# lncRNA HHIP-AS1 Promotes the Osteogenic Differentiation Potential and Inhibits the Migration Ability of Periodontal Ligament Stem Cells

**DOI:** 10.1155/2021/5595580

**Published:** 2021-04-27

**Authors:** Qianyi Qin, Haoqing Yang, Chen Zhang, Xiao Han, Jing Guo, Zhipeng Fan, Jie Guo

**Affiliations:** ^1^Department of Orthodontics, School and Hospital of Stomatology, Cheeloo College of Medicine, Shandong University, Shandong Key Laboratory of Oral Tissue Regeneration, and Shandong Engineering Laboratory for Dental Materials and Oral Tissue Regeneration, 250012, China; ^2^Laboratory of Molecular Signaling and Stem Cells Therapy, Beijing Key Laboratory of Tooth Regeneration and Function Reconstruction, School of Stomatology, Capital Medical University, 100050, China; ^3^The Affiliated Stomatological Hospital of Nanchang University and the Key Laboratory of Oral Biomedicine, Jiangxi Province 330006, China; ^4^Research Unit of Tooth Development and Regeneration, Chinese Academy of Medical Sciences, China

## Abstract

Alveolar bone remodeling under orthodontic force is achieved by periodontal ligament stem cells (PDLSCs), which are sensitive to mechanical loading. How to regulate functions of PDLSCs is a key issue in bone remodeling during orthodontic tooth movement. This study is aimed at investigating the roles of lncRNA Hedgehog-interacting protein antisense RNA 1 (HHIP-AS1) in the functional regulation of PDLSCs. First, HHIP-AS1 expression was downregulated in PDLSCs under continuous compressive pressure. Then, we found that the alkaline phosphatase activity, *in vitro* mineralization, and expression levels of bone sialoprotein, osteocalcin, and osterix were increased in PDLSCs by HHIP-AS1. The results of scratch migration and transwell chemotaxis assays revealed that HHIP-AS1 inhibited the migration and chemotaxis abilities of PDLSCs. In addition, the RNA sequencing data showed that 356 mRNAs and 14 lncRNAs were upregulated, including receptor tyrosine kinase-like orphan receptor 2 and nuclear-enriched abundant transcript 1, while 185 mRNAs and 6 lncRNAs were downregulated, including fibroblast growth factor 5 and LINC00973, in HHIP-AS1-depleted PDLSCs. Bioinformatic analysis revealed several biological processes and signaling pathways related to HHIP-AS1 functions, including the PI3K-Akt signaling pathway and JAK-STAT signaling pathway. In conclusion, our findings indicated that HHIP-AS1 was downregulated in PDLSCs under compressive pressure, and it promoted the osteogenic differentiation potential and inhibited the migration and chemotaxis abilities of PDLSCs. Thus, HHIP-AS1 may be a potential target for accelerating tooth movement during orthodontic treatment.

## 1. Introduction

Orthodontic treatment is a time-consuming process that usually takes 2 to 3 years. Longer treatment cycles are often accompanied by higher risks of complications, such as enamel demineralization, mucosal ulcers, caries, gingival recession, and root resorption [1–7]. It is a common pursuit of orthodontists and patients to accelerate orthodontic tooth movement (OTM) and achieve the treatment goals of health, function, stability, and beauty as quickly as possible. Presently, there are many adjunctive intervention measures to accelerate OTM. These range from surgical interventions to nonsurgical interventions. The surgical interventions include periodontally accelerated osteogenic orthodontics [8], periodontal ligament distraction osteogenesis and dentoalveolar distraction osteogenesis [9], and circumferential supracrestal fiberotomy [10]. The nonsurgical interventions include laser [11], vibration [12], drug [13], ultrasound [14], and electromagnetic therapies [15]. However, the current adjunctive intervention measures are traumatic or cannot accelerate tooth movement completely. Further, none of these measures have been recognized as routine therapies in orthodontic clinics.

During OTM, the force applied to the tooth is transmitted to the alveolar bone through the periodontal ligament (PDL). Research has revealed that compression leads to bone resorption, which is a rate-limiting process, and tension causes bone formation. PDL remodeling, combined with localized resorption and deposition of alveolar bone, enables the tooth to move during orthodontic force application [16, 17]. Furthermore, periodontal ligament stem cells (PDLSCs) with strong self-renewal potential and multiple differentiation potentials can form new bone, cementum, and periodontal ligaments *in vitro* and *in vivo* [18, 19]. PDLSCs are regarded as the seed cells for periodontal tissue reconstruction and remodeling. Additionally, PDLSCs are sensitive to mechanical loading, and the effect of orthodontic force on alveolar bone remodeling is achieved by PDLSCs [20]. Positive signals of PDGFR*α* and nestin in the PDL were gradually enhanced during orthodontic treatment in an OTM rat model and subsequently dropped on the pressure side, indicating that PDLSCs participated in OTM [21]. *In vitro* investigations demonstrated that the application of different types and magnitudes of force stimuli, including tension and compression, could significantly regulate the osteogenic differentiation of PDLSCs [21–23]. Thus, identifying the key targets and regulatory mechanisms underlying the osteogenic differentiation of PDLSCs can be beneficial for bone remodeling under the orthodontic condition.

Long noncoding RNAs (lncRNAs), which are noncoding RNA molecules longer than 200 nt, participate in many important regulatory processes in the body. The mechanisms underlying the action of lncRNAs mainly involve signals, decoys, guides, and scaffolds [24]. According to existing research, lncRNAs regulate gene expression mainly through transcriptional, posttranscriptional, and epigenetic regulation [25–27]. Moreover, lncRNAs can regulate the osteogenic differentiation of PDLSCs in a variety of ways. Some lncRNAs promote the osteogenic differentiation of PDLSCs, while others inhibit this function; all these lncRNAs play a critical role in bone regeneration and remodeling [28]. For instance, lncRNA TUG1 acted as a “sponge” for miR-222-3p to compete with target genes for miR-222-3p binding sites, which inhibited the negative regulation of miR-222-3p on Smad2/7 and promoted the osteogenic differentiation of PDLSCs [29]. Then, downregulation of lncRNA ANCR could activate the canonical Wnt signaling pathway and promote the osteogenesis of PDLSCs [30]. Additionally, lncRNA Hedgehog-interacting protein antisense RNA 1 (HHIP-AS1) is a newly discovered lncRNA located on human chromosome 4 and is mainly expressed in 18 types of tissues [31]. HHIP-AS1 could promote apoptosis and inhibit proliferation, migration, and chemotaxis of liver cancer cells [31]. In our previous study, we found that HHIP-AS1 expression was downregulated during the osteogenic differentiation of bone marrow mesenchymal stem cells (BMSCs) [32]. Furthermore, the expression of HHIP-AS1 in PDLSCs was lower than that in BMSCs [33]. Thus, it was speculated that HHIP-AS1 might be related to the osteogenic differentiation of PDLSCs. However, the role and mechanism of HHIP-AS1 in PDLSCs are still uncertain.

In the present study, we explored the expression of HHIP-AS1 in PDLSCs under compressive pressure and the roles of HHIP-AS1 in the osteogenic differentiation, migration, and chemotaxis of PDLSCs. RNA sequencing (RNA-seq) and bioinformatic analysis were performed to investigate the potential mechanisms of HHIP-AS1. Through this investigation, some original insights which illustrated the potential functions and mechanisms of HHIP-AS1 in PDLSCs were obtained.

## 2. Materials and Methods

### 2.1. Cell Cultures

The PDLSCs used in this study were isolated from human impacted third molar teeth. The study was conducted with the permission of the patients and under the guidance of Beijing Stomatological Hospital, Capital Medical University. First, the teeth were sterilized with 75% ethanol and washed with phosphate-buffered saline (PBS); next, the cells were isolated, cultured, and identified, as previously described [34–36]. Briefly, the periodontal ligaments were digested with a solution containing 3 mg/mL collagenase type I (Sigma-Aldrich, St. Louis, MO, USA) and 4 mg/mL dispase (Roche Diagnostics GmbH, Mannheim, Germany) at 37°C for 60 min after they were separated lightly from the middle one-third of the tooth root. Then, single-cell suspensions were successfully obtained using a 70 mm strainer (Nest, Wuxi, China). The cells were grown in MEM Alpha medium (Gibco, Carlsbad, CA, USA) supplemented with 10,000 units/mL penicillin-streptomycin (Gibco), 200 mM L-glutamine (Gibco), and 10% fetal bovine serum (FBS; Gibco) in an incubator at 37°C and under 5% carbon dioxide. PDLSCs from passages 3–5 were used in the following experiments.

To induce osteogenic differentiation, 2.0 × 10^5^ cells were seeded into each well of 6-well plates. Once the cells reached 80% confluency, the medium was changed to an osteogenic-inducing medium that contained 2 mM *β*-glycerophosphate (Sigma-Aldrich), 100 *μ*M/mL ascorbic acid (Sigma-Aldrich), 10 nM dexamethasone (Sigma-Aldrich), and 1.8 mM KH_2_PO_4_ (Sigma-Aldrich) for a period of up to 14 days.

### 2.2. Plasmid Construction and Viral Infection

The full-length human HHIP-AS1 constructs were created following standard protocols and verified by gene sequencing. The cDNA of HHIP-AS1 was subcloned into the LV5 lentiviral vector (GenePharma Company, Suzhou, China) for overexpression. Subsequently, the LV3 lentiviral vector (GenePharma) was used to construct a short hairpin RNA (shRNA) of HHIP-AS1. The PDLSCs were seeded and cultured in 100 mm dishes overnight and infected with lentiviruses and 6 *μ*g/mL polybrene (Sigma-Aldrich) for 12 h. Then, the transfected PDLSCs were selected by proper antibiotics after 72 h of infection. The target sequences of the shRNAs were as follows: control shRNA (Consh), 5′-TTCTCCGAACGTGTCACGTTTC-3′, and HHIP-AS1 shRNA (HHIP-AS1sh), 5′-GCACCAATGCATCTTGTATGA-3′.

### 2.3. Real-Time Reverse Transcriptase Polymerase Chain Reaction (Real-Time RT-PCR)

RNA extraction, cDNA synthesis, and real-time RT-PCR were performed as described previously [37]. Total RNA was extracted from PDLSCs using TRIzol reagent (Invitrogen, Carlsbad, CA, USA), after which cDNAs were synthesized by 1 *μ*g aliquots of RNA with oligo (dT) with the use of random primers and reverse transcriptase, according to the manufacturer's protocol (Invitrogen). The cDNA amplifications were conducted using the QuantiTect SYBR Green PCR Kit (Qiagen, Hilden, Germany) and an Icycler iQ Multicolor Real-Time PCR Detection System. All samples were tested in triplicate. The 2^−*ΔΔ*CT^ method was used to determine the expression level of each gene, which was normalized to that of GAPDH. The primers used in this study are listed in Table S1.

### 2.4. Western Blot Analysis

Total protein was extracted from PDLSCs using RIPA lysis buffer. The Western blot was performed as previously described [35]. Equal amounts of protein (25 *μ*g) were prepared for gel electrophoresis. The samples were electrophoresed on 10% sodium dodecyl sulfate-polyacrylamide gels. They were then transferred to polyvinylidene difluoride membranes using a semidry electrophoretic transfer apparatus (Bio-Rad Laboratories, Hercules, CA, USA). The membranes were blocked with 5% nonfat milk for 1 h and subsequently incubated overnight with primary antibodies at 4°C. Then, the membranes were washed three times with Tris-buffered saline with Tween and incubated with horseradish peroxidase-conjugated secondary antibodies (Promega, Madison, WI, USA). After this, bands were visualized using enhanced chemiluminescence detection reagents (Applygen, Beijing, China). GAPDH served as an internal control. We used the following primary antibodies: bone sialoprotein (BSP; Cat No. bs-2668R, Bioss, Beijing, China), osteocalcin (OCN; Cat No. bs-0470R, Bioss), osterix (OSX; Cat No. bs-1110R, Bioss), and GAPDH (Cat No. 60004-1-Ig; Proteintech, Rosemont, IL, USA).

### 2.5. Continuous Compressive Pressure Assay

First, round glass panes (with a diameter of 30 mm, a thickness of 4 mm, and a density of 2.5 × 10^3^ kg/m^3^) were customized and sterilized. PDLSCs were seeded at a cell density of 2.0 × 10^5^ and cultured in each well of 6-well plates. Then, we placed a glass pane on the PDLSCs in a single well for 0, 2, 4, and 6 h to subject the PDLSCs to a continuous compressive pressure of 1 g/cm^2^. The control group was treated with 0 h of pressure.

### 2.6. Alkaline Phosphatase (ALP) Activity Assay and Alizarin Red Staining

After 5 days of osteogenic induction, ALP activity was quantified using an ALP activity kit (Sigma-Aldrich) according to the manufacturer's protocol. Then, after 14 days of osteogenic induction, the cells were fixed with 70% ethanol and stained with 2% Alizarin Red (Sigma-Aldrich) to assess mineralization. Next, the cells were destained with 10% cetylpyridinium chloride for approximately 30 min to quantify the calcium levels. The OD values were measured at 562 nm absorbance on a multiplate reader and then compared with a standard calcium curve to determine the calcium concentrations.

### 2.7. Scratch Migration Assay

PDLSCs were seeded into each well of 6-well plates at a 2.0 × 10^5^ cell density. A 1000 *μ*L pipette tip was used to make scratches along the diameters of the cell layers. After washing the wells with PBS, the routine medium was replaced with serum-free medium. Images were captured after 0, 24, and 48 h using a microscope (Olympus) at ×40 magnification. The relative widths of the wounds were calculated by Image-Pro v1.49 (National Institutes of Health).

### 2.8. Transwell Chemotaxis Assay

Transwell chambers (Corning Costar, MA, USA) with 8 *μ*m pore size membranes were applied. In the upper chambers, 2.0 × 10^4^ cells in 100 *μ*L serum-free medium were distributed, and in the bottom chambers, 600 *μ*L MEM Alpha medium with 10% FBS was contained. After 48 h, the cells in the bottom chambers were fixed using 4% paraformaldehyde and stained using a 0.1% crystal violet staining solution. Finally, the numbers of transferred cells in randomly chosen fields were counted with the assistance of a microscope (Olympus) at ×200 magnification.

### 2.9. Bioinformatic Analysis of RNA-seq

HHIP-AS1-depleted PDLSCs were used for RNA-seq. Total RNA was extracted using TRIzol reagent (Invitrogen) according to the manufacturer's instructions. Subsequently, RNA-seq and bioinformatic analysis were carried out by Guangzhou Epibiotek Co., Ltd. China. RNA libraries were constructed by the Epi™ mini long RNA-seq kit, and the Illumina NovaSeq 6000 was used as the instrument model. The DESeq2 algorithm was applied to filter the differentially expressed genes, following the significant analysis and false discovery rate (FDR) analysis under the following criteria: ∣log_2_FC | >1 (fold change > 2), FDR < 0.05. Additionally, GO analysis was performed to explain the biological functions of the differentially expressed genes. The GO annotations were downloaded from Gene Ontology (http://www.geneontology.org/), NCBI (http://www.ncbi.nlm.nih.gov/), and UniProt (http://www.uniprot.org/) [38]. Pathway analysis was conducted to identify the significant pathways of the differentially expressed genes in accordance with the KEGG database [39]. Fisher's exact test was used to identify significant GO categories and pathways. Significance was determined by the *P* value and FDR.

### 2.10. Statistical Analysis

Data were analyzed by the SPSS version 22 statistical software. Statistical significance, which was set at *P* ≤ 0.05, was determined by one-way ANOVA or Student's *t*-test. All experiments were performed independently three times.

## 3. Results

### 3.1. HHIP-AS1 Expression Was Downregulated in PDLSCs under Continuous Compressive Pressure

**Figure 1:**
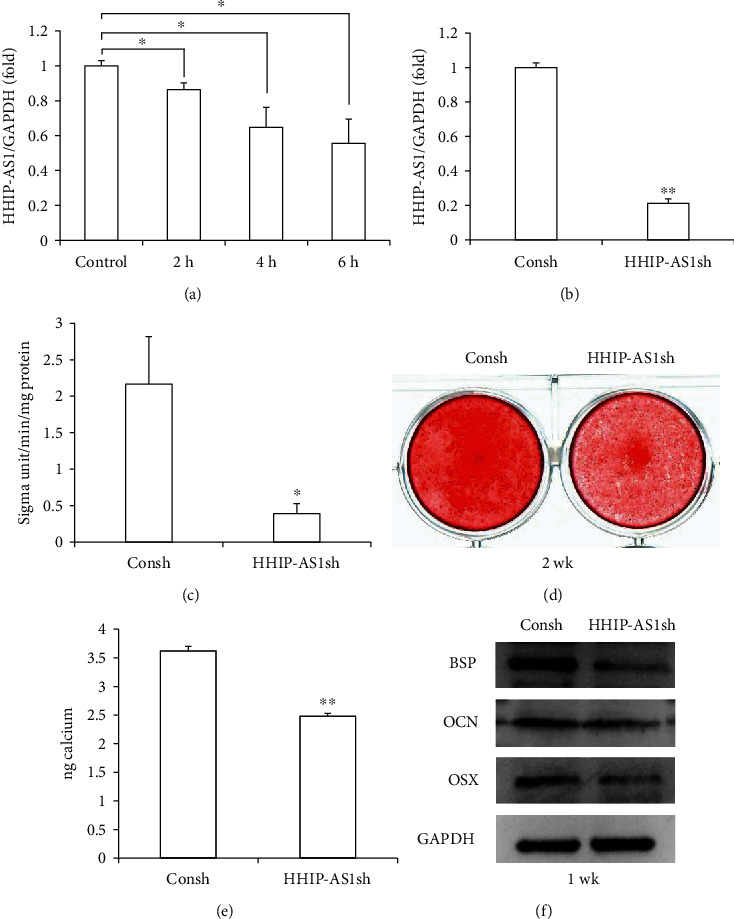
HHIP-AS1 knockdown inhibited the osteogenic differentiation potential of PDLSCs. (a) Real-time RT-PCR result showed the expression level of HHIP-AS1 at 0, 2, 4, and 6 h under continuous compressive pressure. (b) Real-time RT-PCR result showed the knockdown efficiency of HHIP-AS1 in PDLSCs. (c) ALP activity after 5 days of osteogenic induction. (d) Alizarin Red staining result after 2 weeks of osteogenic induction. (e) Calcium quantitative analysis. (f) Western blot results displayed expressions of BSP, OCN, and OSX after 1 week of osteogenic induction. GAPDH was used as internal control in real-time RT-PCR and Western blot. One-way ANOVA or Student's *t*-test was performed to determine statistical significance. All error bars represent the SD (*n* = 3). ^∗^*P* ≤ 0.05; ^∗∗^*P* ≤ 0.01.

First, we evaluated the change in HHIP-AS1 expression in PDLSCs under compressive force, which imitated the mechanical condition during OTM. Real-time RT-PCR result demonstrated that HHIP-AS1 was downregulated in PDLSCs at 2, 4, and 6 h under continuous compressive pressure compared to that in PDLSCs under no pressure (Figure 1(a)).

### 3.2. HHIP-AS1 Promoted the Osteogenic Differentiation Potential of PDLSCs

To further investigate the function of HHIP-AS1 in the osteogenic differentiation of PDLSCs, the HHIP-AS1 shRNA lentivirus was used to knock down HHIP-AS1 expression in PDLSCs. The transfected PDLSCs were treated with 2 *μ*g/mL puromycin for 3 days. The real-time RT-PCR result revealed that HHIP-AS1 was efficiently silenced in PDLSCs (Figure 1(b)). Compared with that in the control group (Consh group), the result of the ALP activity assay indicated that HHIP-AS1 knockdown reduced ALP activity after osteogenic induction for 5 days (Figure 1(c)). Additionally, the mineralization of PDLSCs after 2 weeks of induction was inhibited by HHIP-AS1 depletion compared to that in the Consh group. This was tested by the Alizarin Red staining and calcium quantitative measurement (Figures 1(d) and 1(e)). Furthermore, the Western blot experiments revealed the downregulation of osteogenic-related genes, including BSP and OCN, in HHIP-AS1-depleted PDLSCs compared to that in the Consh group after osteogenic induction for 1 week (Figure 1(f)). The Western blot result also showed that the expression of OSX, a key transcription factor, was significantly decreased in HHIP-AS1-depleted PDLSCs (Figure 1(f)).

**Figure 2:**
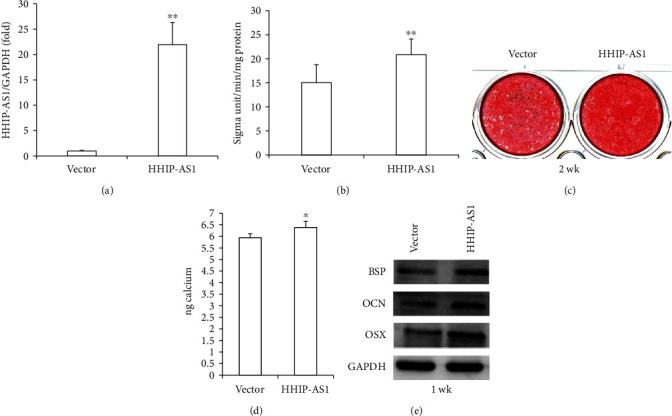
HHIP-AS1 overexpression promoted the osteogenic differentiation potential of PDLSCs. (a) Real-time RT-PCR result showed the overexpression efficiency of HHIP-AS1 in PDLSCs. (b) ALP activity after 5 days of osteogenic induction. (c) Alizarin Red staining result after 2 weeks of osteogenic induction. (d) Calcium quantitative analysis. (e) Western blot results displayed expressions of BSP, OCN, and OSX after 1 week of osteogenic induction. GAPDH was used as internal control in real-time RT-PCR and Western blot. Student's *t*-test was performed to determine statistical significance. All error bars represent the SD (*n* = 3). ^∗^*P* ≤ 0.05; ^∗∗^*P* ≤ 0.01.

Subsequently, PDLSCs were transfected with HHIP-AS1 and an empty vector. After selecting the cells with 2 *μ*g/mL puromycin, the overexpression efficiency of HHIP-AS1 was confirmed by real-time RT-PCR (Figure 2(a)). After 5 days of osteogenic induction, ALP activity was enhanced in PDLSCs by the overexpression of HHIP-AS1 compared with that in the control group (Vector group) (Figure 2(b)). Next, the mineralization of PDLSCs was promoted by HHIP-AS1 overexpression compared with that in the Vector group after 2 weeks of induction (Figures 2(c) and 2(d)). Furthermore, the expressions of BSP, OCN, and OSX were significantly upregulated after 1 week of induction in HHIP-AS1-overexpressed PDLSCs (Figure 2(e)).

### 3.3. HHIP-AS1 Inhibited the Migration and Chemotaxis Abilities of PDLSCs

**Figure 3:**
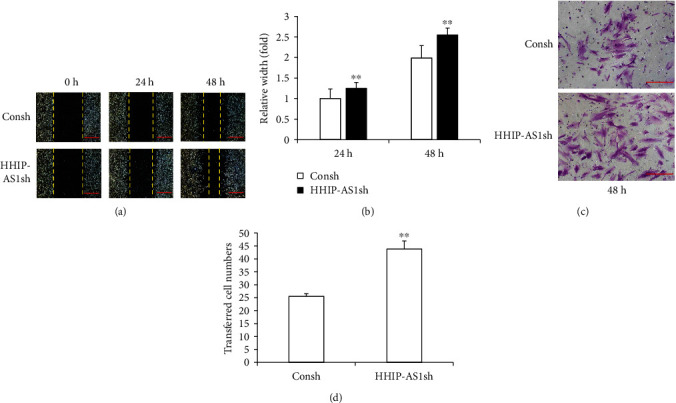
HHIP-AS1 knockdown promoted the migration and chemotaxis abilities of PDLSCs. The results of the (a) scratch migration assay and (b) quantitative analysis at 24 and 48 h (scale bars: 1 mm). The results of the (c) transwell chemotaxis assay and (d) quantitative analysis at 48 h (scale bars: 200 *μ*m). Student's *t*-test was performed to determine statistical significance. All error bars represent the SD (*n* = 9). ^∗∗^*P* ≤ 0.01.

Next, we explored the effect of HHIP-AS1 on cell migration and chemotaxis. The greater migration ability of HHIP-AS1-depleted PDLSCs than that of the Consh group at 24 and 48 h was confirmed by the scratch migration assay and quantitative analysis (Figures 3(a) and 3(b)). Furthermore, the results of the transwell chemotaxis assay and quantitative analysis also revealed a stronger chemotaxis ability of HHIP-AS1-depleted PDLSCs than that of the Consh group at 48 h (Figures 3(c) and 3(d)).

**Figure 4:**
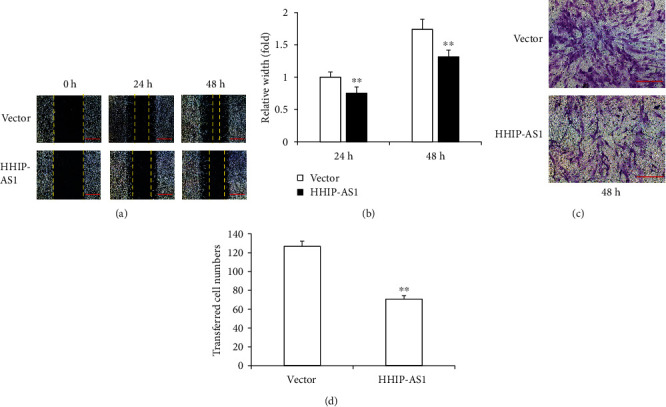
HHIP-AS1 overexpression inhibited the migration and chemotaxis abilities of PDLSCs. The results of the (a) scratch migration assay and (b) quantitative analysis at 24 and 48 h (scale bars: 1 mm). The results of the (c) transwell chemotaxis assay and (d) quantitative analysis at 48 h (scale bars: 200 *μ*m). Student's *t*-test was performed to determine statistical significance. All error bars represent the SD (*n* = 9). ^∗∗^*P* ≤ 0.01.

In contrast, the scratch migration assay and quantitative analysis results showed that HHIP-AS1 overexpression inhibited the migration ability of PDLSCs at 24 and 48 h (Figures 4(a) and 4(b)). Meanwhile, the results of the transwell chemotaxis assay and quantitative analysis revealed that HHIP-AS1 overexpression suppressed the chemotaxis ability at 48 h (Figures 4(c) and 4(d)).

### 3.4. The Profiling of Differentially Expressed mRNAs and lncRNAs in HHIP-AS1-Depleted PDLSCs

The differentially expressed mRNAs and lncRNAs in HHIP-AS1-depleted PDLSCs were identified by RNA-seq. In total, 541 differentially expressed protein-coding genes were detected (∣log_2_FC | >1, FDR < 0.05); among these, 356 were upregulated and 185 were downregulated in HHIP-AS1-depleted PDLSCs compared to that in the Consh group (Table S2). Additionally, 20 differentially expressed lncRNAs were also detected (∣log_2_FC | >1, FDR < 0.05); among these, 14 were upregulated and 6 were downregulated in HHIP-AS1-depleted PDLSCs compared to that in the Consh group (Table S3).

**Figure 5:**
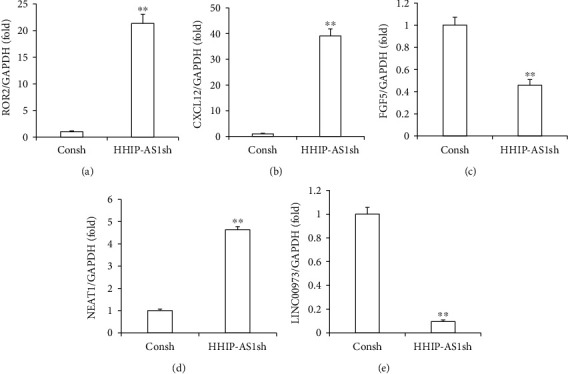
HHIP-AS1 knockdown resulted in upregulation of ROR2, CXCL12, and NEAT1 and downregulation of FGF5 and LINC00973 in PDLSCs. Real-time RT-PCR results showed the expressions of (a) ROR2, (b) CXCL12, (c) FGF5, (d) NEAT1, and (e) LINC00973. GAPDH was used as internal control. Student's *t*-test was performed to determine statistical significance. All error bars represent the SD (*n* = 3). ^∗∗^*P* ≤ 0.01.

To ensure the reliability of the RNA-seq data, three differentially expressed mRNAs (receptor tyrosine kinase-like orphan receptor 2 (ROR2), C-X-C motif chemokine ligand 12 (CXCL12), and fibroblast growth factor 5 (FGF5)) and two differentially expressed lncRNAs (nuclear-enriched abundant transcript 1 (NEAT1) and LINC00973) were randomly chosen for expression detection by real-time RT-PCR. Real-time RT-PCR results confirmed that HHIP-AS1 knockdown resulted in the upregulation of ROR2 (Figure 5(a)), CXCL12 (Figure 5(b)), and NEAT1 (Figure 5(d)) and downregulation of FGF5 (Figure 5(c)) and LINC00973 (Figure 5(e)) in PDLSCs. Furthermore, we repeated the continuous compressive pressure assay and found that when PDLSCs were stimulated by continuous compressive pressure, ROR2 and CXCL12 were both significantly upregulated and FGF5 was significantly downregulated at 2 h under pressure (Figure S1).

### 3.5. Bioinformatic Analysis of RNA-seq Data

**Figure 6:**
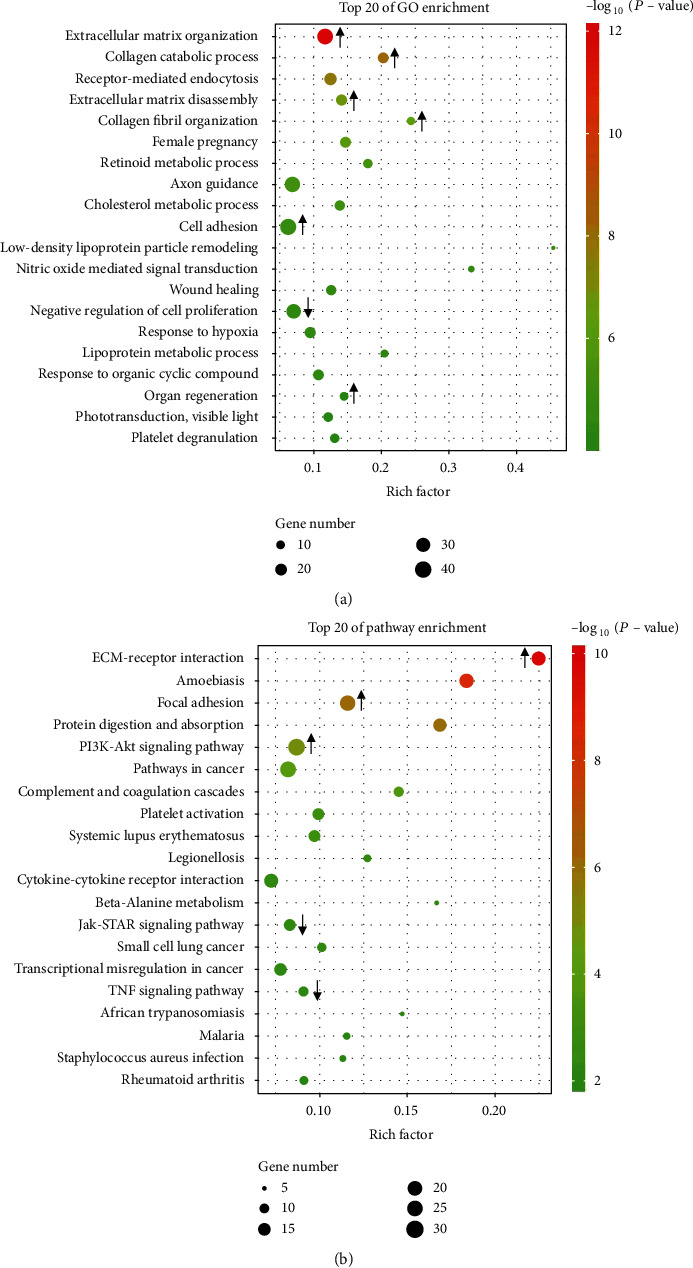
GO and pathway analysis of RNA-seq data. (a) Bubble diagram of the top 20 enriched GO terms of the differentially expressed genes in HHIP-AS1-depleted PDLSCs. (b) Bubble diagram of the top 20 enriched pathway terms of the differentially expressed genes in HHIP-AS1-depleted PDLSCs. The arrows mark the changes of key GO terms and signaling pathways.

First, GO analysis was conducted from three aspects: biological process, molecular function, and cellular component (Figure S2). For biological processes, we obtained 535 upregulated and 360 downregulated GO functions (*P* < 0.05) (Table S4). The relevance between gene expression and biological processes was represented by the negative logarithm of the *P* value (−LgP). The bubble diagram clearly illustrated the top 20 enriched GO terms (Figure 6(a)). Among these enriched terms, some important upregulated GO functions that might be related to osteogenic differentiation included extracellular matrix (ECM) organization, cell adhesion, and skeletal system development, while some important downregulated GO functions that might be related to osteogenic differentiation included DNA methylation on cytosine, regulation of cell proliferation, and cell chemotaxis (Table S4).

Next, the significantly altered pathways that connected the differentially expressed genes with biological functions were identified by KEGG analysis. Twenty-four upregulated pathways and 20 downregulated pathways were detected, which may play critical roles during HHIP-AS1 regulation (*P* < 0.05) (Table S5, Figure S3). The top 20 enriched pathways were depicted as the bubble diagram (Figure 6(b)). Among these signaling pathways, the upregulated pathways that correlated to osteogenic differentiation included the PI3K-Akt signaling pathway, calcium signaling pathway, and Hedgehog signaling pathway. Additionally, the downregulated pathways that correlated to osteogenic differentiation were the TNF signaling pathway, JAK-STAT signaling pathway, and cell cycle (Table S5).

## 4. Discussion

To assist alveolar bone remodeling during OTM, it is necessary to investigate the regulation and related mechanisms of PDLSCs under mechanical stimuli. In the present study, PDLSCs were subjected to continuous compressive pressure at 1 g/cm^2^ for 2, 4, and 6 h to imitate the compressive condition during OTM. We found that HHIP-AS1 was downregulated in PDLSCs under compressive pressure. A study showed that after the application of compressive force at 1 g/cm^2^ for 12 and 24 h, PDLSCs suffered an elongated morphology, and the osteogenic marker type I collagen (Col-I) was suppressed in PDLSCs. However, the morphology and Col-I expression were recovered after force withdrawal [40]. Thus, the compressive pressure caused the downregulation of HHIP-AS1, which might further lead to the inhibition of osteogenic differentiation. To verify this hypothesis, the function of HHIP-AS1 in the osteogenic differentiation of PDLSCs was explored. Initially, we found that HHIP-AS1 knockdown inhibited ALP activity, an early-stage marker of osteogenic differentiation, and *in vitro* mineralization, a late-stage marker of osteogenic differentiation. We further revealed that HHIP-AS1 overexpression promoted ALP activity and *in vitro* mineralization. Moreover, HHIP-AS1 also upregulated the expressions of osteogenic differentiation markers, including BSP and OCN, and the transcription factor, OSX, in PDLSCs. These results suggested that HHIP-AS1 enhanced the osteogenic differentiation of PDLSCs, and downregulation of HHIP-AS1 led to the inhibition of osteogenic differentiation.

In addition to the mineralization ability, we further investigated the roles of HHIP-AS1 in the migration and chemotaxis abilities of PDLSCs. The results of the scratch migration assay, transwell chemotaxis assay, and quantitative analysis illustrated that HHIP-AS1 knockdown promoted the migration and chemotaxis abilities of PDLSCs and that HHIP-AS1 overexpression inhibited the migration and chemotaxis abilities. This was consistent with HHIP-AS1 function in the previous research on liver cancer cells [31]. Moreover, it has been reported that the application of an external force can disturb the balance between the synthesis and degradation of periodontal tissues, and the activated remodeling of PDL and alveolar bone leads to tooth movement. On the resorption side, the PDL and alveolar bone are degraded to provide space for tooth movement [17]. Taken together, our findings indicated that on the compressive side, downregulation of HHIP-AS1 led to the reduction of bone formation and enhanced migration and chemotaxis abilities of PDLSCs, which promoted the movement of PDLSCs and thereby accelerated tooth movement. Further investigation will be carried on in an animal model to validate these findings.

Moreover, to identify the mechanism of HHIP-AS1 action, RNA-seq and subsequent bioinformatic analysis were carried out. RNA-seq identified 541 differentially expressed mRNAs and 20 differentially expressed lncRNAs in HHIP-AS1-depleted PDLSCs. Among these, 356 mRNAs and 14 lncRNAs were upregulated, including ROR2 and NEAT1, while 185 mRNAs and 6 lncRNAs were downregulated, including FGF5 and LINC00973. Furthermore, the expression of ROR2, CXCL12, FGF5, NEAT1, and LINC00973, as tested by real-time RT-PCR, confirmed the RNA-seq results. ROR2, belonging to a family of receptor tyrosine kinases, could initiate the commitment of mesenchymal stem cells (MSCs) to the osteoblastic lineage and promote differentiation at both early and late stages of osteoblastogenesis. Using a mouse calvaria *ex vivo* organ culture model, it was demonstrated that these effects of ROR2 resulted in increased bone formation [41]. However, this was not consistent with our findings that HHIP-AS1 knockdown promoted the expression of ROR2 and inhibited the osteogenic differentiation. Meanwhile, CXCL12, a chemokine, influenced the migration, growth, survival, and differentiation of MSCs. The CXCL12/CXCR4 axis played an important role in the development and maintenance of the skeletal system through the recruitment of multipotent MSCs for bone regeneration [42]. Hwang et al. [43] treated the critical size defects of mouse calvaria with CXCL12 and bone morphogenetic protein 2 (BMP2). The highest degree of new bone regeneration was shown by the strong osteogenic differentiation and enhanced cell migration effects. In our study, although HHIP-AS1 depletion caused the upregulation of CXCL12, which promoted migration and chemotaxis, the suppression of osteogenesis was not consistent with the function of CXCL12. Then, NEAT1, a recently identified lncRNA, promoted the osteogenic differentiation of BMSCs by regulating the miR-29b-3p/BMP1 axis; this indicated its potential role in bone metabolism was contrary to our results [44]. Interestingly, we found that ROR2 and CXCL12 were both significantly upregulated in PDLSCs stimulated by continuous compressive pressure. Sometimes, the gene plays different roles in different cells or tissues under different conditions. ROR2, CXCL12, and NEAT1 might play a different role in PDLSCs during OTM. Further studies will be carried on to test this hypothesis. Additionally, the expression of FGF5 was reduced in HHIP-AS1-depleted PDLSCs and it was also significantly downregulated in PDLSCs stimulated by continuous compressive pressure. A study revealed that FGF5 facilitated cell proliferation through extracellular signal-regulated kinase 1/2 activation and enhanced the osteogenic differentiation of tonsil-derived MSCs [45]. Thus, we speculated that FGF5 played an important role in mediating the HHIP-AS1 function. Furthermore, the expressions of secreted phosphoprotein 1 (SPP1) and spondin 1 (SPON1) were upregulated in HHIP-AS1-depleted PDLSCs. SPP1 was demonstrated to be a factor for triggering an osteopenic state caused by the mechanical load during tooth movement [46, 47]. Meanwhile, SPON1 was upregulated under compression and was confirmed as a negative regulator of bone mass [48, 49]. These results indicated that SPP1 and SPON1 might also be crucial downstream genes of HHIP-AS1 that could have significant impacts on the regulation of PDLSCs and should be explored in further investigations.

Furthermore, various biological processes, ranging from the development of individuals to the occurrence of diseases, have been shown to be affected by lncRNAs [50, 51]. To clarify the underlying mechanism of HHIP-AS1 in PDLSCs, we performed GO analysis to explore the biological processes in which differentially expressed mRNAs are involved. The results showed that ECM organization, cell adhesion, skeletal system development, DNA methylation on cytosine, regulation of cell proliferation, and cell chemotaxis were the top GO terms and were related to HHIP-AS1 regulation in PDLSCs. Moreover, pathway analysis revealed that the PI3K/Akt signaling pathway, calcium signaling pathway, and Hedgehog signaling pathway were upregulated, while the TNF signaling pathway, JAK-STAT signaling pathway, and cell cycle were downregulated in HHIP-AS1-depleted PDLSCs, which were also related to HHIP-AS1 regulation in PDLSCs. Intensive studies have elucidated that many critical signaling pathways participate in regulating the osteogenic differentiation of MSCs, and multiple signaling pathways usually interact with each other to form a complex regulatory network. In our study, HHIP-AS1 depletion suppressed the osteogenic differentiation potential and upregulated the PI3K/Akt signaling pathway. Similarly, miR-let-7c-5p inhibited the expression of HMGA2 and PI3K/Akt signaling pathway and promoted the osteogenic differentiation of dental pulp stem cells in the lipopolysaccharide-mediated inflammatory environment [52]. Also, HHIP-AS1 depletion led to the downregulation of the JAK-STAT signaling pathway. A recent study showed that BMSCs promoted the healing of rabbit tibial fractures through the JAK-STAT signaling pathway [53]. These results indicated that the PI3K/Akt and JAK-STAT signaling pathways were key pathways for HHIP-AS1 regulation in PDLSCs. In general, HHIP-AS1 had a significant effect on the biological functions of PDLSCs by regulating downstream genes and related pathways. However, the underlying molecular mechanisms remain to be further explored.

## 5. Conclusions

In conclusion, we found here that HHIP-AS1 was downregulated in PDLSCs under compressive pressure. Additionally, HHIP-AS1 promoted the osteogenic differentiation potential and inhibited the migration and chemotaxis abilities of PDLSCs. Bioinformatic analysis of RNA-seq data revealed candidate downstream genes of HHIP-AS1 and key signaling pathways related to HHIP-AS1 regulation. Overall, our findings provide a potential target as well as a theoretical basis for the functional modification of PDLSCs and bone remodeling under the mechanical condition of orthodontic treatment.

## Data Availability

The data used to support the findings of this study are included within the article.

## Supplementary Materials

Supplementary 1 Table S1: primer sequences used in the real-time RT-PCR.

Supplementary 2 Table S2: the differentially expressed mRNAs in HHIP-AS1-depleted PDLSCs.

Supplementary 3 Table S3: the differentially expressed lncRNAs in HHIP-AS1-depleted PDLSCs.

Supplementary 4 Figure S1: ROR2 and CXCL12 were upregulated while FGF5 was downregulated under compressive pressure. Real-time RT-PCR results showed expressions of ROR2 (a), CXCL12 (b), and FGF5 (c) at 2 h under continuous compressive pressure. GAPDH was used as internal control. Student's *t*-test was performed to determine statistical significance. All error bars represent the SD (*n* = 3). ^∗^*P* ≤ 0.05; ^∗∗^*P* ≤ 0.01.

Supplementary 5 Figure S2: significant GO analysis of differentially expressed genes from aspects of biological process, molecular function, and cellular component. (a) Upregulated GO terms. (b) Downregulated GO terms.

Supplementary 6 Table S4: the significant upregulated and downregulated GO functions in HHIP-AS1-depleted PDLSCs.

Supplementary 7 Table S5: the significant upregulated and downregulated pathways in HHIP-AS1-depleted PDLSCs.

Supplementary 8 Figure S3: significant pathway analysis of differentially expressed genes in HHIP-AS1-depleted PDLSCs. (a) Upregulated pathways. (b) Downregulated pathways.
