# Supplementary material for: lncRNA HHIP-AS1 Promotes the Osteogenic Differentiation Potential and Inhibits the Migration Ability of Periodontal Ligament Stem Cells
Source: Stem Cells Int. 2021 Apr 27;2021:5595580. doi: 10.1155/2021/5595580 (PMC8554619; doi:10.1155/2021/5595580)
Supplement: Supplementary 1 — Table S1: primer sequences used in the real-time RT-PCR. [file 5595580.f1.pdf]

Supplementary Table 1. Primer sequences used in the real-time RT-PCR.

Gene Symbol	Primer Sequences (5'-3')
GAPDH-F	CGGACCAATACGACCAAATCCG
GAPDH-R	AGCCACATCGCTCAGACACC
HHIP-AS1-F	GCTAAACCGACAGAGTGGTGA
HHIP-AS1-R	AGTCCTGGAGAGGGTTGCTC
ROR2-F	CAGCAAGATGGGGATTCTGT
ROR2-R	GCACATGCAAACCAAGAAGA
CXCL12-F	AGAGCCAACGTCAAGCATCT
CXCL12-R	CTTTAGCTTCGGGTCAATGC
FGF5-F	TTTCTGCCAAGATTCAAGCA
FGF5-R	AGGTGCAGAAAGGGGAATCT
NEAT1-F	GGGCCATCAGCTTTGAATAA
NEAT1-R	CTTGAAGCAAGGTTCCAAGC
LINC00973-F	CAGCTGTGTTACTCCTTCGC
LINC00973-R	AGCCAGAGATCAGGGTTGAC
