# Supplementary material for: lncRNA HHIP-AS1 Promotes the Osteogenic Differentiation Potential and Inhibits the Migration Ability of Periodontal Ligament Stem Cells
Source: Stem Cells Int. 2021 Apr 27;2021:5595580. doi: 10.1155/2021/5595580 (PMC8554619; doi:10.1155/2021/5595580)
Supplement: Supplementary 2 — Table S2: the differentially expressed mRNAs in HHIP-AS1-depleted PDLSCs. [file 5595580.f2.pdf]

Supplementary Table 2. The differentially expressed mRNAs in HHIP-AS1-depleted PDLSCs.

external_gene_name	log2FC	P value	FDR	Style	gene_biotype
TF	8.10339	1.88E-10	7.19E-09	up	protein_coding
LYZ	7.87192	9.46E-10	3.31E-08	up	protein_coding
AHSG	7.62963	1.28E-09	4.37E-08	up	protein_coding
ALB	7.50654	7.42E-09	2.26E-07	up	protein_coding
ITIH2	7.22841	6.56E-08	1.66E-06	up	protein_coding
APOH	7.15778	1.35E-08	3.91E-07	up	protein_coding
SERPINA1	7.1035	5.63E-11	2.34E-09	up	protein_coding
APOA2	6.82945	9.16E-08	2.23E-06	up	protein_coding
APOB	6.67813	4.63E-22	5.58E-20	up	protein_coding
APOA1	6.56511	5.03E-07	1.07E-05	up	protein_coding
FGA	6.55842	2.56E-06	4.72E-05	up	protein_coding
PGC	6.0953	1.49E-05	0.000224721	up	protein_coding
FGG	5.93528	6.31E-05	0.000808704	up	protein_coding
AGT	5.71486	3.57E-05	0.00049193	up	protein_coding
AMBP	5.4264	3.23E-06	5.86E-05	up	protein_coding
SERPINF2	5.32625	0.000159978	0.001791279	up	protein_coding
PTGIS	4.96258	3.04E-224	4.58E-220	up	protein_coding
COL21A1	4.67585	4.79E-09	1.50E-07	up	protein_coding
ADH1B	4.62265	2.53E-37	7.06E-35	up	protein_coding
SPP1	4.54333	6.68E-06	0.000111952	up	protein_coding
NR0B1	4.47045	1.27E-17	1.08E-15	up	protein_coding
AFP	4.469	2.07E-13	1.18E-11	up	protein_coding
KIF26B	4.36285	2.56E-64	3.22E-61	up	protein_coding
EPHA3	4.35947	2.07E-22	2.58E-20	up	protein_coding
ROR2	4.20943	4.99E-38	1.50E-35	up	protein_coding
ADH1C	4.16089	0.000923083	0.007907643	up	protein_coding
RASL12	4.15169	0.000109802	0.001290013	up	protein_coding
ERVMER34-1	4.01765	0.00177526	0.013535088	up	protein_coding
GPC3	3.97292	0.000253096	0.002641455	up	protein_coding
ALPL	3.90592	0.004158874	0.026766084	up	protein_coding
A2M	3.81852	9.13E-05	0.001114109	up	protein_coding
CXCL12	3.80744	2.34E-15	1.67E-13	up	protein_coding
BMP6	3.80045	0.000441273	0.00424637	up	protein_coding
RELN	3.71344	9.67E-05	0.001163035	up	protein_coding
STEAP4	3.66243	4.36E-09	1.37E-07	up	protein_coding
MMP28	3.64173	1.57E-06	3.03E-05	up	protein_coding
GMNC	3.61217	0.000263356	0.002733388	up	protein_coding
MOB3B	3.60223	0.001529682	0.011973497	up	protein_coding
GCNT3	3.4917	4.53E-12	2.16E-10	up	protein_coding
LAMP3	3.36481	0.000182151	0.001996501	up	protein_coding
ADGRD1	3.30157	0.001565285	0.012195132	up	protein_coding
PDGFD	3.11519	1.57E-22	1.98E-20	up	protein_coding
COL11A1	3.10689	4.45E-104	1.34E-100	up	protein_coding
OLFML2A	3.10256	6.25E-28	1.19E-25	up	protein_coding
NANOS1	3.09904	5.75E-17	4.66E-15	up	protein_coding
RASL11B	3.07891	1.27E-06	2.50E-05	up	protein_coding
IL16	3.03323	4.30E-16	3.23E-14	up	protein_coding
ABCA13	3.01587	1.43E-15	1.04E-13	up	protein_coding
PPARGC1A	3.00621	2.24E-19	2.25E-17	up	protein_coding
ST8SIA4	2.99644	4.90E-09	1.53E-07	up	protein_coding
FER1L6	2.98502	5.10E-47	2.40E-44	up	protein_coding
RGCC	2.90002	5.37E-43	2.07E-40	up	protein_coding
MLXIPL	2.8876	0.003452722	0.023149927	up	protein_coding
ERBB3	2.84709	0.000915141	0.007853005	up	protein_coding
PLXDC1	2.84068	0.001122921	0.009307205	up	protein_coding
SEL1L3	2.82079	3.53E-06	6.33E-05	up	protein_coding
ABCC6	2.79914	9.49E-05	0.001147723	up	protein_coding
GNAL	2.79197	8.64E-06	0.000141642	up	protein_coding
RASD1	2.77926	2.12E-09	6.93E-08	up	protein_coding
FAM107A	2.73823	2.95E-38	9.07E-36	up	protein_coding
ACKR2	2.73157	0.000195614	0.002119775	up	protein_coding
IDO1	2.72508	1.76E-06	3.36E-05	up	protein_coding
SNCAIP	2.72034	4.42E-06	7.73E-05	up	protein_coding
RBP4	2.69863	0.001139172	0.009421155	up	protein_coding

ALDH3A1	2.65759	3.88E-17	3.20E-15	up	protein_coding
SEMA4A	2.63277	0.001250178	0.010155165	up	protein_coding
GBP4	2.62393	0.0017184	0.013207778	up	protein_coding
KMO	2.59028	2.56E-05	0.000368284	up	protein_coding
PTPRO	2.59021	1.03E-05	0.000165638	up	protein_coding
LBH	2.57671	5.75E-09	1.78E-07	up	protein_coding
FNDC1	2.53682	0.00041065	0.004002836	up	protein_coding
FPR1	2.52632	0.002065491	0.015255664	up	protein_coding
COL5A3	2.50847	7.82E-56	6.54E-53	up	protein_coding
DAAM2	2.48868	1.32E-37	3.74E-35	up	protein_coding
NDST3	2.48475	0.002399485	0.017281802	up	protein_coding
MTUS1	2.46184	3.82E-11	1.62E-09	up	protein_coding
ACKR3	2.44167	0.000120822	0.001399671	up	protein_coding
PHKG1	2.3885	0.001634185	0.01267946	up	protein_coding
SPON1	2.37757	2.80E-40	9.36E-38	up	protein_coding
KLHL41	2.3542	0.000174878	0.001935097	up	protein_coding
COL3A1	2.34093	7.07E-173	5.32E-169	up	protein_coding
SCARA5	2.33923	0.001139977	0.009422646	up	protein_coding
C2	2.3392	0.003293328	0.022331168	up	protein_coding
CES3	2.32807	0.001595584	0.012411929	up	protein_coding
B3GALT4	2.3047	1.02E-08	3.03E-07	up	protein_coding
SLITRK1	2.29301	0.000988106	0.008342467	up	protein_coding
FHDC1	2.28659	4.12E-07	8.89E-06	up	protein_coding
PPL	2.25887	1.61E-19	1.65E-17	up	protein_coding
TMEM155	2.23775	6.82E-05	0.000866354	up	protein_coding
COL14A1	2.23078	1.40E-27	2.60E-25	up	protein_coding
SNED1	2.22311	2.83E-121	1.07E-117	up	protein_coding
FAM160A1	2.22165	4.59E-14	2.80E-12	up	protein_coding
RGMA	2.21106	1.33E-09	4.53E-08	up	protein_coding
TSPAN11	2.1971	2.66E-08	7.29E-07	up	protein_coding
LRP5	2.18718	7.52E-50	4.36E-47	up	protein_coding
DDIT4L	2.1846	0.004918896	0.030789102	up	protein_coding
GLDN	2.15708	1.86E-09	6.15E-08	up	protein_coding
SLC1A3	2.14695	5.23E-43	2.07E-40	up	protein_coding
CRISPLD1	2.13162	5.66E-20	6.05E-18	up	protein_coding
AOC3	2.11257	0.001146756	0.009459241	up	protein_coding
MYO3B	2.1118	6.04E-08	1.54E-06	up	protein_coding
NDUFA4L2	2.10431	0.00316831	0.021639341	up	protein_coding
ITGB8	2.10081	1.98E-07	4.58E-06	up	protein_coding
EYA1	2.09842	3.38E-24	5.20E-22	up	protein_coding
GFAP	2.07735	0.000922883	0.007907643	up	protein_coding
C1R	2.07215	1.43E-80	3.58E-77	up	protein_coding
CH25H	2.06876	0.0089387	0.049765923	up	protein_coding
MRO	2.05387	1.13E-13	6.51E-12	up	protein_coding
PENK	2.05104	8.72E-72	1.46E-68	up	protein_coding
CMKLR1	2.04261	1.23E-15	9.01E-14	up	protein_coding
CYP26B1	2.03485	1.04E-14	6.88E-13	up	protein_coding
VSTM2L	2.02829	5.95E-13	3.25E-11	up	protein_coding
INHBB	2.01436	0.005006173	0.03123155	up	protein_coding
FOS	2.00491	5.82E-06	9.86E-05	up	protein_coding
CHST6	2.00409	1.19E-05	0.000186302	up	protein_coding
LRP1B	1.99324	1.06E-05	0.000168731	up	protein_coding
CYP39A1	1.97722	0.000108913	0.001282512	up	protein_coding
GUCY1A3	1.95709	0.00330512	0.022383827	up	protein_coding
RBM47	1.95423	0.008900725	0.04963814	up	protein_coding
VSTM4	1.9341	1.27E-22	1.63E-20	up	protein_coding
SLC7A11	1.91774	3.00E-16	2.28E-14	up	protein_coding
MFAP4	1.90666	1.33E-25	2.15E-23	up	protein_coding
PIR	1.89695	5.55E-14	3.34E-12	up	protein_coding
FMO3	1.89059	0.003046206	0.020966753	up	protein_coding
PRSS35	1.88306	0.001194293	0.009784262	up	protein_coding
ZBTB16	1.8828	2.91E-26	5.04E-24	up	protein_coding
FRMD4B	1.8787	1.12E-05	0.000176947	up	protein_coding
PODNL1	1.8742	1.91E-08	5.41E-07	up	protein_coding
PLLP	1.86816	0.000720646	0.00638032	up	protein_coding
MAFB	1.85757	6.35E-06	0.000106916	up	protein_coding

SOBP	1.83219	0.008376593	0.047247748	up	protein_coding
RORB	1.82035	8.49E-36	2.24E-33	up	protein_coding
TSC22D3	1.81993	1.33E-49	7.40E-47	up	protein_coding
MEDAG	1.8066	0.005445111	0.033607942	up	protein_coding
MUC1	1.79517	1.16E-05	0.000181804	up	protein_coding
RIPK3	1.7856	0.000238623	0.002515844	up	protein_coding
APOD	1.78051	1.46E-28	2.94E-26	up	protein_coding
NTN1	1.7649	1.94E-07	4.51E-06	up	protein_coding
PLSCR4	1.76355	2.59E-18	2.37E-16	up	protein_coding
ABCC2	1.75023	0.000103442	0.001232462	up	protein_coding
NHSL2	1.74854	1.03E-35	2.66E-33	up	protein_coding
SORBS2	1.7346	1.53E-07	3.61E-06	up	protein_coding
BMF	1.71617	0.008210193	0.046465803	up	protein_coding
RNF144A	1.71448	1.57E-47	7.88E-45	up	protein_coding
NETO1	1.71299	0.000680286	0.00609103	up	protein_coding
IL20RB	1.71242	8.09E-05	0.001002255	up	protein_coding
THRB	1.71182	4.26E-13	2.37E-11	up	protein_coding
SELENOP	1.70614	2.79E-08	7.59E-07	up	protein_coding
PTK2B	1.6991	3.79E-24	5.71E-22	up	protein_coding
ITPRIPL1	1.69589	2.31E-11	1.00E-09	up	protein_coding
PCDH18	1.69364	1.98E-63	2.30E-60	up	protein_coding
ARHGAP28	1.69004	1.55E-16	1.20E-14	up	protein_coding
TRIM16L	1.67085	1.25E-37	3.63E-35	up	protein_coding
RAB27B	1.66019	1.37E-15	9.98E-14	up	protein_coding
SLC6A15	1.64049	1.17E-39	3.75E-37	up	protein_coding
MXRA5	1.63937	2.54E-35	6.48E-33	up	protein_coding
RABL2A	1.63737	5.16E-06	8.87E-05	up	protein_coding
EPHX1	1.62908	2.99E-58	2.65E-55	up	protein_coding
PLEKHH2	1.62144	0.006080424	0.036643131	up	protein_coding
ARHGAP26	1.61493	3.89E-19	3.78E-17	up	protein_coding
FGF7	1.60806	3.52E-16	2.66E-14	up	protein_coding
FAM198B	1.58922	0.000234326	0.002479939	up	protein_coding
FAM84A	1.58127	2.32E-25	3.68E-23	up	protein_coding
GAS1	1.57425	6.50E-76	1.40E-72	up	protein_coding
TMEM51	1.56772	1.34E-07	3.20E-06	up	protein_coding
A4GALT	1.56075	3.02E-26	5.17E-24	up	protein_coding
RASSF2	1.53603	3.97E-08	1.05E-06	up	protein_coding
WNT9A	1.53418	0.000179832	0.001978279	up	protein_coding
HTRA3	1.52902	1.60E-09	5.42E-08	up	protein_coding
EPHB3	1.52025	5.44E-05	0.000711542	up	protein_coding
CYP19A1	1.51818	0.00030147	0.003080147	up	protein_coding
GUCY1B3	1.51789	7.01E-13	3.76E-11	up	protein_coding
LIPC	1.5164	0.007692426	0.044098949	up	protein_coding
CCDC69	1.51539	2.25E-10	8.47E-09	up	protein_coding
FMO4	1.5112	0.004693469	0.029587126	up	protein_coding
IRX5	1.50078	8.81E-05	0.001080747	up	protein_coding
CLCA2	1.50025	1.76E-08	5.04E-07	up	protein_coding
IL1R1	1.50008	1.44E-59	1.44E-56	up	protein_coding
PCSK9	1.4989	0.004244167	0.027152569	up	protein_coding
SLC22A15	1.49603	2.44E-10	9.11E-09	up	protein_coding
APOE	1.49524	0.000759635	0.006674507	up	protein_coding
HMOX1	1.49489	9.31E-51	5.61E-48	up	protein_coding
ADAMTS3	1.49005	2.51E-05	0.000361009	up	protein_coding
STXBP6	1.48696	0.000172187	0.001912193	up	protein_coding
SLC7A8	1.47809	5.83E-26	9.65E-24	up	protein_coding
UNC5D	1.4722	2.19E-06	4.09E-05	up	protein_coding
AKR1C1	1.4721	7.56E-29	1.56E-26	up	protein_coding
RIPOR3	1.4668	2.41E-20	2.69E-18	up	protein_coding
PDE4D	1.46629	2.47E-27	4.53E-25	up	protein_coding
NNMT	1.46034	2.88E-38	9.05E-36	up	protein_coding
CEBPD	1.45771	1.08E-41	3.97E-39	up	protein_coding
IL1RN	1.45742	0.000707882	0.00630438	up	protein_coding
NR4A2	1.45579	2.84E-06	5.20E-05	up	protein_coding
ITGA11	1.44321	4.06E-06	7.19E-05	up	protein_coding
ADAMTS9	1.4428	5.03E-07	1.07E-05	up	protein_coding
AFF2	1.44039	0.000372941	0.003692634	up	protein_coding

IGFBP7	1.43582	5.25E-35	1.32E-32	up	protein_coding
SLC12A8	1.43092	1.02E-22	1.34E-20	up	protein_coding
FKBP5	1.42884	1.02E-52	6.68E-50	up	protein_coding
C5AR2	1.42801	3.57E-13	2.00E-11	up	protein_coding
WDR31	1.42435	2.31E-06	4.29E-05	up	protein_coding
SLC1A4	1.42308	0.00132324	0.010628264	up	protein_coding
C1S	1.423	6.82E-49	3.67E-46	up	protein_coding
STOM	1.41532	2.41E-51	1.51E-48	up	protein_coding
KIAA1522	1.41255	7.97E-14	4.69E-12	up	protein_coding
SLC25A27	1.41	0.007322077	0.042460716	up	protein_coding
CRISPLD2	1.40903	4.54E-24	6.70E-22	up	protein_coding
HRCT1	1.40694	0.002946307	0.020397779	up	protein_coding
WISP2	1.39917	1.16E-06	2.29E-05	up	protein_coding
C1QTNF6	1.39886	7.34E-15	4.89E-13	up	protein_coding
METTL7A	1.39029	5.70E-34	1.34E-31	up	protein_coding
MASP1	1.38395	7.95E-20	8.32E-18	up	protein_coding
AKAP6	1.38255	0.002196441	0.016034127	up	protein_coding
MSC	1.37784	8.61E-20	8.94E-18	up	protein_coding
SLC46A3	1.37686	8.56E-07	1.74E-05	up	protein_coding
OLFML2B	1.3735	5.23E-24	7.64E-22	up	protein_coding
SLC6A9	1.36704	5.68E-07	1.20E-05	up	protein_coding
PIM1	1.36617	1.48E-15	1.06E-13	up	protein_coding
BOC	1.36359	9.28E-08	2.25E-06	up	protein_coding
KYNU	1.3625	1.08E-26	1.91E-24	up	protein_coding
PID1	1.36119	1.79E-13	1.03E-11	up	protein_coding
VCAN	1.35275	1.32E-34	3.26E-32	up	protein_coding
ENPP2	1.34793	5.14E-41	1.80E-38	up	protein_coding
SLC14A1	1.34775	6.00E-42	2.26E-39	up	protein_coding
OCA2	1.34544	0.00859274	0.048241364	up	protein_coding
SERTAD4	1.34519	4.23E-05	0.0005714	up	protein_coding
SOCS1	1.34397	0.002559347	0.018215392	up	protein_coding
COL1A1	1.33725	1.99E-44	8.84E-42	up	protein_coding
DIRAS3	1.32558	0.004703878	0.029636154	up	protein_coding
IFITM1	1.32534	0.000248668	0.002602464	up	protein_coding
TIMP4	1.32339	5.90E-14	3.54E-12	up	protein_coding
SLC9A9	1.31961	6.21E-17	4.98E-15	up	protein_coding
MBP	1.31737	0.000107145	0.001267554	up	protein_coding
ACSL5	1.30737	0.005955473	0.036090134	up	protein_coding
IGFBP5	1.30462	1.49E-05	0.000224721	up	protein_coding
CORIN	1.3035	6.07E-05	0.000782361	up	protein_coding
PDE3B	1.29146	0.000101072	0.001208045	up	protein_coding
PCDH7	1.2888	9.08E-08	2.21E-06	up	protein_coding
C1orf115	1.28079	0.002121485	0.015575527	up	protein_coding
ST6GAL1	1.27957	5.66E-10	2.04E-08	up	protein_coding
PDGFRB	1.27847	7.64E-30	1.64E-27	up	protein_coding
FGF14	1.27133	0.000385199	0.003799019	up	protein_coding
CLMN	1.27091	0.000749201	0.006602085	up	protein_coding
HECTD2	1.25974	7.11E-05	0.000899495	up	protein_coding
ACACB	1.25665	1.73E-06	3.31E-05	up	protein_coding
COL4A2	1.24491	1.10E-27	2.07E-25	up	protein_coding
ZNF493	1.24174	0.004974226	0.03107086	up	protein_coding
EVI2A	1.2351	0.000632178	0.005745687	up	protein_coding
CYP27A1	1.23338	1.80E-14	1.16E-12	up	protein_coding
MYO1D	1.23238	4.11E-09	1.29E-07	up	protein_coding
SEC31B	1.23193	0.002013607	0.014938384	up	protein_coding
DHRS12	1.2303	0.00457196	0.028954466	up	protein_coding
SLC40A1	1.23013	0.00018886	0.002055084	up	protein_coding
FOXO1	1.22921	6.10E-05	0.000786352	up	protein_coding
LAMB3	1.2292	2.35E-07	5.31E-06	up	protein_coding
CMPK2	1.22823	0.003561059	0.023740396	up	protein_coding
ENPP1	1.2244	8.76E-18	7.71E-16	up	protein_coding
PLA2R1	1.22044	3.91E-06	6.94E-05	up	protein_coding
IMPA2	1.22043	0.001516716	0.011896741	up	protein_coding
PCDH10	1.21926	6.73E-07	1.39E-05	up	protein_coding
EMILIN2	1.21896	4.01E-10	1.47E-08	up	protein_coding
PLCD4	1.21817	2.24E-10	8.46E-09	up	protein_coding

FAM129A	1.21343	9.80E-22	1.16E-19	up	protein_coding
PTGFR	1.21204	8.84E-18	7.74E-16	up	protein_coding
KSR1	1.21203	4.09E-08	1.08E-06	up	protein_coding
TMEM100	1.20836	0.000710853	0.006319624	up	protein_coding
PAPLN	1.20784	2.77E-06	5.08E-05	up	protein_coding
ITPKB	1.20443	7.22E-06	0.00012036	up	protein_coding
JAG1	1.20011	1.14E-20	1.29E-18	up	protein_coding
TCEA3	1.19406	0.000171726	0.001908635	up	protein_coding
ANGPTL2	1.19292	2.30E-16	1.77E-14	up	protein_coding
PPM1H	1.18723	1.63E-09	5.49E-08	up	protein_coding
EYA2	1.18008	1.33E-18	1.25E-16	up	protein_coding
SHTN1	1.17973	0.000641189	0.005813547	up	protein_coding
MFSD7	1.17856	0.004769172	0.029951515	up	protein_coding
ADAMTS2	1.17231	3.95E-33	9.02E-31	up	protein_coding
EFEMP1	1.16936	1.21E-08	3.55E-07	up	protein_coding
ALDH3A2	1.16767	8.97E-18	7.76E-16	up	protein_coding
VWA5A	1.16761	1.30E-16	1.02E-14	up	protein_coding
DTX4	1.16142	1.83E-05	0.000270198	up	protein_coding
CMTM8	1.16046	1.33E-05	0.000203999	up	protein_coding
EEPD1	1.15951	7.31E-05	0.000919308	up	protein_coding
IFIT1	1.15654	0.002559269	0.018215392	up	protein_coding
PIK3C2B	1.15102	4.44E-08	1.17E-06	up	protein_coding
TGFB3	1.1462	0.001693604	0.013073128	up	protein_coding
LRRC75A	1.14611	0.003100069	0.021260994	up	protein_coding
SLC22A23	1.14451	1.15E-05	0.00018154	up	protein_coding
PAN2	1.14296	2.39E-12	1.19E-10	up	protein_coding
C1RL	1.12956	3.32E-14	2.08E-12	up	protein_coding
PPP1R3E	1.12892	0.000579554	0.005341546	up	protein_coding
STAT5A	1.12623	1.61E-12	8.21E-11	up	protein_coding
DPYD	1.12538	2.02E-14	1.29E-12	up	protein_coding
CORO6	1.12274	2.51E-07	5.64E-06	up	protein_coding
MYLIP	1.12223	2.24E-07	5.07E-06	up	protein_coding
TSPAN9	1.12157	5.61E-16	4.14E-14	up	protein_coding
THBS2	1.11873	1.80E-25	2.88E-23	up	protein_coding
ADAMTS15	1.11644	5.66E-12	2.65E-10	up	protein_coding
PER3	1.11417	3.52E-05	0.000486947	up	protein_coding
COL6A3	1.11226	1.90E-34	4.62E-32	up	protein_coding
LYRM9	1.11033	0.000242465	0.002549891	up	protein_coding
SCART1	1.10853	0.006644085	0.039285402	up	protein_coding
ABCC3	1.09874	3.66E-12	1.78E-10	up	protein_coding
HRH1	1.09766	8.11E-08	2.02E-06	up	protein_coding
USP13	1.0951	3.23E-28	6.39E-26	up	protein_coding
PTPRU	1.09464	2.51E-09	8.08E-08	up	protein_coding
EDNRA	1.0942	0.000756537	0.006655049	up	protein_coding
SAMHD1	1.09385	4.95E-23	6.71E-21	up	protein_coding
STRADA	1.09247	0.006465157	0.038438712	up	protein_coding
SDC2	1.09165	8.07E-17	6.43E-15	up	protein_coding
LRRN4CL	1.08761	6.02E-08	1.53E-06	up	protein_coding
APOL1	1.08539	8.77E-08	2.15E-06	up	protein_coding
DENND6B	1.08028	0.000710018	0.006319624	up	protein_coding
FAM43A	1.07104	6.01E-13	3.27E-11	up	protein_coding
FLRT3	1.06871	1.77E-07	4.15E-06	up	protein_coding
COL4A1	1.06798	2.95E-20	3.24E-18	up	protein_coding
TMC7	1.06449	0.00293716	0.020346655	up	protein_coding
PLEKHF1	1.05994	2.20E-08	6.18E-07	up	protein_coding
WNT2B	1.05796	2.90E-17	2.43E-15	up	protein_coding
PAPPA	1.05779	1.30E-25	2.12E-23	up	protein_coding
PIP	1.05639	7.73E-11	3.15E-09	up	protein_coding
C16orf86	1.05614	0.004907597	0.030731146	up	protein_coding
FZD5	1.05528	0.000621653	0.005673997	up	protein_coding
ISM1	1.05521	3.49E-05	0.000482784	up	protein_coding
TENM3	1.04488	5.25E-09	1.63E-07	up	protein_coding
ARSG	1.0441	6.13E-07	1.28E-05	up	protein_coding
PCBP3	1.04393	2.73E-14	1.72E-12	up	protein_coding
STON1	1.04337	9.95E-10	3.47E-08	up	protein_coding
PXK	1.04112	3.29E-20	3.59E-18	up	protein_coding

SPTLC3	1.04095	2.17E-07	4.96E-06	up	protein_coding
COL1A2	1.03687	5.29E-27	9.48E-25	up	protein_coding
COL12A1	1.03557	1.34E-24	2.10E-22	up	protein_coding
TBX4	1.03437	0.008951356	0.049817966	up	protein_coding
TRIM45	1.02801	0.003809765	0.024988128	up	protein_coding
RETREG1	1.02796	0.004595964	0.029082026	up	protein_coding
SPRY1	1.02788	1.69E-12	8.56E-11	up	protein_coding
PCDHGA10	1.02669	2.13E-11	9.29E-10	up	protein_coding
MN1	1.02527	3.26E-12	1.61E-10	up	protein_coding
LRP1	1.02514	1.14E-23	1.60E-21	up	protein_coding
LAMA1	1.02393	1.37E-20	1.55E-18	up	protein_coding
PELI2	1.02301	5.51E-11	2.29E-09	up	protein_coding
COL27A1	1.02166	6.24E-19	5.87E-17	up	protein_coding
ST3GAL5	1.02083	1.63E-10	6.29E-09	up	protein_coding
GLIS2	1.02079	4.14E-13	2.31E-11	up	protein_coding
GPC6	1.02074	1.11E-09	3.84E-08	up	protein_coding
COL4A5	1.02047	1.27E-09	4.37E-08	up	protein_coding
RRAD	1.0181	2.72E-08	7.41E-07	up	protein_coding
THEMIS2	1.01795	1.08E-08	3.20E-07	up	protein_coding
NCAM2	1.01757	0.00038266	0.00377645	up	protein_coding
KIAA1671	1.01731	4.53E-05	0.000607987	up	protein_coding
COL4A6	1.01275	3.68E-05	0.000504734	up	protein_coding
MMD	1.01231	8.96E-18	7.76E-16	up	protein_coding
LRP5L	1.01004	0.00517527	0.032153285	up	protein_coding
SLC39A8	1.00703	7.15E-14	4.27E-12	up	protein_coding
SLC48A1	1.00518	5.95E-10	2.14E-08	up	protein_coding
DCN	1.00267	5.66E-24	8.20E-22	up	protein_coding
STON2	1.00186	0.000157882	0.001769776	up	protein_coding
SLC37A2	-1.0018	0.003445233	0.023120798	down	protein_coding
HIST1H2AI	-1.0037	0.003606514	0.023948017	down	protein_coding
UGCG	-1.0055	9.96E-21	1.14E-18	down	protein_coding
CRIM1	-1.0068	4.14E-24	6.17E-22	down	protein_coding
EGLN1	-1.0081	3.01E-15	2.08E-13	down	protein_coding
ATRNL1	-1.0122	8.06E-05	0.000999841	down	protein_coding
FAM162A	-1.0123	1.56E-07	3.67E-06	down	protein_coding
HIST1H2BO	-1.0135	1.55E-05	0.00023245	down	protein_coding
FAM107B	-1.0137	1.81E-14	1.16E-12	down	protein_coding
AKAP12	-1.0149	2.14E-09	6.98E-08	down	protein_coding
PRAG1	-1.0151	0.00024742	0.002592033	down	protein_coding
FILIP1L	-1.0177	0.000276709	0.002854271	down	protein_coding
GADD45A	-1.0198	3.39E-12	1.67E-10	down	protein_coding
KPNA6	-1.0198	2.02E-26	3.54E-24	down	protein_coding
HCAR1	-1.0224	0.000312092	0.003171461	down	protein_coding
PEAR1	-1.0249	6.36E-05	0.000815081	down	protein_coding
SEC61A2	-1.0251	0.00043301	0.004180214	down	protein_coding
CDCA5	-1.0274	1.56E-06	3.03E-05	down	protein_coding
RELT	-1.0278	0.001541585	0.012041635	down	protein_coding
POU2F2	-1.0286	5.05E-14	3.07E-12	down	protein_coding
SH2D5	-1.0298	0.003284674	0.022302614	down	protein_coding
CDC6	-1.0324	0.000558118	0.005188427	down	protein_coding
SMTN	-1.034	2.96E-24	4.60E-22	down	protein_coding
BIRC3	-1.0357	8.93E-06	0.000145575	down	protein_coding
TMEM160	-1.0374	0.001978254	0.01470509	down	protein_coding
MYPN	-1.0384	6.77E-15	4.55E-13	down	protein_coding
FOXD1	-1.0399	1.32E-16	1.02E-14	down	protein_coding
CDC25A	-1.05	2.30E-05	0.000334045	down	protein_coding
NFKBIZ	-1.0506	1.08E-14	7.10E-13	down	protein_coding
CDCA7	-1.0525	0.000181729	0.001993333	down	protein_coding
HIST1H2BH	-1.0544	0.000199213	0.002150648	down	protein_coding
EPOP	-1.0572	0.000672312	0.00603039	down	protein_coding
PM20D2	-1.0632	2.17E-06	4.06E-05	down	protein_coding
PLD5	-1.0632	0.008189144	0.046381539	down	protein_coding
C6orf132	-1.066	1.61E-08	4.63E-07	down	protein_coding
SLC43A2	-1.0667	1.76E-06	3.35E-05	down	protein_coding
SPRY2	-1.0672	3.76E-24	5.71E-22	down	protein_coding
SPHK1	-1.0735	6.88E-07	1.42E-05	down	protein_coding

CARD10	-1.0736	5.30E-05	0.000696398	down	protein_coding
CCND1	-1.0742	9.68E-41	3.31E-38	down	protein_coding
APAF1	-1.0763	3.82E-11	1.62E-09	down	protein_coding
PPP1R13B	-1.0804	0.007093222	0.041436741	down	protein_coding
SHCBP1	-1.0852	2.59E-06	4.77E-05	down	protein_coding
HIST1H3H	-1.0864	0.000117548	0.001367012	down	protein_coding
NF2	-1.0893	1.26E-29	2.68E-27	down	protein_coding
TNFSF4	-1.0899	0.003106223	0.021282856	down	protein_coding
HMMR	-1.0902	0.001913366	0.014335968	down	protein_coding
UAP1	-1.0907	4.18E-17	3.40E-15	down	protein_coding
KIAA1549L	-1.0908	1.82E-14	1.17E-12	down	protein_coding
HIST1H1B	-1.0944	4.86E-10	1.76E-08	down	protein_coding
HIST1H3G	-1.102	2.13E-07	4.89E-06	down	protein_coding
RASA2	-1.1037	1.41E-10	5.47E-09	down	protein_coding
RNF122	-1.1039	0.00598738	0.036256511	down	protein_coding
ARHGAP39	-1.1123	0.00020343	0.002193029	down	protein_coding
HIST1H2AB	-1.1132	0.003748759	0.024653412	down	protein_coding
SEMA6D	-1.1136	0.000188704	0.002055084	down	protein_coding
DPYSL4	-1.1163	2.06E-07	4.74E-06	down	protein_coding
CNNM1	-1.1217	0.007648922	0.043922441	down	protein_coding
ORC6	-1.1237	0.001269205	0.01028753	down	protein_coding
INPP4B	-1.1321	4.05E-07	8.77E-06	down	protein_coding
DUSP6	-1.1331	2.54E-34	6.08E-32	down	protein_coding
MOK	-1.1397	8.17E-13	4.30E-11	down	protein_coding
FOXG1	-1.1438	0.008898737	0.04963814	down	protein_coding
MAD2L1	-1.1486	6.18E-08	1.57E-06	down	protein_coding
IKZF2	-1.1578	3.36E-06	6.06E-05	down	protein_coding
PFKFB4	-1.1618	2.55E-19	2.52E-17	down	protein_coding
ABAT	-1.1651	8.38E-08	2.07E-06	down	protein_coding
PLCB4	-1.171	9.94E-15	6.59E-13	down	protein_coding
EXO1	-1.1781	0.00270912	0.019118723	down	protein_coding
ZNF185	-1.1788	2.99E-13	1.70E-11	down	protein_coding
ADRB2	-1.1853	1.88E-08	5.35E-07	down	protein_coding
VLDLR	-1.1882	3.65E-08	9.72E-07	down	protein_coding
WNK3	-1.1899	0.001738005	0.013313508	down	protein_coding
EHF	-1.193	0.008384254	0.047273254	down	protein_coding
GYS1	-1.2032	1.43E-29	2.98E-27	down	protein_coding
HSPE1	-1.2075	1.55E-05	0.000232216	down	protein_coding
RGMB	-1.2167	2.74E-54	1.96E-51	down	protein_coding
TICRR	-1.2169	9.68E-05	0.001163035	down	protein_coding
KCTD12	-1.2197	1.17E-32	2.63E-30	down	protein_coding
RBM38	-1.224	1.44E-06	2.80E-05	down	protein_coding
VEGFC	-1.2279	2.62E-20	2.90E-18	down	protein_coding
TES	-1.2404	1.45E-06	2.83E-05	down	protein_coding
MYBL1	-1.2492	0.000104321	0.00124195	down	protein_coding
IL7R	-1.2529	2.44E-09	7.90E-08	down	protein_coding
HCLS1	-1.2658	0.000149054	0.001683987	down	protein_coding
MPP2	-1.2663	9.72E-07	1.95E-05	down	protein_coding
HIST1H2BM	-1.2685	0.001647033	0.012765988	down	protein_coding
ACPP	-1.269	7.66E-06	0.000126701	down	protein_coding
PSG5	-1.2692	3.49E-08	9.36E-07	down	protein_coding
KCNJ2	-1.2703	3.61E-08	9.61E-07	down	protein_coding
AMPD3	-1.2737	1.67E-23	2.31E-21	down	protein_coding
CXCL1	-1.2865	0.000465932	0.004438288	down	protein_coding
NES	-1.2899	5.51E-26	9.21E-24	down	protein_coding
NDN	-1.2931	0.000125804	0.001454036	down	protein_coding
ZC3HAV1L	-1.2954	5.76E-07	1.21E-05	down	protein_coding
DOK5	-1.3129	1.70E-09	5.70E-08	down	protein_coding
MFSD2B	-1.3145	0.000930257	0.007960036	down	protein_coding
SLC4A7	-1.3151	1.83E-23	2.50E-21	down	protein_coding
IL12A	-1.3203	0.000992867	0.008372104	down	protein_coding
KCTD4	-1.3204	1.99E-05	0.000292903	down	protein_coding
ADGRG1	-1.3232	1.30E-05	0.000200679	down	protein_coding
ANKRD37	-1.3339	0.002293147	0.016635255	down	protein_coding
EDN1	-1.3379	0.001616008	0.012556129	down	protein_coding
NRG1	-1.3402	6.47E-24	9.28E-22	down	protein_coding

HIST1H3J	-1.347	6.27E-05	0.000804908	down	protein_coding
MT2A	-1.3472	2.09E-43	8.51E-41	down	protein_coding
HIST1H4L	-1.3492	0.002405849	0.017311074	down	protein_coding
NPDC1	-1.351	1.15E-11	5.20E-10	down	protein_coding
BTBD11	-1.3536	0.001854169	0.013975867	down	protein_coding
SLC4A4	-1.3628	4.40E-12	2.11E-10	down	protein_coding
WNT16	-1.3737	1.54E-12	7.85E-11	down	protein_coding
CLDN1	-1.3903	0.006378333	0.03802759	down	protein_coding
MYEOV	-1.399	2.97E-05	0.000419721	down	protein_coding
PDK1	-1.4167	1.81E-22	2.28E-20	down	protein_coding
CORO1A	-1.4171	0.007244619	0.04212508	down	protein_coding
ARHGAP29	-1.4216	5.46E-20	5.87E-18	down	protein_coding
BNIP3	-1.4226	8.86E-48	4.60E-45	down	protein_coding
PPP1R3C	-1.4254	6.66E-45	3.04E-42	down	protein_coding
P3H2	-1.4543	0.001275179	0.010313745	down	protein_coding
AK4	-1.4661	1.32E-36	3.60E-34	down	protein_coding
PRKCZ	-1.4779	0.000165685	0.001851053	down	protein_coding
PDCD1LG2	-1.484	2.17E-18	1.99E-16	down	protein_coding
DDIAS	-1.4894	0.000536221	0.005018946	down	protein_coding
FGF5	-1.4936	4.32E-44	1.81E-41	down	protein_coding
PSD	-1.4959	0.00746325	0.043113366	down	protein_coding
GINS2	-1.5049	3.06E-06	5.58E-05	down	protein_coding
NRK	-1.5051	8.50E-05	0.001047567	down	protein_coding
RP1	-1.5058	0.000144756	0.00164158	down	protein_coding
SLC2A1	-1.5068	6.10E-34	1.41E-31	down	protein_coding
MMP1	-1.5075	2.73E-58	2.57E-55	down	protein_coding
HIST2H3D	-1.5085	0.000773004	0.00678406	down	protein_coding
HBEGF	-1.5163	2.77E-08	7.54E-07	down	protein_coding
EEF1A2	-1.5417	0.000257287	0.002677779	down	protein_coding
IL13RA2	-1.5473	1.68E-05	0.000249288	down	protein_coding
ANO7	-1.5609	0.000505311	0.004768158	down	protein_coding
GNAZ	-1.5611	3.10E-05	0.000435377	down	protein_coding
SMURF2	-1.5676	1.25E-54	9.88E-52	down	protein_coding
DCBLD2	-1.5722	9.90E-53	6.68E-50	down	protein_coding
ADAMTS1	-1.5843	3.36E-47	1.63E-44	down	protein_coding
BHLHE41	-1.6419	0.003977622	0.025853685	down	protein_coding
KCTD16	-1.6423	0.002687733	0.018994489	down	protein_coding
ANGPTL4	-1.6484	3.65E-44	1.57E-41	down	protein_coding
NEK10	-1.6689	4.89E-08	1.27E-06	down	protein_coding
CENPU	-1.6967	0.002269817	0.016513743	down	protein_coding
MARCKSL1	-1.7509	7.03E-24	9.99E-22	down	protein_coding
PLAT	-1.7525	1.01E-39	3.32E-37	down	protein_coding
STC1	-1.7751	2.15E-62	2.31E-59	down	protein_coding
AC106886.5	-1.7818	0.003472703	0.023213007	down	protein_coding
MCM10	-1.7886	1.35E-05	0.000205985	down	protein_coding
GPRC5A	-1.8048	2.23E-08	6.23E-07	down	protein_coding
PTPRN	-1.8161	2.70E-11	1.16E-09	down	protein_coding
ENC1	-1.8894	1.56E-41	5.60E-39	down	protein_coding
DHRS3	-1.8945	1.25E-12	6.42E-11	down	protein_coding
KIT	-1.9252	4.46E-13	2.46E-11	down	protein_coding
SYNPO2L	-1.9323	0.000208077	0.002236716	down	protein_coding
CCND2	-1.94	1.88E-74	3.54E-71	down	protein_coding
CXCL3	-1.9527	1.34E-19	1.39E-17	down	protein_coding
KRTAP1-1	-1.9765	2.45E-09	7.91E-08	down	protein_coding
LIF	-2.0172	2.54E-54	1.92E-51	down	protein_coding
NPTX1	-2.0482	2.44E-68	3.67E-65	down	protein_coding
KRT19	-2.0815	2.25E-05	0.000327268	down	protein_coding
RAB20	-2.1555	5.23E-05	0.000687895	down	protein_coding
PRKG2	-2.1625	6.34E-11	2.62E-09	down	protein_coding
KRT34	-2.1764	1.00E-28	2.04E-26	down	protein_coding
SHISA2	-2.2001	7.45E-07	1.53E-05	down	protein_coding
CORO2B	-2.2039	0.004838494	0.030348904	down	protein_coding
CXCL8	-2.2203	1.24E-65	1.69E-62	down	protein_coding
PPFIA4	-2.2936	5.74E-08	1.47E-06	down	protein_coding
CEND1	-2.428	4.55E-08	1.19E-06	down	protein_coding
IL11	-2.471	9.23E-05	0.001121944	down	protein_coding

RAB39B	-2.5786	0.002502387	0.017903063	down	protein_coding
DOK3	-2.6646	0.001298048	0.01045939	down	protein_coding
SERPINB2	-2.6696	3.48E-11	1.48E-09	down	protein_coding
KRT15	-2.8118	0.000728404	0.006437656	down	protein_coding
CXCL5	-2.8297	2.68E-31	5.93E-29	down	protein_coding
GRIA1	-2.8653	0.001742347	0.013339985	down	protein_coding
PSG1	-2.8888	0.003351627	0.022584119	down	protein_coding
MGP	-2.9122	0.007214037	0.041979672	down	protein_coding
KRTAP1-5	-2.9365	7.99E-161	4.01E-157	down	protein_coding
OLAH	-2.9776	4.21E-05	0.000569073	down	protein_coding
GPR146	-3.0398	0.000129807	0.001498005	down	protein_coding
KRTAP2-3	-3.3272	7.19E-13	3.83E-11	down	protein_coding
FBXL16	-3.5056	6.45E-05	0.000822021	down	protein_coding
TTN	-3.5713	5.98E-05	0.000772254	down	protein_coding
KPRP	-4.0641	3.64E-14	2.25E-12	down	protein_coding
