# Supplementary material for: lncRNA HHIP-AS1 Promotes the Osteogenic Differentiation Potential and Inhibits the Migration Ability of Periodontal Ligament Stem Cells
Source: Stem Cells Int. 2021 Apr 27;2021:5595580. doi: 10.1155/2021/5595580 (PMC8554619; doi:10.1155/2021/5595580)
Supplement: Supplementary 3 — Table S3: the differentially expressed lncRNAs in HHIP-AS1-depleted PDLSCs. [file 5595580.f3.pdf]

Supplementary Table 3. The differentially expressed lncRNAs in HHIP-AS1-depleted PDLSCs.

external_gene_name	log2FC	P value	FDR	Style	gene_biotype
AC099805.1	3.524706858	0.000311135	0.003163867	up	lincRNA
LINC01126	2.663642979	0.001893272	0.0142137	up	lincRNA
FP671120.4	2.63935472	0.001712496	0.013176617	up	lincRNA
AC104051.2	2.369860908	1.73E-05	0.000256927	up	lincRNA
C1orf132	1.876658149	2.44E-17	2.05E-15	up	lincRNA
AC013644.1	1.870248242	0.004034475	0.026155485	up	lincRNA
NEAT1	1.751400999	2.15E-10	8.14E-09	up	lincRNA
PSMB8-AS1	1.745219528	0.002078267	0.015327471	up	lincRNA
AL603840.1	1.686222629	7.91E-07	1.61E-05	up	lincRNA
AC048341.2	1.555777196	0.000268557	0.002777799	up	lincRNA
CARMN	1.487598386	6.25E-15	4.24E-13	up	lincRNA
AC099786.3	1.402876801	0.007630632	0.043878319	up	lincRNA
LINC00511	1.168329709	0.000450711	0.004328898	up	lincRNA
AC144831.1	1.138952786	0.001787412	0.013595167	up	lincRNA
AC009549.1	-1.553219302	1.22E-07	2.94E-06	down	lincRNA
AC091182.2	-1.675078174	0.008117507	0.046045069	down	lincRNA
LINC02407	-1.761160936	0.001246496	0.010130719	down	lincRNA
AL139220.2	-1.763517935	2.00E-05	0.000293656	down	lincRNA
MIR210HG	-2.535568353	1.66E-19	1.69E-17	down	lincRNA
LINC00973	-3.337491217	1.33E-08	3.87E-07	down	lincRNA
