# Supplementary figures and images for: lncRNA HHIP-AS1 Promotes the Osteogenic Differentiation Potential and Inhibits the Migration Ability of Periodontal Ligament Stem Cells
Source: Stem Cells Int. 2021 Apr 27;2021:5595580. doi: 10.1155/2021/5595580 (PMC8554619; doi:10.1155/2021/5595580)

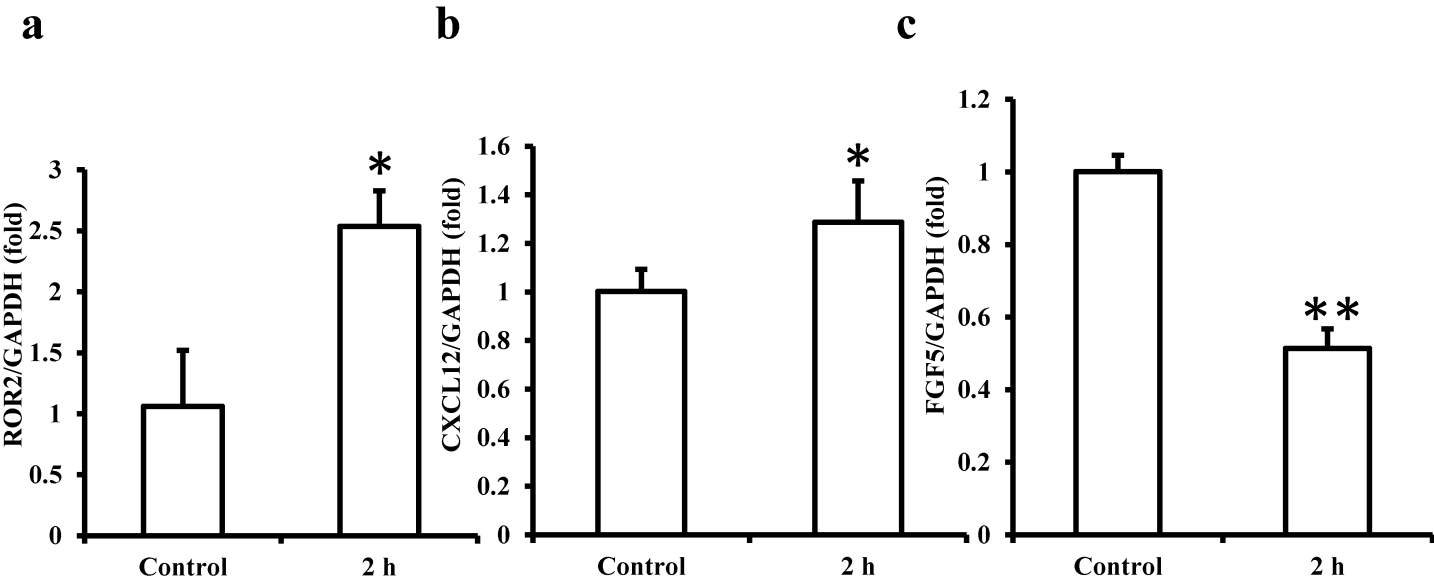

Supplement: Supplementary 4 — Figure S1: ROR2 and CXCL12 were upregulated while FGF5 was downregulated under compressive pressure. Real-time RT-PCR results showed expressions of ROR2 (a), CXCL12 (b), and FGF5 (c) at 2 h under continuous compressive pressure. GAPDH was used as internal control. Student's t-test was performed to determine statistical significance. All error bars represent the SD (n = 3). ∗P ≤ 0.05; ∗∗P ≤ 0.01. [file 5595580.f4.pdf]

**a**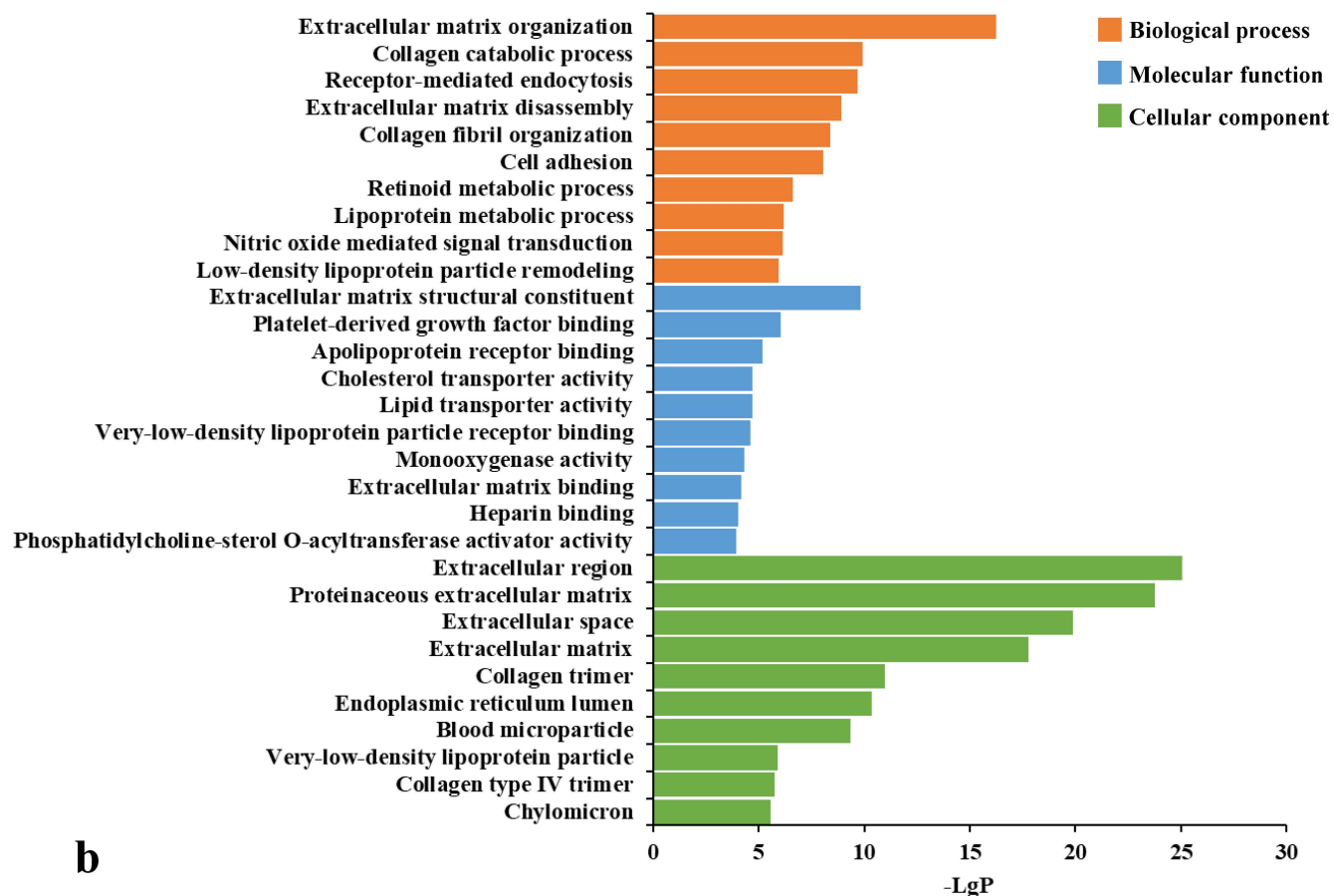**b**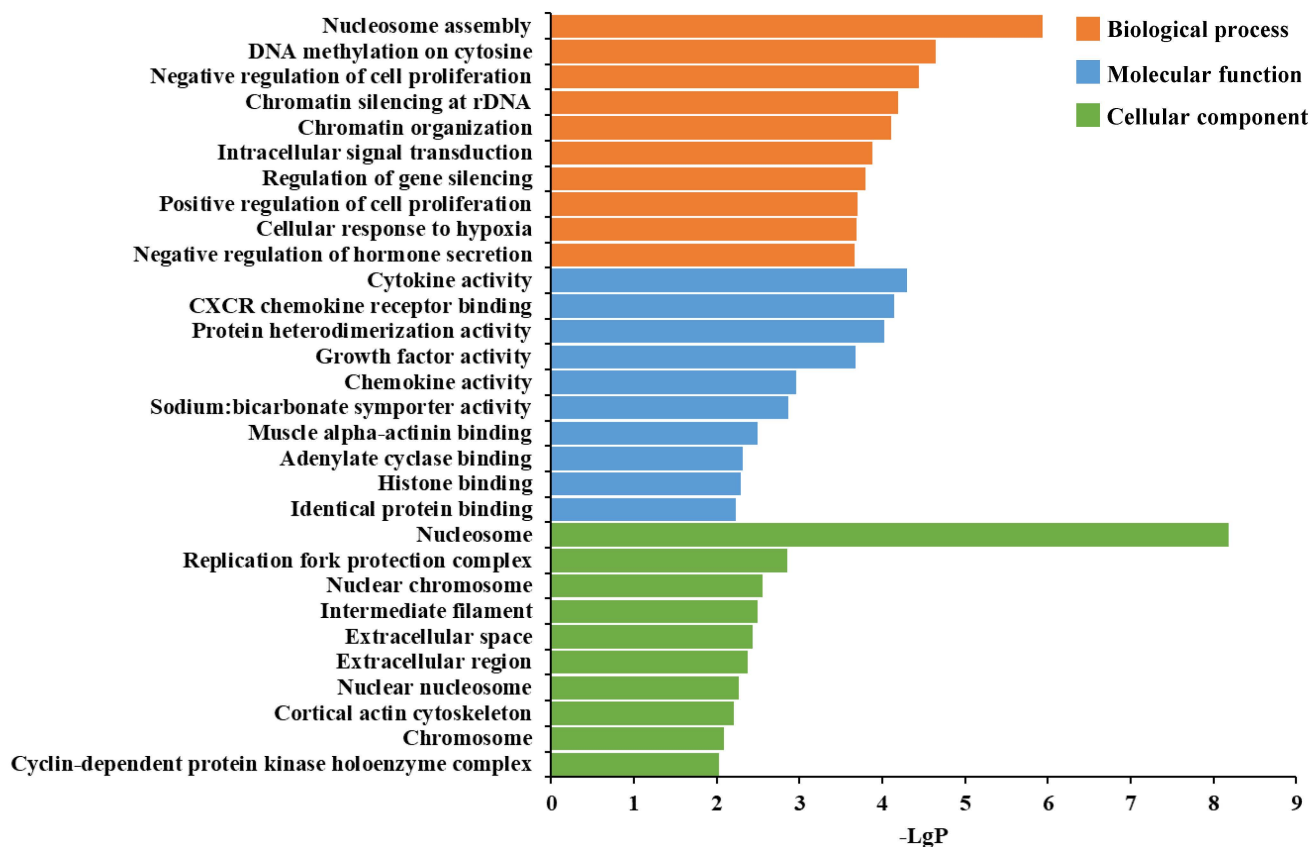

Supplement: Supplementary 5 — Figure S2: significant GO analysis of differentially expressed genes from aspects of biological process, molecular function, and cellular component. (a) Upregulated GO terms. (b) Downregulated GO terms. [file 5595580.f5.pdf]

**a**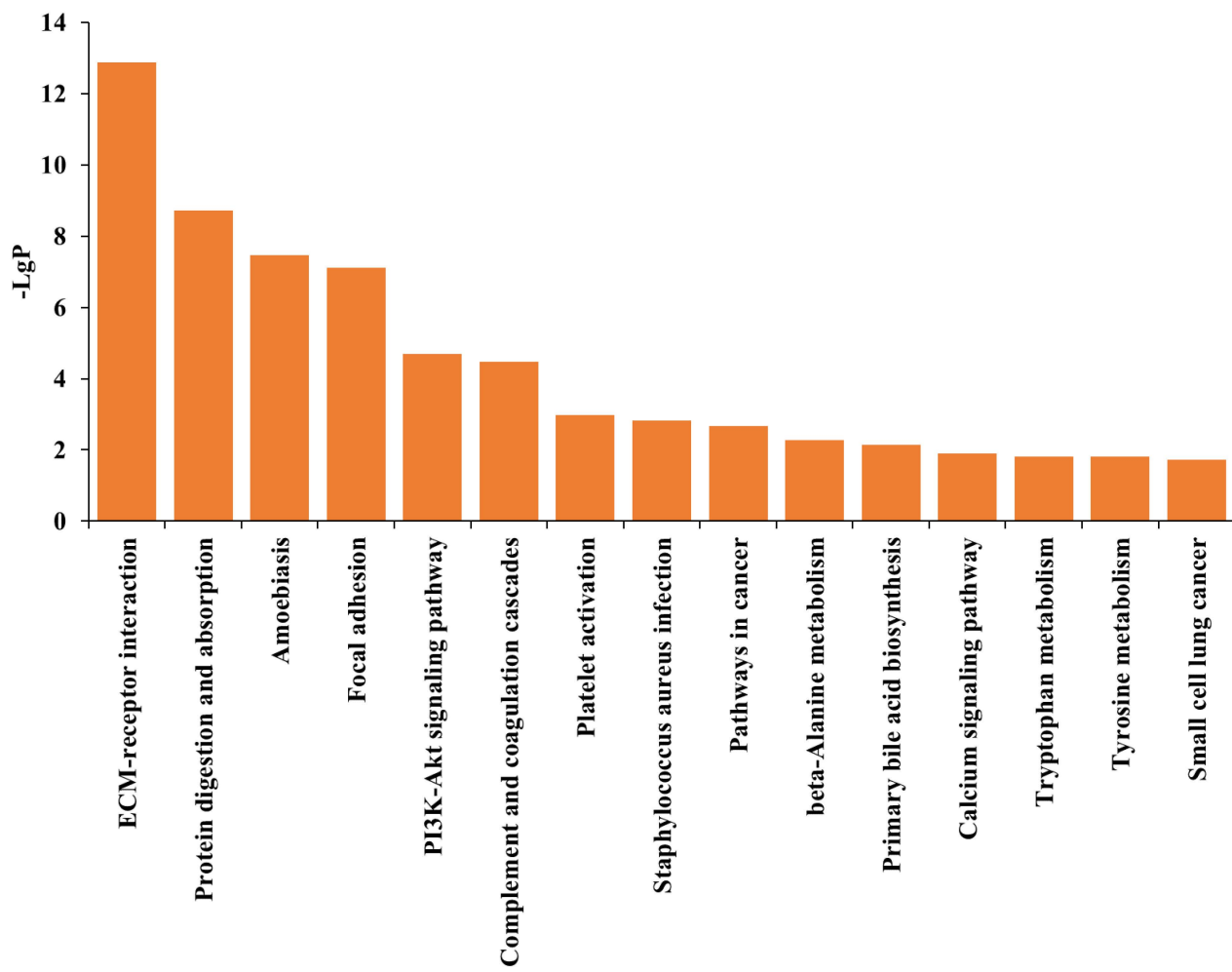**b**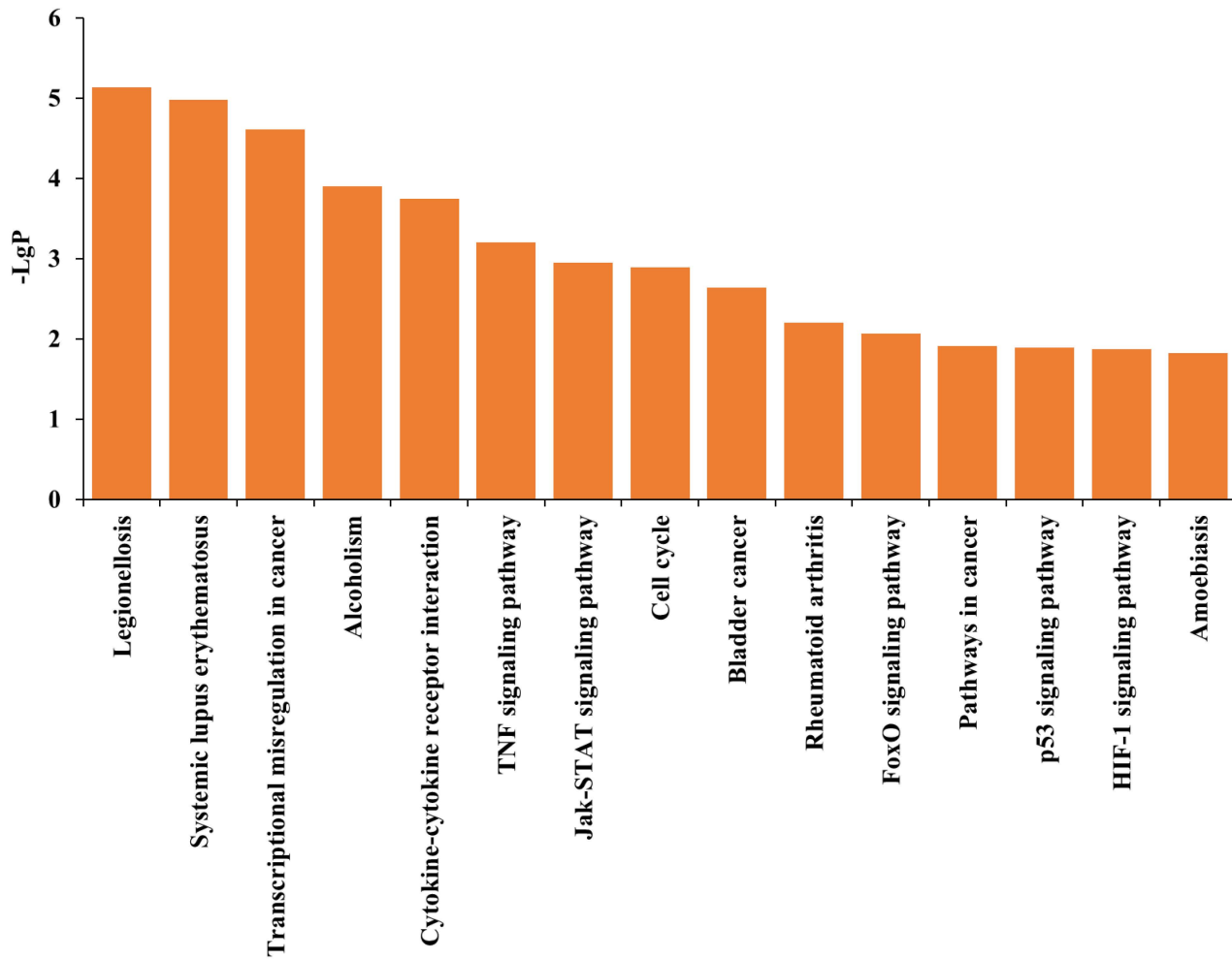

Supplement: Supplementary 8 — Figure S3: significant pathway analysis of differentially expressed genes in HHIP-AS1-depleted PDLSCs. (a) Upregulated pathways. (b) Downregulated pathways. [file 5595580.f8.pdf]
