# Supplementary material for: lncRNA HHIP-AS1 Promotes the Osteogenic Differentiation Potential and Inhibits the Migration Ability of Periodontal Ligament Stem Cells
Source: Stem Cells Int. 2021 Apr 27;2021:5595580. doi: 10.1155/2021/5595580 (PMC8554619; doi:10.1155/2021/5595580)
Supplement: Supplementary 6 — Table S4: the significant upregulated and downregulated GO functions in HHIP-AS1-depleted PDLSCs. [file 5595580.f6.pdf]

Supplementary Table 4. The significant upregulated and downregulated GO functions in HHIP-AS1-depleted PDLSCs.

GOID	GOTerm	DifGene	AllDifGene	GeneInGO	AllGene	P-Value	FDR	Enrichment	(-log10P)	Style
GO:0030198	extracellular matrix organization	35	323	317	16775	5.52E-17	1.09E-13	5.734146556	16.25811	up
GO:0030574	collagen catabolic process	14	323	74	16775	1.17E-10	1.15E-07	9.825537612	9.931134	up
GO:0006898	receptor-mediated endocytosis	20	323	176	16775	1.97E-10	1.29E-07	5.901702786	9.706365	up
GO:0022617	extracellular matrix disassembly	16	323	120	16775	1.25E-09	6.18E-07	6.924664603	8.901409	up
GO:0030199	collagen fibril organization	10	323	41	16775	4.03E-09	1.59E-06	12.6670694	8.395039	up
GO:0007155	cell adhesion	36	323	640	16775	8.41E-09	2.76E-06	2.921342879	8.074957	up
GO:0001523	retinoid metabolic process	10	323	61	16775	2.3E-07	6.48E-05	8.513931889	6.637683	up
GO:0042157	lipoprotein metabolic process	8	323	39	16775	6.34E-07	0.000156	10.65333016	6.198015	up
GO:0007263	nitric oxide mediated signal transduction	6	323	18	16775	7.43E-07	0.000163	17.31166151	6.128981	up
GO:0034374	low-density lipoprotein particle remodeling	5	323	11	16775	1.08E-06	0.000212	23.60681115	5.96737	up
GO:0008203	cholesterol metabolic process	11	323	94	16775	1.89E-06	0.000339	6.077498189	5.72263	up
GO:0042632	cholesterol homeostasis	9	323	64	16775	3.53E-06	0.00058	7.303357198	5.451701	up
GO:0007565	female pregnancy	11	323	102	16775	4.26E-06	0.000646	5.600831664	5.370318	up
GO:0010873	positive regulation of cholesterol esterification	4	323	8	16775	8.89E-06	0.00125	25.96749226	5.051297	up
GO:0007411	axon guidance	26	323	543	16775	1.99E-05	0.002567	2.486758006	4.701624	up
GO:0007603	phototransduction, visible light	10	323	99	16775	2.09E-05	0.002567	5.245958032	4.680685	up
GO:0002576	platelet degranulation	9	323	84	16775	3.37E-05	0.003906	5.564462627	4.472015	up
GO:0071230	cellular response to amino acid stimulus	7	323	49	16775	3.94E-05	0.004315	7.419283503	4.403985	up
GO:0031100	organ regeneration	8	323	69	16775	5.22E-05	0.005408	6.021447481	4.282436	up
GO:0001501	skeletal system development	12	323	159	16775	6.03E-05	0.005935	3.919621473	4.219761	up
GO:0061304	retinal blood vessel morphogenesis	3	323	5	16775	6.87E-05	0.006444	31.16099071	4.16286	up
GO:0014070	response to organic cyclic compound	11	323	140	16775	8.49E-05	0.007596	4.080605927	4.071217	up
GO:0008206	bile acid metabolic process	6	323	40	16775	0.000108	0.009132	7.790247678	3.968509	up
GO:0035909	aorta morphogenesis	4	323	14	16775	0.000116	0.009132	14.83856701	3.935759	up
GO:0033700	phospholipid efflux	4	323	14	16775	0.000116	0.009132	14.83856701	3.935759	up
GO:0034384	high-density lipoprotein particle clearance	3	323	6	16775	0.000135	0.009528	25.96749226	3.868077	up
GO:0044332	Wnt signaling pathway involved in dorsal/ventral axis specificatioı	3	323	6	16775	0.000135	0.009528	25.96749226	3.868077	up
GO:0042158	lipoprotein biosynthetic process	3	323	6	16775	0.000135	0.009528	25.96749226	3.868077	up
GO:0007568	aging	12	323	175	16775	0.000151	0.01022	3.561256081	3.8215	up
GO:0034375	high-density lipoprotein particle remodeling	4	323	15	16775	0.000156	0.01022	13.84932921	3.80768	up
GO:0016525	negative regulation of angiogenesis	7	323	61	16775	0.000164	0.010447	5.959752322	3.783908	up
GO:0033344	cholesterol efflux	5	323	28	16775	0.000175	0.010781	9.274104379	3.756449	up
GO:0014012	peripheral nervous system axon regeneration	3	323	7	16775	0.000234	0.013536	22.25785051	3.631281	up
GO:0019441	tryptophan catabolic process to kynurenine	3	323	7	16775	0.000234	0.013536	22.25785051	3.631281	up
GO:0001525	angiogenesis	14	323	244	16775	0.000284	0.015988	2.979876161	3.546381	up
GO:0030168	platelet activation	13	323	216	16775	0.000294	0.016105	3.125716661	3.530972	up
GO:0043691	reverse cholesterol transport	4	323	18	16775	0.000333	0.016541	11.54110767	3.476922	up
GO:2000379	positive regulation of reactive oxygen species metabolic process	5	323	32	16775	0.000337	0.016541	8.114841331	3.472284	up
GO:0050848	regulation of calcium-mediated signaling	3	323	8	16775	0.000369	0.016541	19.4756192	3.433398	up
GO:0034380	high-density lipoprotein particle assembly	3	323	8	16775	0.000369	0.016541	19.4756192	3.433398	up
GO:0032488	Cdc42 protein signal transduction	2	323	2	16775	0.00037	0.016541	51.93498452	3.432241	up
GO:0010758	regulation of macrophage chemotaxis	2	323	2	16775	0.00037	0.016541	51.93498452	3.432241	up
GO:0032803	gulation of low-density lipoprotein particle receptor catabolic proc	2	323	2	16775	0.00037	0.016541	51.93498452	3.432241	up
GO:0032805	e regulation of low-density lipoprotein particle receptor catabolic ן	2	323	2	16775	0.00037	0.016541	51.93498452	3.432241	up
GO:0009612	response to mechanical stimulus	7	323	70	16775	0.00039	0.017062	5.193498452	3.40901	up
GO:0008217	regulation of blood pressure	7	323	72	16775	0.000464	0.01985	5.049234606	3.333732	up
GO:0071300	cellular response to retinoic acid	7	323	73	16775	0.000505	0.021139	4.980067009	3.29706	up
GO:0010951	negative regulation of endopeptidase activity	10	323	146	16775	0.000535	0.021465	3.557190721	3.271261	up
GO:0034372	very-low-density lipoprotein particle remodeling	3	323	9	16775	0.000545	0.021465	17.31166151	3.263536	up
GO:0031325	positive regulation of cellular metabolic process	3	323	9	16775	0.000545	0.021465	17.31166151	3.263536	up
GO:0007160	cell-matrix adhesion	8	323	101	16775	0.000742	0.027621	4.11366214	3.129862	up
GO:0031102	neuron projection regeneration	3	323	10	16775	0.000768	0.027621	15.58049536	3.114859	up
GO:0045723	positive regulation of fatty acid biosynthetic process	3	323	10	16775	0.000768	0.027621	15.58049536	3.114859	up
GO:0006569	tryptophan catabolic process	3	323	10	16775	0.000768	0.027621	15.58049536	3.114859	up
GO:0006935	chemotaxis	10	323	153	16775	0.000772	0.027621	3.394443433	3.112645	up
GO:0010466	negative regulation of peptidase activity	9	323	127	16775	0.00079	0.027769	3.680431974	3.102498	up
GO:0042060	wound healing	8	323	103	16775	0.000844	0.029156	4.033785205	3.07364	up
GO:0060395	SMAD protein signal transduction	6	323	59	16775	0.000929	0.031239	5.28152385	3.03184	up
GO:0055072	iron ion homeostasis	5	323	40	16775	0.000971	0.031239	6.491873065	3.012666	up
GO:0006805	xenobiotic metabolic process	10	323	158	16775	0.000988	0.031239	3.287024337	3.005286	up
GO:0031639	plasminogen activation	3	323	11	16775	0.00104	0.031239	14.16408669	2.982775	up
GO:0070208	protein heterotrimerization	3	323	11	16775	0.00104	0.031239	14.16408669	2.982775	up
GO:0030301	cholesterol transport	4	323	24	16775	0.001057	0.031239	8.655830753	2.975823	up
GO:0006953	acute-phase response	5	323	41	16775	0.001089	0.031239	6.333534698	2.962996	up
GO:0034395	ion of transcription from RNA polymerase II promoter in response	2	323	3	16775	0.001095	0.031239	34.62332301	2.960696	up
GO:0003331	positive regulation of extracellular matrix constituent secretion	2	323	3	16775	0.001095	0.031239	34.62332301	2.960696	up
GO:0010903	gative regulation of very-low-density lipoprotein particle remodeli	2	323	3	16775	0.001095	0.031239	34.62332301	2.960696	up
GO:0002034	regulation of blood vessel size by renin-angiotensin	2	323	3	16775	0.001095	0.031239	34.62332301	2.960696	up
GO:0043420	anthranilate metabolic process	2	323	3	16775	0.001095	0.031239	34.62332301	2.960696	up
GO:0001666	response to hypoxia	11	323	190	16775	0.001165	0.032775	3.006762262	2.93361	up
GO:0032496	response to lipopolysaccharide	11	323	195	16775	0.001436	0.039465	2.929665793	2.84286	up
GO:0032967	positive regulation of collagen biosynthetic process	4	323	26	16775	0.001443	0.039465	7.989997618	2.840701	up
GO:0043524	negative regulation of neuron apoptotic process	9	323	139	16775	0.001495	0.04032	3.362696839	2.825398	up
GO:0018108	peptidyl-tyrosine phosphorylation	9	323	142	16775	0.001733	0.044334	3.291653948	2.761153	up
GO:0043206	extracellular fibril organization	3	323	13	16775	0.001753	0.044334	11.98499643	2.756311	up
GO:0014912	negative regulation of smooth muscle cell migration	3	323	13	16775	0.001753	0.044334	11.98499643	2.756311	up
GO:0009267	cellular response to starvation	5	323	46	16775	0.001842	0.044334	5.645107013	2.734757	up
GO:0035987	endodermal cell differentiation	4	323	28	16775	0.001918	0.044334	7.419283503	2.71725	up
GO:0042472	inner ear morphogenesis	6	323	68	16775	0.001954	0.044334	4.582498634	2.709038	up
GO:0006865	amino acid transport	5	323	47	16775	0.002029	0.044334	5.524998353	2.6927	up
GO:0042493	response to drug	16	323	368	16775	0.002152	0.044334	2.258042805	2.667062	up
GO:0060192	negative regulation of lipase activity	2	323	4	16775	0.002162	0.044334	25.96749226	2.665234	up
GO:0032489	regulation of Cdc42 protein signal transduction	2	323	4	16775	0.002162	0.044334	25.96749226	2.665234	up
GO:0034638	phosphatidylcholine catabolic process	2	323	4	16775	0.002162	0.044334	25.96749226	2.665234	up
GO:0048144	fibroblast proliferation	2	323	4	16775	0.002162	0.044334	25.96749226	2.665234	up
GO:0051387	egative regulation of neurotrophin TRK receptor signaling pathwa	2	323	4	16775	0.002162	0.044334	25.96749226	2.665234	up
GO:0014706	striated muscle tissue development	2	323	4	16775	0.002162	0.044334	25.96749226	2.665234	up
GO:0034354	'de novo' NAD biosynthetic process from tryptophan	2	323	4	16775	0.002162	0.044334	25.96749226	2.665234	up
GO:0072378	blood coagulation, fibrin clot formation	2	323	4	16775	0.002162	0.044334	25.96749226	2.665234	up
GO:0010989	negative regulation of low-density lipoprotein particle clearance	2	323	4	16775	0.002162	0.044334	25.96749226	2.665234	up
GO:0021545	cranial nerve development	2	323	4	16775	0.002162	0.044334	25.96749226	2.665234	up
GO:0048659	smooth muscle cell proliferation	2	323	4	16775	0.002162	0.044334	25.96749226	2.665234	up
GO:2000272	negative regulation of receptor activity	2	323	4	16775	0.002162	0.044334	25.96749226	2.665234	up
GO:0019805	quinolinate biosynthetic process	2	323	4	16775	0.002162	0.044334	25.96749226	2.665234	up
GO:0042159	lipoprotein catabolic process	2	323	4	16775	0.002162	0.044334	25.96749226	2.665234	up
GO:0035813	regulation of renal sodium excretion	2	323	4	16775	0.002162	0.044334	25.96749226	2.665234	up
GO:0030204	chondroitin sulfate metabolic process	5	323	49	16775	0.002445	0.049627	5.299488216	2.611756	up
GO:0019433	triglyceride catabolic process	4	323	30	16775	0.00249	0.050034	6.924664603	2.603758	up
GO:0071354	cellular response to interleukin-6	3	323	15	16775	0.00271	0.053895	10.3869969	2.567061	up
GO:0008202	steroid metabolic process	8	323	124	16775	0.002764	0.054425	3.350644163	2.558444	up
GO:0046627	negative regulation of insulin receptor signaling pathway	4	323	31	16775	0.002816	0.054905	6.701288325	2.550311	up
GO:0055085	transmembrane transport	28	323	829	16775	0.002857	0.055144	1.754136992	2.544147	up
GO:0045444	fat cell differentiation	6	323	74	16775	0.003005	0.057444	4.210944691	2.522163	up

GO:2001244	positive regulation of intrinsic apoptotic signaling pathway	4	323	32	16775	0.003171	0.060027	6.491873065	2.498865	up
GO:2000310	gulation of N-methyl-D-aspartate selective glutamate receptor activ	3	323	16	16775	0.003288	0.061657	9.737809598	2.483073	up
GO:0001503	ossification	7	323	101	16775	0.003375	0.061976	3.599454373	2.471706	up
GO:0030300	regulation of intestinal cholesterol absorption	2	323	5	16775	0.003557	0.061976	20.77399381	2.448944	up
GO:0018158	protein oxidation	2	323	5	16775	0.003557	0.061976	20.77399381	2.448944	up
GO:0016576	histone dephosphorylation	2	323	5	16775	0.003557	0.061976	20.77399381	2.448944	up
GO:0097114	N-methyl-D-aspartate receptor clustering	2	323	5	16775	0.003557	0.061976	20.77399381	2.448944	up
GO:0034755	iron ion transmembrane transport	2	323	5	16775	0.003557	0.061976	20.77399381	2.448944	up
GO:2001171	positive regulation of ATP biosynthetic process	2	323	5	16775	0.003557	0.061976	20.77399381	2.448944	up
GO:0044320	cellular response to leptin stimulus	2	323	5	16775	0.003557	0.061976	20.77399381	2.448944	up
GO:0043627	response to estrogen	6	323	77	16775	0.003666	0.06277	4.046881911	2.435794	up
GO:0001558	regulation of cell growth	6	323	77	16775	0.003666	0.06277	4.046881911	2.435794	up
GO:0007229	integrin-mediated signaling pathway	7	323	103	16775	0.003764	0.06389	3.529562055	2.424353	up
GO:0043623	cellular protein complex assembly	3	323	17	16775	0.003936	0.066241	9.164997268	2.404934	up
GO:0032526	response to retinoic acid	5	323	55	16775	0.004062	0.067775	4.721362229	2.391295	up
GO:0046849	bone remodeling	3	323	18	16775	0.004657	0.077049	8.655830753	2.331929	up
GO:0048839	inner ear development	5	323	57	16775	0.004739	0.077757	4.555700397	2.324325	up
GO:0001649	osteoblast differentiation	7	323	108	16775	0.004885	0.078925	3.366156404	2.311156	up
GO:0060325	face morphogenesis	4	323	36	16775	0.00489	0.078925	5.770553836	2.310673	up
GO:0032964	collagen biosynthetic process	2	323	6	16775	0.005267	0.080399	17.31166151	2.278403	up
GO:0018206	peptidyl-methionine modification	2	323	6	16775	0.005267	0.080399	17.31166151	2.278403	up
GO:2000650	egative regulation of sodium ion transmembrane transporter activi	2	323	6	16775	0.005267	0.080399	17.31166151	2.278403	up
GO:0002740	gative regulation of cytokine secretion involved in immune respon	2	323	6	16775	0.005267	0.080399	17.31166151	2.278403	up
GO:0034382	chylomicron remnant clearance	2	323	6	16775	0.005267	0.080399	17.31166151	2.278403	up
GO:0001957	intramembranous ossification	2	323	6	16775	0.005267	0.080399	17.31166151	2.278403	up
GO:0046006	regulation of activated T cell proliferation	2	323	6	16775	0.005267	0.080399	17.31166151	2.278403	up
GO:0033077	T cell differentiation in thymus	4	323	37	16775	0.005401	0.081807	5.614592921	2.267509	up
GO:2000463	positive regulation of excitatory postsynaptic membrane potential	3	323	19	16775	0.005452	0.081942	8.200260714	2.263466	up
GO:0006641	triglyceride metabolic process	4	323	38	16775	0.005947	0.088038	5.466840476	2.225724	up
GO:0050714	positive regulation of protein secretion	4	323	38	16775	0.005947	0.088038	5.466840476	2.225724	up
GO:0048661	positive regulation of smooth muscle cell proliferation	5	323	61	16775	0.006327	0.092974	4.256965944	2.19878	up
GO:0016477	cell migration	10	323	206	16775	0.006727	0.095574	2.521115753	2.172211	up
GO:0001568	blood vessel development	5	323	62	16775	0.006776	0.095574	4.188305203	2.169004	up
GO:0060070	canonical Wnt signaling pathway	6	323	88	16775	0.007034	0.095574	3.541021672	2.152796	up
GO:0001895	retina homeostasis	4	323	40	16775	0.007145	0.095574	5.193498452	2.145996	up
GO:0042730	fibrinolysis	3	323	21	16775	0.007273	0.095574	7.419283503	2.13826	up
GO:0097503	sialylation	3	323	21	16775	0.007273	0.095574	7.419283503	2.13826	up
GO:0016125	sterol metabolic process	3	323	21	16775	0.007273	0.095574	7.419283503	2.13826	up
GO:0033137	negative regulation of peptidyl-serine phosphorylation	3	323	21	16775	0.007273	0.095574	7.419283503	2.13826	up
GO:0019835	cytolysis	3	323	21	16775	0.007273	0.095574	7.419283503	2.13826	up
GO:0035902	response to immobilization stress	3	323	21	16775	0.007273	0.095574	7.419283503	2.13826	up
GO:0030193	regulation of blood coagulation	3	323	21	16775	0.007273	0.095574	7.419283503	2.13826	up
GO:0071281	cellular response to iron ion	2	323	7	16775	0.007281	0.095574	14.83856701	2.137816	up
GO:0035524	proline transmembrane transport	2	323	7	16775	0.007281	0.095574	14.83856701	2.137816	up
GO:0042448	progesterone metabolic process	2	323	7	16775	0.007281	0.095574	14.83856701	2.137816	up
GO:0042167	heme catabolic process	2	323	7	16775	0.007281	0.095574	14.83856701	2.137816	up
GO:0034115	negative regulation of heterotypic cell-cell adhesion	2	323	7	16775	0.007281	0.095574	14.83856701	2.137816	up
GO:0032570	response to progesterone	4	323	41	16775	0.0078	0.101707	5.066827758	2.107919	up
GO:0006699	bile acid biosynthetic process	3	323	22	16775	0.008303	0.106859	7.082043344	2.080743	up
GO:0006796	phosphate-containing compound metabolic process	3	323	22	16775	0.008303	0.106859	7.082043344	2.080743	up
GO:0006024	glycosaminoglycan biosynthetic process	4	323	42	16775	0.008493	0.107884	4.946189002	2.070955	up
GO:0045600	positive regulation of fat cell differentiation	4	323	42	16775	0.008493	0.107884	4.946189002	2.070955	up
GO:0007275	multicellular organismal development	33	323	1110	16775	0.008665	0.108937	1.544013053	2.062214	up
GO:0051384	response to glucocorticoid	6	323	92	16775	0.008686	0.108937	3.387064208	2.061171	up
GO:0045921	positive regulation of exocytosis	3	323	23	16775	0.009415	0.11	6.774128416	2.026191	up
GO:0045879	negative regulation of smoothened signaling pathway	3	323	23	16775	0.009415	0.11	6.774128416	2.026191	up
GO:0061072	iris morphogenesis	2	323	8	16775	0.009585	0.11	12.98374613	2.01841	up
GO:0031659	ependent protein serine/threonine kinase activity involved in G1/S	2	323	8	16775	0.009585	0.11	12.98374613	2.01841	up
GO:0010656	negative regulation of muscle cell apoptotic process	2	323	8	16775	0.009585	0.11	12.98374613	2.01841	up
GO:0034383	low-density lipoprotein particle clearance	2	323	8	16775	0.009585	0.11	12.98374613	2.01841	up
GO:0014824	artery smooth muscle contraction	2	323	8	16775	0.009585	0.11	12.98374613	2.01841	up
GO:0042473	outer ear morphogenesis	2	323	8	16775	0.009585	0.11	12.98374613	2.01841	up
GO:0070233	negative regulation of T cell apoptotic process	2	323	8	16775	0.009585	0.11	12.98374613	2.01841	up
GO:0060059	embryonic retina morphogenesis in camera-type eye	2	323	8	16775	0.009585	0.11	12.98374613	2.01841	up
GO:0045647	negative regulation of erythrocyte differentiation	2	323	8	16775	0.009585	0.11	12.98374613	2.01841	up
GO:0033629	negative regulation of cell adhesion mediated by integrin	2	323	8	16775	0.009585	0.11	12.98374613	2.01841	up
GO:0015824	proline transport	2	323	8	16775	0.009585	0.11	12.98374613	2.01841	up
GO:0090277	positive regulation of peptide hormone secretion	2	323	8	16775	0.009585	0.11	12.98374613	2.01841	up
GO:0009636	response to toxic substance	6	323	94	16775	0.009609	0.11	3.314999012	2.017326	up
GO:0030334	regulation of cell migration	5	323	69	16775	0.01056	0.117351	3.763404675	1.976351	up
GO:0070098	chemokine-mediated signaling pathway	5	323	69	16775	0.01056	0.117351	3.763404675	1.976351	up
GO:0006956	complement activation	5	323	69	16775	0.01056	0.117351	3.763404675	1.976351	up
GO:0070328	triglyceride homeostasis	3	323	24	16775	0.010609	0.117351	6.491873065	1.974338	up
GO:0061098	positive regulation of protein tyrosine kinase activity	3	323	24	16775	0.010609	0.117351	6.491873065	1.974338	up
GO:0050804	regulation of synaptic transmission	3	323	24	16775	0.010609	0.117351	6.491873065	1.974338	up
GO:0030522	intracellular receptor signaling pathway	4	323	45	16775	0.010809	0.117742	4.616443068	1.966206	up
GO:0050679	positive regulation of epithelial cell proliferation	5	323	70	16775	0.011198	0.117742	3.709641751	1.950878	up
GO:0006656	phosphatidylcholine biosynthetic process	3	323	25	16775	0.011886	0.117742	6.232198142	1.924954	up
GO:1902042	gulation of extrinsic apoptotic signaling pathway via death domai	3	323	25	16775	0.011886	0.117742	6.232198142	1.924954	up
GO:0006954	inflammatory response	14	323	367	16775	0.012008	0.117742	1.981171072	1.920525	up
GO:0060586	multicellular organismal iron ion homeostasis	2	323	9	16775	0.012168	0.117742	11.54110767	1.914789	up
GO:0006688	glycosphingolipid biosynthetic process	2	323	9	16775	0.012168	0.117742	11.54110767	1.914789	up
GO:0061049	cell growth involved in cardiac muscle cell development	2	323	9	16775	0.012168	0.117742	11.54110767	1.914789	up
GO:0043589	skin morphogenesis	2	323	9	16775	0.012168	0.117742	11.54110767	1.914789	up
GO:0097120	receptor localization to synapse	2	323	9	16775	0.012168	0.117742	11.54110767	1.914789	up
GO:0060603	mammary gland duct morphogenesis	2	323	9	16775	0.012168	0.117742	11.54110767	1.914789	up
GO:0010884	positive regulation of lipid storage	2	323	9	16775	0.012168	0.117742	11.54110767	1.914789	up
GO:0070374	positive regulation of ERK1 and ERK2 cascade	8	323	160	16775	0.012419	0.117742	2.596749226	1.90592	up
GO:0048705	skeletal system morphogenesis	4	323	47	16775	0.012559	0.117742	4.419998683	1.90103	up
GO:0009311	oligosaccharide metabolic process	3	323	26	16775	0.013248	0.117742	5.992498214	1.877836	up
GO:0045880	positive regulation of smoothened signaling pathway	3	323	26	16775	0.013248	0.117742	5.992498214	1.877836	up
GO:0006027	glycosaminoglycan catabolic process	3	323	26	16775	0.013248	0.117742	5.992498214	1.877836	up
GO:0006879	cellular iron ion homeostasis	5	323	74	16775	0.014011	0.117742	3.509120576	1.853521	up
GO:0030325	adrenal gland development	3	323	27	16775	0.014696	0.117742	5.770553836	1.832804	up
GO:2000377	regulation of reactive oxygen species metabolic process	3	323	27	16775	0.014696	0.117742	5.770553836	1.832804	up
GO:0015721	bile acid and bile salt transport	3	323	27	16775	0.014696	0.117742	5.770553836	1.832804	up
GO:0045165	cell fate commitment	5	323	75	16775	0.014783	0.117742	3.462332301	1.830252	up
GO:0046689	response to mercury ion	2	323	10	16775	0.015018	0.117742	10.3869969	1.823393	up
GO:0051347	positive regulation of transferase activity	2	323	10	16775	0.015018	0.117742	10.3869969	1.823393	up
GO:2000505	regulation of energy homeostasis	2	323	10	16775	0.015018	0.117742	10.3869969	1.823393	up
GO:0070886	positive regulation of calcineurin-NFAT signaling cascade	2	323	10	16775	0.015018	0.117742	10.3869969	1.823393	up
GO:0006835	dicarboxylic acid transport	2	323	10	16775	0.015018	0.117742	10.3869969	1.823393	up
GO:0051006	positive regulation of lipoprotein lipase activity	2	323	10	16775	0.015018	0.117742	10.3869969	1.823393	up
GO:0043534	blood vessel endothelial cell migration	2	323	10	16775	0.015018	0.117742	10.3869969	1.823393	up
GO:0006930	substrate-dependent cell migration, cell extension	2	323	10	16775	0.015018	0.117742	10.3869969	1.823393	up

GO:0051918	negative regulation of fibrinolysis	2	323	10	16775	0.015018	0.117742	10.3869969	1.823393	up
GO:0034116	positive regulation of heterotypic cell-cell adhesion	2	323	10	16775	0.015018	0.117742	10.3869969	1.823393	up
GO:0043434	response to peptide hormone	5	323	76	16775	0.015582	0.117742	3.416775297	1.807386	up
GO:0007596	blood coagulation	17	323	495	16775	0.015761	0.117742	1.783625731	1.80243	up
GO:0008406	gonad development	3	323	28	16775	0.016229	0.117742	5.564462627	1.7897	up
GO:0045840	positive regulation of mitosis	3	323	29	16775	0.017849	0.117742	5.372584606	1.748384	up
GO:1900026	positive regulation of substrate adhesion-dependent cell spreading	3	323	29	16775	0.017849	0.117742	5.372584606	1.748384	up
GO:0030828	positive regulation of cGMP biosynthetic process	2	323	11	16775	0.018124	0.117742	9.442724458	1.741748	up
GO:0006707	cholesterol catabolic process	2	323	11	16775	0.018124	0.117742	9.442724458	1.741748	up
GO:0021952	central nervous system projection neuron axonogenesis	2	323	11	16775	0.018124	0.117742	9.442724458	1.741748	up
GO:1902475	L-alpha-amino acid transmembrane transport	2	323	11	16775	0.018124	0.117742	9.442724458	1.741748	up
GO:0046325	negative regulation of glucose import	2	323	11	16775	0.018124	0.117742	9.442724458	1.741748	up
GO:0002011	morphogenesis of an epithelial sheet	2	323	11	16775	0.018124	0.117742	9.442724458	1.741748	up
GO:0042551	neuron maturation	2	323	11	16775	0.018124	0.117742	9.442724458	1.741748	up
GO:0098609	cell-cell adhesion	2	323	11	16775	0.018124	0.117742	9.442724458	1.741748	up
GO:0061303	cornea development in camera-type eye	2	323	11	16775	0.018124	0.117742	9.442724458	1.741748	up
GO:0006081	cellular aldehyde metabolic process	2	323	11	16775	0.018124	0.117742	9.442724458	1.741748	up
GO:0045603	positive regulation of endothelial cell differentiation	2	323	11	16775	0.018124	0.117742	9.442724458	1.741748	up
GO:0043408	regulation of MAPK cascade	4	323	53	16775	0.018858	0.117742	3.919621473	1.724503	up
GO:0071250	cellular response to nitrite	1	323	1	16775	0.019255	0.117742	51.93498452	1.71546	up
GO:0051641	cellular localization	1	323	1	16775	0.019255	0.117742	51.93498452	1.71546	up
GO:0061444	endocardial cushion cell development	1	323	1	16775	0.019255	0.117742	51.93498452	1.71546	up
GO:0051651	maintenance of location in cell	1	323	1	16775	0.019255	0.117742	51.93498452	1.71546	up
GO:1902999	negative regulation of phospholipid efflux	1	323	1	16775	0.019255	0.117742	51.93498452	1.71546	up
GO:0060588	negative regulation of lipoprotein lipid oxidation	1	323	1	16775	0.019255	0.117742	51.93498452	1.71546	up
GO:1903001	negative regulation of lipid transport across blood brain barrier	1	323	1	16775	0.019255	0.117742	51.93498452	1.71546	up
GO:1903002	positive regulation of lipid transport across blood brain barrier	1	323	1	16775	0.019255	0.117742	51.93498452	1.71546	up
GO:0032960	regulation of inositol trisphosphate biosynthetic process	1	323	1	16775	0.019255	0.117742	51.93498452	1.71546	up
GO:0006214	thymidine catabolic process	1	323	1	16775	0.019255	0.117742	51.93498452	1.71546	up
GO:0061050	gulation of cell growth involved in cardiac muscle cell developme	1	323	1	16775	0.019255	0.117742	51.93498452	1.71546	up
GO:0036060	slit diaphragm assembly	1	323	1	16775	0.019255	0.117742	51.93498452	1.71546	up
GO:0032514	positive regulation of protein phosphatase type 2B activity	1	323	1	16775	0.019255	0.117742	51.93498452	1.71546	up
GO:0021859	pyramidal neuron differentiation	1	323	1	16775	0.019255	0.117742	51.93498452	1.71546	up
GO:0010712	regulation of collagen metabolic process	1	323	1	16775	0.019255	0.117742	51.93498452	1.71546	up
GO:0043618	on of transcription from RNA polymerase II promoter in response	1	323	1	16775	0.019255	0.117742	51.93498452	1.71546	up
GO:0052565	response to defense-related host nitric oxide production	1	323	1	16775	0.019255	0.117742	51.93498452	1.71546	up
GO:0006145	purine nucleobase catabolic process	1	323	1	16775	0.019255	0.117742	51.93498452	1.71546	up
GO:0010635	regulation of mitochondrial fusion	1	323	1	16775	0.019255	0.117742	51.93498452	1.71546	up
GO:1902078	positive regulation of lateral motor column neuron migration	1	323	1	16775	0.019255	0.117742	51.93498452	1.71546	up
GO:1902952	positive regulation of dendritic spine maintenance	1	323	1	16775	0.019255	0.117742	51.93498452	1.71546	up
GO:1902951	negative regulation of dendritic spine maintenance	1	323	1	16775	0.019255	0.117742	51.93498452	1.71546	up
GO:1902947	regulation of tau-protein kinase activity	1	323	1	16775	0.019255	0.117742	51.93498452	1.71546	up
GO:0010255	glucose mediated signaling pathway	1	323	1	16775	0.019255	0.117742	51.93498452	1.71546	up
GO:1901630	negative regulation of presynaptic membrane organization	1	323	1	16775	0.019255	0.117742	51.93498452	1.71546	up
GO:1901628	positive regulation of postsynaptic membrane organization	1	323	1	16775	0.019255	0.117742	51.93498452	1.71546	up
GO:1901627	negative regulation of postsynaptic membrane organization	1	323	1	16775	0.019255	0.117742	51.93498452	1.71546	up
GO:0070346	positive regulation of fat cell proliferation	1	323	1	16775	0.019255	0.117742	51.93498452	1.71546	up
GO:0034276	kynurenic acid biosynthetic process	1	323	1	16775	0.019255	0.117742	51.93498452	1.71546	up
GO:0002678	positive regulation of chronic inflammatory response	1	323	1	16775	0.019255	0.117742	51.93498452	1.71546	up
GO:0010286	heat acclimation	1	323	1	16775	0.019255	0.117742	51.93498452	1.71546	up
GO:0060981	cell migration involved in coronary angiogenesis	1	323	1	16775	0.019255	0.117742	51.93498452	1.71546	up
GO:0046687	response to chromate	1	323	1	16775	0.019255	0.117742	51.93498452	1.71546	up
GO:0061573	actin filament bundle retrograde transport	1	323	1	16775	0.019255	0.117742	51.93498452	1.71546	up
GO:1901585	regulation of acid-sensing ion channel activity	1	323	1	16775	0.019255	0.117742	51.93498452	1.71546	up
GO:0006714	sesquiterpenoid metabolic process	1	323	1	16775	0.019255	0.117742	51.93498452	1.71546	up
GO:0030826	regulation of cGMP biosynthetic process	1	323	1	16775	0.019255	0.117742	51.93498452	1.71546	up
GO:0070471	uterine smooth muscle contraction	1	323	1	16775	0.019255	0.117742	51.93498452	1.71546	up
GO:0071357	cellular response to type I interferon	1	323	1	16775	0.019255	0.117742	51.93498452	1.71546	up
GO:0060695	negative regulation of cholesterol transporter activity	1	323	1	16775	0.019255	0.117742	51.93498452	1.71546	up
GO:0061163	endoplasmic reticulum polarization	1	323	1	16775	0.019255	0.117742	51.93498452	1.71546	up
GO:0021511	spinal cord patterning	1	323	1	16775	0.019255	0.117742	51.93498452	1.71546	up
GO:0048178	egative regulation of hepatocyte growth factor biosynthetic proces	1	323	1	16775	0.019255	0.117742	51.93498452	1.71546	up
GO:0001914	regulation of T cell mediated cytotoxicity	1	323	1	16775	0.019255	0.117742	51.93498452	1.71546	up
GO:0072275	metanephric glomerulus morphogenesis	1	323	1	16775	0.019255	0.117742	51.93498452	1.71546	up
GO:0010752	regulation of cGMP-mediated signaling	1	323	1	16775	0.019255	0.117742	51.93498452	1.71546	up
GO:0097053	L-kynurenine catabolic process	1	323	1	16775	0.019255	0.117742	51.93498452	1.71546	up
GO:0031275	regulation of lateral pseudopodium assembly	1	323	1	16775	0.019255	0.117742	51.93498452	1.71546	up
GO:0002765	immune response-inhibiting signal transduction	1	323	1	16775	0.019255	0.117742	51.93498452	1.71546	up
GO:0034373	intermediate-density lipoprotein particle remodeling	1	323	1	16775	0.019255	0.117742	51.93498452	1.71546	up
GO:1900139	negative regulation of arachidonic acid secretion	1	323	1	16775	0.019255	0.117742	51.93498452	1.71546	up
GO:0060354	negative regulation of cell adhesion molecule production	1	323	1	16775	0.019255	0.117742	51.93498452	1.71546	up
GO:0060376	positive regulation of mast cell differentiation	1	323	1	16775	0.019255	0.117742	51.93498452	1.71546	up
GO:0048685	ive regulation of collateral sprouting of intact axon in response to	1	323	1	16775	0.019255	0.117742	51.93498452	1.71546	up
GO:0060345	spleen trabecula formation	1	323	1	16775	0.019255	0.117742	51.93498452	1.71546	up
GO:0051866	general adaptation syndrome	1	323	1	16775	0.019255	0.117742	51.93498452	1.71546	up
GO:0036269	swimming behavior	1	323	1	16775	0.019255	0.117742	51.93498452	1.71546	up
GO:0097242	beta-amyloid clearance	1	323	1	16775	0.019255	0.117742	51.93498452	1.71546	up
GO:0072377	blood coagulation, common pathway	1	323	1	16775	0.019255	0.117742	51.93498452	1.71546	up
GO:0033986	response to methanol	1	323	1	16775	0.019255	0.117742	51.93498452	1.71546	up
GO:0001998	diated vasoconstriction involved in regulation of systemic arterial	1	323	1	16775	0.019255	0.117742	51.93498452	1.71546	up
GO:0001999	low involved in circulatory renin-angiotensin regulation of system	1	323	1	16775	0.019255	0.117742	51.93498452	1.71546	up
GO:0086024	gic receptor signaling pathway involved in positive regulation of h	1	323	1	16775	0.019255	0.117742	51.93498452	1.71546	up
GO:0021572	rhombomere 6 development	1	323	1	16775	0.019255	0.117742	51.93498452	1.71546	up
GO:1901860	positive regulation of mitochondrial DNA metabolic process	1	323	1	16775	0.019255	0.117742	51.93498452	1.71546	up
GO:1901862	negative regulation of muscle tissue development	1	323	1	16775	0.019255	0.117742	51.93498452	1.71546	up
GO:1901863	positive regulation of muscle tissue development	1	323	1	16775	0.019255	0.117742	51.93498452	1.71546	up
GO:2000405	negative regulation of T cell migration	1	323	1	16775	0.019255	0.117742	51.93498452	1.71546	up
GO:0035789	metanephric mesenchymal cell migration	1	323	1	16775	0.019255	0.117742	51.93498452	1.71546	up
GO:1901843	positive regulation of high voltage-gated calcium channel activity	1	323	1	16775	0.019255	0.117742	51.93498452	1.71546	up
GO:2000452	egulation of CD8-positive, alpha-beta cytotoxic T cell extravasatio	1	323	1	16775	0.019255	0.117742	51.93498452	1.71546	up
GO:0001576	globoside biosynthetic process	1	323	1	16775	0.019255	0.117742	51.93498452	1.71546	up
GO:0001100	negative regulation of exit from mitosis	1	323	1	16775	0.019255	0.117742	51.93498452	1.71546	up
GO:2000261	negative regulation of blood coagulation, common pathway	1	323	1	16775	0.019255	0.117742	51.93498452	1.71546	up
GO:0003431	growth plate cartilage chondrocyte development	1	323	1	16775	0.019255	0.117742	51.93498452	1.71546	up
GO:1902632	positive regulation of membrane hyperpolarization	1	323	1	16775	0.019255	0.117742	51.93498452	1.71546	up
GO:0032802	low-density lipoprotein particle receptor catabolic process	1	323	1	16775	0.019255	0.117742	51.93498452	1.71546	up
GO:0051969	regulation of transmission of nerve impulse	1	323	1	16775	0.019255	0.117742	51.93498452	1.71546	up
GO:0070235	regulation of activation-induced cell death of T cells	1	323	1	16775	0.019255	0.117742	51.93498452	1.71546	up
GO:0060092	regulation of synaptic transmission, glycinergic	1	323	1	16775	0.019255	0.117742	51.93498452	1.71546	up
GO:1901382	regulation of chorionic trophoblast cell proliferation	1	323	1	16775	0.019255	0.117742	51.93498452	1.71546	up
GO:0051580	regulation of neurotransmitter uptake	1	323	1	16775	0.019255	0.117742	51.93498452	1.71546	up
GO:0010625	positive regulation of Schwann cell proliferation	1	323	1	16775	0.019255	0.117742	51.93498452	1.71546	up
GO:0038168	wth factor receptor signaling pathway via I-kappaB kinase/NF-ka	1	323	1	16775	0.019255	0.117742	51.93498452	1.71546	up
GO:0010534	regulation of activation of JAK2 kinase activity	1	323	1	16775	0.019255	0.117742	51.93498452	1.71546	up
GO:0032799	low-density lipoprotein receptor particle metabolic process	1	323	1	16775	0.019255	0.117742	51.93498452	1.71546	up

GO:0046520	sphingoid biosynthetic process	1	323	1	16775	0.019255	0.117742	51.93498452	1.71546	up
GO:0034516	response to vitamin B6	1	323	1	16775	0.019255	0.117742	51.93498452	1.71546	up
GO:0015828	tyrosine transport	1	323	1	16775	0.019255	0.117742	51.93498452	1.71546	up
GO:0034589	hydroxyproline transport	1	323	1	16775	0.019255	0.117742	51.93498452	1.71546	up
GO:0015826	threonine transport	1	323	1	16775	0.019255	0.117742	51.93498452	1.71546	up
GO:0034104	negative regulation of tissue remodeling	1	323	1	16775	0.019255	0.117742	51.93498452	1.71546	up
GO:0055065	metal ion homeostasis	1	323	1	16775	0.019255	0.117742	51.93498452	1.71546	up
GO:1902618	cellular response to fluoride	1	323	1	16775	0.019255	0.117742	51.93498452	1.71546	up
GO:0010171	body morphogenesis	1	323	1	16775	0.019255	0.117742	51.93498452	1.71546	up
GO:0045773	positive regulation of axon extension	3	323	30	16775	0.019556	0.117752	5.193498452	1.708728	up
GO:0031214	biomineral tissue development	3	323	30	16775	0.019556	0.117752	5.193498452	1.708728	up
GO:0010508	positive regulation of autophagy	3	323	30	16775	0.019556	0.117752	5.193498452	1.708728	up
GO:0001958	endochondral ossification	3	323	30	16775	0.019556	0.117752	5.193498452	1.708728	up
GO:0048662	negative regulation of smooth muscle cell proliferation	3	323	30	16775	0.019556	0.117752	5.193498452	1.708728	up
GO:0008285	negative regulation of cell proliferation	15	323	431	16775	0.020278	0.121728	1.80748206	1.692983	up
GO:0030324	lung development	6	323	111	16775	0.020433	0.122289	2.807296461	1.689661	up
GO:0006629	lipid metabolic process	17	323	512	16775	0.021126	0.122564	1.724403783	1.675193	up
GO:0043588	skin development	4	323	55	16775	0.021322	0.122564	3.777089783	1.671173	up
GO:0010718	positive regulation of epithelial to mesenchymal transition	3	323	31	16775	0.021349	0.122564	5.025966244	1.67062	up
GO:0001937	negative regulation of endothelial cell proliferation	3	323	31	16775	0.021349	0.122564	5.025966244	1.67062	up
GO:0042476	odontogenesis	3	323	31	16775	0.021349	0.122564	5.025966244	1.67062	up
GO:0010738	regulation of protein kinase A signaling	2	323	12	16775	0.021475	0.122564	8.655830753	1.668064	up
GO:2000573	positive regulation of DNA biosynthetic process	2	323	12	16775	0.021475	0.122564	8.655830753	1.668064	up
GO:0060391	positive regulation of SMAD protein import into nucleus	2	323	12	16775	0.021475	0.122564	8.655830753	1.668064	up
GO:2001222	regulation of neuron migration	2	323	12	16775	0.021475	0.122564	8.655830753	1.668064	up
GO:0002021	response to dietary excess	2	323	12	16775	0.021475	0.122564	8.655830753	1.668064	up
GO:0035025	positive regulation of Rho protein signal transduction	2	323	12	16775	0.021475	0.122564	8.655830753	1.668064	up
GO:0030208	dermatan sulfate biosynthetic process	2	323	12	16775	0.021475	0.122564	8.655830753	1.668064	up
GO:0019363	pyridine nucleotide biosynthetic process	2	323	12	16775	0.021475	0.122564	8.655830753	1.668064	up
GO:0006069	ethanol oxidation	2	323	12	16775	0.021475	0.122564	8.655830753	1.668064	up
GO:0031000	response to caffeine	2	323	12	16775	0.021475	0.122564	8.655830753	1.668064	up
GO:0055089	fatty acid homeostasis	2	323	12	16775	0.021475	0.122564	8.655830753	1.668064	up
GO:0032869	cellular response to insulin stimulus	5	323	83	16775	0.02199	0.125138	3.128613525	1.657781	up
GO:0002376	immune system process	14	323	398	16775	0.022555	0.127985	1.826858752	1.646759	up
GO:0014068	positive regulation of phosphatidylinositol 3-kinase signaling	4	323	56	16775	0.022624	0.12801	3.709641751	1.645424	up
GO:0007420	brain development	10	323	249	16775	0.022813	0.128709	2.08574235	1.641812	up
GO:0006869	lipid transport	6	323	114	16775	0.022959	0.128794	2.733420238	1.639044	up
GO:0032355	response to estradiol	6	323	114	16775	0.022959	0.128794	2.733420238	1.639044	up
GO:0016042	lipid catabolic process	6	323	115	16775	0.023846	0.133387	2.709651366	1.622591	up
GO:0051965	positive regulation of synapse assembly	4	323	57	16775	0.023974	0.13364	3.644560317	1.620256	up
GO:0044281	small molecule metabolic process	39	323	1465	16775	0.024027	0.13364	1.382569554	1.619306	up
GO:0045722	positive regulation of gluconeogenesis	2	323	13	16775	0.025061	0.135937	7.989997618	1.601	up
GO:0042953	lipoprotein transport	2	323	13	16775	0.025061	0.135937	7.989997618	1.601	up
GO:0009435	NAD biosynthetic process	2	323	13	16775	0.025061	0.135937	7.989997618	1.601	up
GO:0046889	positive regulation of lipid biosynthetic process	2	323	13	16775	0.025061	0.135937	7.989997618	1.601	up
GO:0010469	regulation of receptor activity	2	323	13	16775	0.025061	0.135937	7.989997618	1.601	up
GO:0060445	branching involved in salivary gland morphogenesis	2	323	13	16775	0.025061	0.135937	7.989997618	1.601	up
GO:0089711	L-glutamate transmembrane transport	2	323	13	16775	0.025061	0.135937	7.989997618	1.601	up
GO:0042574	retinal metabolic process	2	323	13	16775	0.025061	0.135937	7.989997618	1.601	up
GO:0001678	cellular glucose homeostasis	2	323	13	16775	0.025061	0.135937	7.989997618	1.601	up
GO:0050731	positive regulation of peptidyl-tyrosine phosphorylation	5	323	86	16775	0.025191	0.136266	3.019475844	1.598756	up
GO:0042542	response to hydrogen peroxide	4	323	58	16775	0.025372	0.136495	3.58172307	1.595648	up
GO:0043401	steroid hormone mediated signaling pathway	4	323	58	16775	0.025372	0.136495	3.58172307	1.595648	up
GO:0030203	glycosaminoglycan metabolic process	6	323	117	16775	0.025687	0.137813	2.663332539	1.590288	up
GO:0030879	mammary gland development	3	323	34	16775	0.027252	0.145416	4.582498634	1.564606	up
GO:0001569	patterning of blood vessels	3	323	34	16775	0.027252	0.145416	4.582498634	1.564606	up
GO:0042733	embryonic digit morphogenesis	4	323	60	16775	0.028312	0.150258	3.462332301	1.548033	up
GO:0007586	digestion	4	323	60	16775	0.028312	0.150258	3.462332301	1.548033	up
GO:0001822	kidney development	6	323	120	16775	0.028622	0.150391	2.596749226	1.543295	up
GO:0050900	leukocyte migration	6	323	120	16775	0.028622	0.150391	2.596749226	1.543295	up
GO:0071498	cellular response to fluid shear stress	2	323	14	16775	0.028871	0.150391	7.419283503	1.539532	up
GO:0010875	positive regulation of cholesterol efflux	2	323	14	16775	0.028871	0.150391	7.419283503	1.539532	up
GO:1902230	gulation of intrinsic apoptotic signaling pathway in response to DN	2	323	14	16775	0.028871	0.150391	7.419283503	1.539532	up
GO:0030207	chondroitin sulfate catabolic process	2	323	14	16775	0.028871	0.150391	7.419283503	1.539532	up
GO:0030195	negative regulation of blood coagulation	2	323	14	16775	0.028871	0.150391	7.419283503	1.539532	up
GO:0002027	regulation of heart rate	3	323	35	16775	0.029393	0.151407	4.451570102	1.531758	up
GO:0043507	positive regulation of JUN kinase activity	3	323	35	16775	0.029393	0.151407	4.451570102	1.531758	up
GO:0000302	response to reactive oxygen species	3	323	35	16775	0.029393	0.151407	4.451570102	1.531758	up
GO:0051591	response to cAMP	4	323	61	16775	0.029855	0.151407	3.405572755	1.52499	up
GO:0006508	proteolysis	20	323	659	16775	0.030553	0.151407	1.576175554	1.514952	up
GO:0010243	response to organonitrogen compound	3	323	36	16775	0.03162	0.151407	4.327915377	1.500032	up
GO:0046677	response to antibiotic	3	323	36	16775	0.03162	0.151407	4.327915377	1.500032	up
GO:0045907	positive regulation of vasoconstriction	3	323	36	16775	0.03162	0.151407	4.327915377	1.500032	up
GO:0050777	negative regulation of immune response	2	323	15	16775	0.032896	0.151407	6.924664603	1.482853	up
GO:0010744	positive regulation of macrophage derived foam cell differentiatior	2	323	15	16775	0.032896	0.151407	6.924664603	1.482853	up
GO:0009651	response to salt stress	2	323	15	16775	0.032896	0.151407	6.924664603	1.482853	up
GO:0006198	cAMP catabolic process	2	323	15	16775	0.032896	0.151407	6.924664603	1.482853	up
GO:0048856	anatomical structure development	2	323	15	16775	0.032896	0.151407	6.924664603	1.482853	up
GO:0030818	negative regulation of cAMP biosynthetic process	2	323	15	16775	0.032896	0.151407	6.924664603	1.482853	up
GO:0010812	negative regulation of cell-substrate adhesion	2	323	15	16775	0.032896	0.151407	6.924664603	1.482853	up
GO:0071732	cellular response to nitric oxide	2	323	15	16775	0.032896	0.151407	6.924664603	1.482853	up
GO:0050995	negative regulation of lipid catabolic process	2	323	15	16775	0.032896	0.151407	6.924664603	1.482853	up
GO:0050930	induction of positive chemotaxis	2	323	15	16775	0.032896	0.151407	6.924664603	1.482853	up
GO:0043537	negative regulation of blood vessel endothelial cell migration	2	323	15	16775	0.032896	0.151407	6.924664603	1.482853	up
GO:0015804	neutral amino acid transport	2	323	15	16775	0.032896	0.151407	6.924664603	1.482853	up
GO:0015813	L-glutamate transport	2	323	15	16775	0.032896	0.151407	6.924664603	1.482853	up
GO:0016266	O-glycan processing	4	323	63	16775	0.033087	0.151407	3.297459335	1.480346	up
GO:0010977	negative regulation of neuron projection development	3	323	37	16775	0.033934	0.151407	4.210944691	1.469367	up
GO:0006184	GTP catabolic process	10	323	269	16775	0.036028	0.151407	1.93066857	1.443363	up
GO:0003333	amino acid transmembrane transport	3	323	38	16775	0.036333	0.151407	4.100130357	1.439701	up
GO:0042417	dopamine metabolic process	2	323	16	16775	0.037126	0.151407	6.491873065	1.430321	up
GO:0019674	NAD metabolic process	2	323	16	16775	0.037126	0.151407	6.491873065	1.430321	up
GO:0030502	negative regulation of bone mineralization	2	323	16	16775	0.037126	0.151407	6.491873065	1.430321	up
GO:0060828	regulation of canonical Wnt signaling pathway	2	323	16	16775	0.037126	0.151407	6.491873065	1.430321	up
GO:0008209	androgen metabolic process	2	323	16	16775	0.037126	0.151407	6.491873065	1.430321	up
GO:0090280	positive regulation of calcium ion import	2	323	16	16775	0.037126	0.151407	6.491873065	1.430321	up
GO:0007584	response to nutrient	5	323	96	16775	0.037952	0.151407	2.70494711	1.420765	up
GO:0042704	uterine wall breakdown	1	323	2	16775	0.03814	0.151407	25.96749226	1.418619	up
GO:0002246	wound healing involved in inflammatory response	1	323	2	16775	0.03814	0.151407	25.96749226	1.418619	up
GO:0051659	maintenance of mitochondrion location	1	323	2	16775	0.03814	0.151407	25.96749226	1.418619	up
GO:0072111	cell proliferation involved in kidney development	1	323	2	16775	0.03814	0.151407	25.96749226	1.418619	up
GO:1902995	positive regulation of phospholipid efflux	1	323	2	16775	0.03814	0.151407	25.96749226	1.418619	up
GO:1902998	positive regulation of neurofibrillary tangle assembly	1	323	2	16775	0.03814	0.151407	25.96749226	1.418619	up
GO:1901201	regulation of extracellular matrix assembly	1	323	2	16775	0.03814	0.151407	25.96749226	1.418619	up
GO:2001037	positive regulation of tongue muscle cell differentiation	1	323	2	16775	0.03814	0.151407	25.96749226	1.418619	up

GO:0006212	uracil catabolic process	1	323	2	16775	0.03814	0.151407	25.96749226	1.418619	up
GO:0072180	mesonephric duct morphogenesis	1	323	2	16775	0.03814	0.151407	25.96749226	1.418619	up
GO:0032528	microvillus organization	1	323	2	16775	0.03814	0.151407	25.96749226	1.418619	up
GO:0097460	ferrous iron import into cell	1	323	2	16775	0.03814	0.151407	25.96749226	1.418619	up
GO:0061033	secretion by lung epithelial cell involved in lung growth	1	323	2	16775	0.03814	0.151407	25.96749226	1.418619	up
GO:0006233	dTDP biosynthetic process	1	323	2	16775	0.03814	0.151407	25.96749226	1.418619	up
GO:0019483	beta-alanine biosynthetic process	1	323	2	16775	0.03814	0.151407	25.96749226	1.418619	up
GO:0035947	gluconeogenesis by regulation of transcription from RNA polymera	1	323	2	16775	0.03814	0.151407	25.96749226	1.418619	up
GO:0032429	regulation of phospholipase A2 activity	1	323	2	16775	0.03814	0.151407	25.96749226	1.418619	up
GO:0034633	retinol transport	1	323	2	16775	0.03814	0.151407	25.96749226	1.418619	up
GO:0033306	phytol metabolic process	1	323	2	16775	0.03814	0.151407	25.96749226	1.418619	up
GO:1901631	positive regulation of presynaptic membrane organization	1	323	2	16775	0.03814	0.151407	25.96749226	1.418619	up
GO:0060545	positive regulation of necroptotic process	1	323	2	16775	0.03814	0.151407	25.96749226	1.418619	up
GO:0071670	smooth muscle cell chemotaxis	1	323	2	16775	0.03814	0.151407	25.96749226	1.418619	up
GO:2000660	negative regulation of interleukin-1-mediated signaling pathway	1	323	2	16775	0.03814	0.151407	25.96749226	1.418619	up
GO:0071600	otic vesicle morphogenesis	1	323	2	16775	0.03814	0.151407	25.96749226	1.418619	up
GO:0060976	coronary vasculature development	1	323	2	16775	0.03814	0.151407	25.96749226	1.418619	up
GO:0070483	detection of hypoxia	1	323	2	16775	0.03814	0.151407	25.96749226	1.418619	up
GO:1900221	regulation of beta-amyloid clearance	1	323	2	16775	0.03814	0.151407	25.96749226	1.418619	up
GO:2000058	protein ubiquitination involved in ubiquitin-dependent protein cata	1	323	2	16775	0.03814	0.151407	25.96749226	1.418619	up
GO:0072203	cell proliferation involved in metanephros development	1	323	2	16775	0.03814	0.151407	25.96749226	1.418619	up
GO:0090083	regulation of inclusion body assembly	1	323	2	16775	0.03814	0.151407	25.96749226	1.418619	up
GO:2000098	negative regulation of smooth muscle cell-matrix adhesion	1	323	2	16775	0.03814	0.151407	25.96749226	1.418619	up
GO:0010822	positive regulation of mitochondrion organization	1	323	2	16775	0.03814	0.151407	25.96749226	1.418619	up
GO:0006788	heme oxidation	1	323	2	16775	0.03814	0.151407	25.96749226	1.418619	up
GO:0072262	glomerular mesangial cell proliferation involved in metanephros	1	323	2	16775	0.03814	0.151407	25.96749226	1.418619	up
GO:0021986	habenula development	1	323	2	16775	0.03814	0.151407	25.96749226	1.418619	up
GO:0030382	sperm mitochondrion organization	1	323	2	16775	0.03814	0.151407	25.96749226	1.418619	up
GO:0045428	regulation of nitric oxide biosynthetic process	1	323	2	16775	0.03814	0.151407	25.96749226	1.418619	up
GO:0001808	negative regulation of type IV hypersensitivity	1	323	2	16775	0.03814	0.151407	25.96749226	1.418619	up
GO:0001869	negative regulation of complement activation, lectin pathway	1	323	2	16775	0.03814	0.151407	25.96749226	1.418619	up
GO:1901991	negative regulation of mitotic cell cycle phase transition	1	323	2	16775	0.03814	0.151407	25.96749226	1.418619	up
GO:2000538	positive regulation of B cell chemotaxis	1	323	2	16775	0.03814	0.151407	25.96749226	1.418619	up
GO:0008050	female courtship behavior	1	323	2	16775	0.03814	0.151407	25.96749226	1.418619	up
GO:0061502	early endosome to recycling endosome transport	1	323	2	16775	0.03814	0.151407	25.96749226	1.418619	up
GO:0090024	negative regulation of neutrophil chemotaxis	1	323	2	16775	0.03814	0.151407	25.96749226	1.418619	up
GO:0060621	negative regulation of cholesterol import	1	323	2	16775	0.03814	0.151407	25.96749226	1.418619	up
GO:2000587	regulation of platelet-derived growth factor receptor-beta signaling	1	323	2	16775	0.03814	0.151407	25.96749226	1.418619	up
GO:0060327	cytoplasmic actin-based contraction involved in cell motility	1	323	2	16775	0.03814	0.151407	25.96749226	1.418619	up
GO:0002019	regulation of renal output by angiotensin	1	323	2	16775	0.03814	0.151407	25.96749226	1.418619	up
GO:2001295	malonyl-CoA biosynthetic process	1	323	2	16775	0.03814	0.151407	25.96749226	1.418619	up
GO:0086100	endothelin receptor signaling pathway	1	323	2	16775	0.03814	0.151407	25.96749226	1.418619	up
GO:0042976	activation of Janus kinase activity	1	323	2	16775	0.03814	0.151407	25.96749226	1.418619	up
GO:0032764	negative regulation of mast cell cytokine production	1	323	2	16775	0.03814	0.151407	25.96749226	1.418619	up
GO:0038065	collagen-activated signaling pathway	1	323	2	16775	0.03814	0.151407	25.96749226	1.418619	up
GO:0038060	nitric oxide-cGMP-mediated signaling pathway	1	323	2	16775	0.03814	0.151407	25.96749226	1.418619	up
GO:0001991	ation of systemic arterial blood pressure by circulatory renin-angio	1	323	2	16775	0.03814	0.151407	25.96749226	1.418619	up
GO:0097156	fasciculation of motor neuron axon	1	323	2	16775	0.03814	0.151407	25.96749226	1.418619	up
GO:0014806	smooth muscle hyperplasia	1	323	2	16775	0.03814	0.151407	25.96749226	1.418619	up
GO:2000860	positive regulation of aldosterone secretion	1	323	2	16775	0.03814	0.151407	25.96749226	1.418619	up
GO:0060764	cell-cell signaling involved in mammary gland development	1	323	2	16775	0.03814	0.151407	25.96749226	1.418619	up
GO:0001543	ovarian follicle rupture	1	323	2	16775	0.03814	0.151407	25.96749226	1.418619	up
GO:0014873	sponse to muscle activity involved in regulation of muscle adaptati	1	323	2	16775	0.03814	0.151407	25.96749226	1.418619	up
GO:1901898	negative regulation of relaxation of cardiac muscle	1	323	2	16775	0.03814	0.151407	25.96749226	1.418619	up
GO:0034014	response to triglyceride	1	323	2	16775	0.03814	0.151407	25.96749226	1.418619	up
GO:0071871	response to epinephrine	1	323	2	16775	0.03814	0.151407	25.96749226	1.418619	up
GO:1901857	positive regulation of cellular respiration	1	323	2	16775	0.03814	0.151407	25.96749226	1.418619	up
GO:0002426	immunoglobulin production in mucosal tissue	1	323	2	16775	0.03814	0.151407	25.96749226	1.418619	up
GO:2001170	negative regulation of ATP biosynthetic process	1	323	2	16775	0.03814	0.151407	25.96749226	1.418619	up
GO:0003050	ulation of systemic arterial blood pressure by atrial natriuretic pept	1	323	2	16775	0.03814	0.151407	25.96749226	1.418619	up
GO:0072070	loop of Henle development	1	323	2	16775	0.03814	0.151407	25.96749226	1.418619	up
GO:0051562	negative regulation of mitochondrial calcium ion concentration	1	323	2	16775	0.03814	0.151407	25.96749226	1.418619	up
GO:0019442	tryptophan catabolic process to acetyl-CoA	1	323	2	16775	0.03814	0.151407	25.96749226	1.418619	up
GO:0060061	Spemann organizer formation	1	323	2	16775	0.03814	0.151407	25.96749226	1.418619	up
GO:0034505	tooth mineralization	1	323	2	16775	0.03814	0.151407	25.96749226	1.418619	up
GO:0032370	positive regulation of lipid transport	1	323	2	16775	0.03814	0.151407	25.96749226	1.418619	up
GO:0032349	positive regulation of aldosterone biosynthetic process	1	323	2	16775	0.03814	0.151407	25.96749226	1.418619	up
GO:0035441	cell migration involved in vasculogenesis	1	323	2	16775	0.03814	0.151407	25.96749226	1.418619	up
GO:0090298	negative regulation of mitochondrial DNA replication	1	323	2	16775	0.03814	0.151407	25.96749226	1.418619	up
GO:0071529	cementum mineralization	1	323	2	16775	0.03814	0.151407	25.96749226	1.418619	up
GO:0090272	negative regulation of fibroblast growth factor production	1	323	2	16775	0.03814	0.151407	25.96749226	1.418619	up
GO:0035411	catenin import into nucleus	1	323	2	16775	0.03814	0.151407	25.96749226	1.418619	up
GO:1902617	response to fluoride	1	323	2	16775	0.03814	0.151407	25.96749226	1.418619	up
GO:0046544	development of secondary male sexual characteristics	1	323	2	16775	0.03814	0.151407	25.96749226	1.418619	up
GO:0090260	negative regulation of retinal ganglion cell axon guidance	1	323	2	16775	0.03814	0.151407	25.96749226	1.418619	up
GO:0002534	cytokine production involved in inflammatory response	1	323	2	16775	0.03814	0.151407	25.96749226	1.418619	up
GO:0051917	regulation of fibrinolysis	1	323	2	16775	0.03814	0.151407	25.96749226	1.418619	up
GO:0090240	positive regulation of histone H4 acetylation	1	323	2	16775	0.03814	0.151407	25.96749226	1.418619	up
GO:0045860	positive regulation of protein kinase activity	4	323	66	16775	0.038304	0.151446	3.147574819	1.416757	up
GO:0009611	response to wounding	4	323	66	16775	0.038304	0.151446	3.147574819	1.416757	up
GO:0009314	response to radiation	3	323	39	16775	0.038817	0.15286	3.994998809	1.410983	up
GO:0009409	response to cold	3	323	39	16775	0.038817	0.15286	3.994998809	1.410983	up
GO:0007601	visual perception	8	323	201	16775	0.041155	0.159794	2.067064061	1.385581	up
GO:0060291	long-term synaptic potentiation	3	323	40	16775	0.041384	0.159794	3.895123839	1.383162	up
GO:0070527	platelet aggregation	3	323	40	16775	0.041384	0.159794	3.895123839	1.383162	up
GO:0040007	growth	3	323	40	16775	0.041384	0.159794	3.895123839	1.383162	up
GO:0045668	negative regulation of osteoblast differentiation	3	323	40	16775	0.041384	0.159794	3.895123839	1.383162	up
GO:0006182	cGMP biosynthetic process	2	323	17	16775	0.041551	0.159794	6.109998179	1.381415	up
GO:0050730	regulation of peptidyl-tyrosine phosphorylation	2	323	17	16775	0.041551	0.159794	6.109998179	1.381415	up
GO:0048596	embryonic camera-type eye morphogenesis	2	323	17	16775	0.041551	0.159794	6.109998179	1.381415	up
GO:0006826	iron ion transport	2	323	17	16775	0.041551	0.159794	6.109998179	1.381415	up
GO:0001935	endothelial cell proliferation	2	323	17	16775	0.041551	0.159794	6.109998179	1.381415	up
GO:0010035	response to inorganic substance	2	323	17	16775	0.041551	0.159794	6.109998179	1.381415	up
GO:0007413	axonal fasciculation	2	323	17	16775	0.041551	0.159794	6.109998179	1.381415	up
GO:0043433	regulation of sequence-specific DNA binding transcription factor	4	323	68	16775	0.042029	0.161315	3.054999089	1.376453	up
GO:0009887	organ morphogenesis	6	323	132	16775	0.042537	0.16295	2.360681115	1.371229	up
GO:0030162	regulation of proteolysis	3	323	41	16775	0.044036	0.168363	3.800120819	1.356193	up
GO:0007613	memory	4	323	70	16775	0.045951	0.171825	2.967713401	1.337703	up
GO:2000114	regulation of establishment of cell polarity	2	323	18	16775	0.046163	0.171825	5.770553836	1.335704	up
GO:0042310	vasoconstriction	2	323	18	16775	0.046163	0.171825	5.770553836	1.335704	up
GO:0007009	plasma membrane organization	2	323	18	16775	0.046163	0.171825	5.770553836	1.335704	up
GO:0030279	negative regulation of ossification	2	323	18	16775	0.046163	0.171825	5.770553836	1.335704	up
GO:0006790	sulfur compound metabolic process	2	323	18	16775	0.046163	0.171825	5.770553836	1.335704	up
GO:0007597	blood coagulation, intrinsic pathway	2	323	18	16775	0.046163	0.171825	5.770553836	1.335704	up
GO:0009395	phospholipid catabolic process	2	323	18	16775	0.046163	0.171825	5.770553836	1.335704	up

GO:0015711	organic anion transport	2	323	18	16775	0.046163	0.171825	5.770553836	1.335704	up
GO:0050919	negative chemotaxis	2	323	18	16775	0.046163	0.171825	5.770553836	1.335704	up
GO:0001574	ganglioside biosynthetic process	2	323	18	16775	0.046163	0.171825	5.770553836	1.335704	up
GO:0051968	positive regulation of synaptic transmission, glutamatergic	2	323	18	16775	0.046163	0.171825	5.770553836	1.335704	up
GO:0032331	negative regulation of chondrocyte differentiation	2	323	18	16775	0.046163	0.171825	5.770553836	1.335704	up
GO:0032332	positive regulation of chondrocyte differentiation	2	323	18	16775	0.046163	0.171825	5.770553836	1.335704	up
GO:0010033	response to organic substance	5	323	102	16775	0.047209	0.173244	2.545832575	1.325975	up
GO:0055114	oxidation-reduction process	21	323	736	16775	0.047359	0.173244	1.481840591	1.3246	up
GO:0060337	type I interferon signaling pathway	4	323	71	16775	0.047986	0.173244	2.925914621	1.318881	up
GO:0008015	blood circulation	3	323	43	16775	0.049586	0.173244	3.623371013	1.304642	up
GO:0051496	positive regulation of stress fiber assembly	3	323	43	16775	0.049586	0.173244	3.623371013	1.304642	up
GO:0045599	negative regulation of fat cell differentiation	3	323	43	16775	0.049586	0.173244	3.623371013	1.304642	up
GO:0006334	nucleosome assembly	10	171	134	16775	1.14E-06	0.001406	7.320851881	5.941632	down
GO:0032776	DNA methylation on cytosine	5	171	34	16775	2.27E-05	0.013971	14.42638459	4.643293	down
GO:0008285	negative regulation of cell proliferation	15	171	431	16775	3.62E-05	0.014813	3.41413278	4.441773	down
GO:0000183	chromatin silencing at rDNA	5	171	42	16775	6.51E-05	0.018958	11.67850181	4.186574	down
GO:0006325	chromatin organization	11	171	261	16775	7.71E-05	0.018958	4.134458112	4.112782	down
GO:0035556	intracellular signal transduction	15	171	484	16775	0.000133	0.026301	3.040271132	3.877162	down
GO:0060968	regulation of gene silencing	3	171	11	16775	0.000162	0.026301	26.75438596	3.791252	down
GO:0008284	positive regulation of cell proliferation	15	171	503	16775	0.000202	0.026301	2.925429877	3.695368	down
GO:0071456	cellular response to hypoxia	7	171	118	16775	0.000206	0.026301	5.819456834	3.685346	down
GO:0046888	negative regulation of hormone secretion	3	171	12	16775	0.000214	0.026301	24.5248538	3.669576	down
GO:0000079	gulation of cyclin-dependent protein serine/threonine kinase activi	5	171	57	16775	0.000283	0.030723	8.60521186	3.547911	down
GO:0006335	DNA replication-dependent nucleosome assembly	4	171	32	16775	0.0003	0.030723	12.2624269	3.522909	down
GO:0007264	small GTPase mediated signal transduction	18	171	731	16775	0.000489	0.046195	2.415580675	3.311009	down
GO:0051290	protein heterotetramerization	4	171	37	16775	0.00053	0.046486	10.60534218	3.276102	down
GO:0071548	response to dexamethasone	2	171	4	16775	0.000612	0.050108	49.0497076	3.213555	down
GO:0060326	cell chemotaxis	5	171	75	16775	0.001007	0.077387	6.539961014	2.996761	down
GO:0002690	positive regulation of leukocyte chemotaxis	3	171	21	16775	0.001209	0.08743	14.01420217	2.917443	down
GO:0046033	AMP metabolic process	2	171	6	16775	0.001508	0.097576	32.69980507	2.821457	down
GO:0046039	GTP metabolic process	2	171	6	16775	0.001508	0.097576	32.69980507	2.821457	down
GO:0007399	nervous system development	13	171	498	16775	0.001806	0.111	2.560828108	2.743198	down
GO:0045630	positive regulation of T-helper 2 cell differentiation	2	171	7	16775	0.002098	0.122768	28.02840434	2.678247	down
GO:0048010	vascular endothelial growth factor receptor signaling pathway	9	171	281	16775	0.002401	0.131489	3.141974152	2.619643	down
GO:0045814	negative regulation of gene expression, epigenetic	5	171	94	16775	0.002745	0.131489	5.218054	2.561522	down
GO:0042102	positive regulation of T cell proliferation	4	171	58	16775	0.002882	0.131489	6.765476911	2.540301	down
GO:0006820	anion transport	3	171	29	16775	0.00313	0.131489	10.14821537	2.504462	down
GO:0001666	response to hypoxia	7	171	190	16775	0.003375	0.131489	3.614188981	2.471723	down
GO:2000177	regulation of neural precursor cell proliferation	2	171	9	16775	0.003548	0.131489	21.79987005	2.449992	down
GO:0019511	peptidyl-proline hydroxylation	2	171	9	16775	0.003548	0.131489	21.79987005	2.449992	down
GO:0043653	mitochondrial fragmentation involved in apoptotic process	2	171	9	16775	0.003548	0.131489	21.79987005	2.449992	down
GO:0042060	wound healing	5	171	103	16775	0.004069	0.131489	4.762107534	2.390555	down
GO:0051930	regulation of sensory perception of pain	3	171	32	16775	0.004155	0.131489	9.196820175	2.381449	down
GO:0045214	sarcomere organization	3	171	33	16775	0.004536	0.131489	8.918128655	2.343286	down
GO:0040029	regulation of gene expression, epigenetic	5	171	108	16775	0.004975	0.131489	4.541639593	2.30322	down
GO:0042511	positive regulation of tyrosine phosphorylation of Stat1 protein	2	171	11	16775	0.005349	0.131489	17.83625731	2.27175	down
GO:0070098	chemokine-mediated signaling pathway	4	171	69	16775	0.005386	0.131489	5.686922621	2.268697	down
GO:0000082	G1/S transition of mitotic cell cycle	6	171	157	16775	0.005476	0.131489	3.749022237	2.261523	down
GO:0006260	DNA replication	6	171	157	16775	0.005476	0.131489	3.749022237	2.261523	down
GO:0035249	synaptic transmission, glutamatergic	3	171	37	16775	0.006271	0.131489	7.954006638	2.202644	down
GO:0032700	negative regulation of interleukin-17 production	2	171	12	16775	0.006376	0.131489	16.34990253	2.195475	down
GO:0007171	ctivation of transmembrane receptor protein tyrosine kinase activit	2	171	12	16775	0.006376	0.131489	16.34990253	2.195475	down
GO:0055008	cardiac muscle tissue morphogenesis	2	171	12	16775	0.006376	0.131489	16.34990253	2.195475	down
GO:0000165	MAPK cascade	8	171	271	16775	0.006704	0.131489	2.895923696	2.17365	down
GO:0051402	neuron apoptotic process	3	171	38	16775	0.006758	0.131489	7.744690674	2.170151	down
GO:0033138	positive regulation of peptidyl-serine phosphorylation	4	171	75	16775	0.007225	0.131489	5.231968811	2.141132	down
GO:0045651	positive regulation of macrophage differentiation	2	171	13	16775	0.007485	0.131489	15.09221772	2.125827	down
GO:0043627	response to estrogen	4	171	77	16775	0.00792	0.131489	5.096073517	2.101276	down
GO:0030307	positive regulation of cell growth	4	171	77	16775	0.00792	0.131489	5.096073517	2.101276	down
GO:0002230	positive regulation of defense response to virus by host	5	171	123	16775	0.008541	0.131489	3.987781106	2.068469	down
GO:0007076	mitotic chromosome condensation	2	171	14	16775	0.008674	0.131489	14.01420217	2.061781	down
GO:0032026	response to magnesium ion	2	171	14	16775	0.008674	0.131489	14.01420217	2.061781	down
GO:0086004	regulation of cardiac muscle cell contraction	2	171	14	16775	0.008674	0.131489	14.01420217	2.061781	down
GO:0014032	neural crest cell development	2	171	15	16775	0.009942	0.131489	13.07992203	2.002532	down
GO:0060347	heart trabecula formation	2	171	15	16775	0.009942	0.131489	13.07992203	2.002532	down
GO:2000179	positive regulation of neural precursor cell proliferation	2	171	15	16775	0.009942	0.131489	13.07992203	2.002532	down
GO:0071850	mitotic cell cycle arrest	2	171	15	16775	0.009942	0.131489	13.07992203	2.002532	down
GO:0050930	induction of positive chemotaxis	2	171	15	16775	0.009942	0.131489	13.07992203	2.002532	down
GO:1903403	negative regulation of renal phosphate excretion	1	171	1	16775	0.010194	0.131489	98.0994152	1.991666	down
GO:1902749	regulation of cell cycle G2/M phase transition	1	171	1	16775	0.010194	0.131489	98.0994152	1.991666	down
GO:1901676	positive regulation of histone H3-K27 acetylation	1	171	1	16775	0.010194	0.131489	98.0994152	1.991666	down
GO:0006679	glucosylceramide biosynthetic process	1	171	1	16775	0.010194	0.131489	98.0994152	1.991666	down
GO:0042313	protein kinase C deactivation	1	171	1	16775	0.010194	0.131489	98.0994152	1.991666	down
GO:0035712	T-helper 2 cell activation	1	171	1	16775	0.010194	0.131489	98.0994152	1.991666	down
GO:0035713	response to nitrogen dioxide	1	171	1	16775	0.010194	0.131489	98.0994152	1.991666	down
GO:0035714	cellular response to nitrogen dioxide	1	171	1	16775	0.010194	0.131489	98.0994152	1.991666	down
GO:0035709	memory T cell activation	1	171	1	16775	0.010194	0.131489	98.0994152	1.991666	down
GO:0031583	spholipase D-activating G-protein coupled receptor signaling path	1	171	1	16775	0.010194	0.131489	98.0994152	1.991666	down
GO:0001993	ation of systemic arterial blood pressure by norepinephrine-epinep	1	171	1	16775	0.010194	0.131489	98.0994152	1.991666	down
GO:0014861	ulation of skeletal muscle contraction via regulation of action poter	1	171	1	16775	0.010194	0.131489	98.0994152	1.991666	down
GO:0035783	CD4-positive, alpha-beta T cell costimulation	1	171	1	16775	0.010194	0.131489	98.0994152	1.991666	down
GO:1902975	mitotic DNA replication initiation	1	171	1	16775	0.010194	0.131489	98.0994152	1.991666	down
GO:0060721	regulation of spongiotrophoblast cell proliferation	1	171	1	16775	0.010194	0.131489	98.0994152	1.991666	down
GO:2000688	ositive regulation of rubidium ion transmembrane transporter activ	1	171	1	16775	0.010194	0.131489	98.0994152	1.991666	down
GO:2000682	positive regulation of rubidium ion transport	1	171	1	16775	0.010194	0.131489	98.0994152	1.991666	down
GO:0071867	response to monoamine	1	171	1	16775	0.010194	0.131489	98.0994152	1.991666	down
GO:0045091	f single stranded viral RNA replication via double stranded DNA i	1	171	1	16775	0.010194	0.131489	98.0994152	1.991666	down
GO:0002371	dendritic cell cytokine production	1	171	1	16775	0.010194	0.131489	98.0994152	1.991666	down
GO:0072213	metanephric capsule development	1	171	1	16775	0.010194	0.131489	98.0994152	1.991666	down
GO:0002335	mature B cell differentiation	1	171	1	16775	0.010194	0.131489	98.0994152	1.991666	down
GO:0051545	negative regulation of elastin biosynthetic process	1	171	1	16775	0.010194	0.131489	98.0994152	1.991666	down
GO:1900281	ositive regulation of CD4-positive, alpha-beta T cell costimulatio	1	171	1	16775	0.010194	0.131489	98.0994152	1.991666	down
GO:0060678	tomous subdivision of terminal units involved in ureteric bud bran	1	171	1	16775	0.010194	0.131489	98.0994152	1.991666	down
GO:0030844	positive regulation of intermediate filament depolymerization	1	171	1	16775	0.010194	0.131489	98.0994152	1.991666	down
GO:0072432	response to G1 DNA damage checkpoint signaling	1	171	1	16775	0.010194	0.131489	98.0994152	1.991666	down
GO:0003059	positive regulation of the force of heart contraction by epinephrine	1	171	1	16775	0.010194	0.131489	98.0994152	1.991666	down
GO:0072076	nephrogenic mesenchyme development	1	171	1	16775	0.010194	0.131489	98.0994152	1.991666	down
GO:0072267	metanephric capsule specification	1	171	1	16775	0.010194	0.131489	98.0994152	1.991666	down
GO:0097326	melanocyte adhesion	1	171	1	16775	0.010194	0.131489	98.0994152	1.991666	down
GO:0090218	positive regulation of lipid kinase activity	1	171	1	16775	0.010194	0.131489	98.0994152	1.991666	down
GO:0046521	sphingoid catabolic process	1	171	1	16775	0.010194	0.131489	98.0994152	1.991666	down
GO:0021686	cerebellar granular layer maturation	1	171	1	16775	0.010194	0.131489	98.0994152	1.991666	down
GO:0060419	heart growth	1	171	1	16775	0.010194	0.131489	98.0994152	1.991666	down
GO:0003421	growth plate cartilage axis specification	1	171	1	16775	0.010194	0.131489	98.0994152	1.991666	down
GO:0070662	mast cell proliferation	1	171	1	16775	0.010194	0.131489	98.0994152	1.991666	down

GO:2000572	e regulation of interleukin-4-dependent isotype switching to IgE is	1	171	1	16775	0.010194	0.131489	98.0994152	1.991666	down
GO:0043056	forward locomotion	1	171	1	16775	0.010194	0.131489	98.0994152	1.991666	down
GO:0008543	fibroblast growth factor receptor signaling pathway	8	171	292	16775	0.010271	0.131489	2.687655211	1.98839	down
GO:0051260	protein homooligomerization	6	171	181	16775	0.010683	0.132659	3.251914316	1.97129	down
GO:0051897	positive regulation of protein kinase B signaling	4	171	84	16775	0.010692	0.132659	4.671400724	1.970956	down
GO:0010976	positive regulation of neuron projection development	4	171	84	16775	0.010692	0.132659	4.671400724	1.970956	down
GO:0045595	regulation of cell differentiation	3	171	45	16775	0.010794	0.132659	6.539961014	1.966814	down
GO:0070528	protein kinase C signaling	2	171	16	16775	0.011287	0.137339	12.2624269	1.947436	down
GO:0050731	positive regulation of peptidyl-tyrosine phosphorylation	4	171	86	16775	0.011585	0.139586	4.562763498	1.936109	down
GO:0007265	Ras protein signal transduction	7	171	243	16775	0.01241	0.144601	2.82590908	1.906244	down
GO:0071380	cellular response to prostaglandin E stimulus	2	171	17	16775	0.012707	0.144601	11.54110767	1.895972	down
GO:0007596	blood coagulation	11	171	495	16775	0.012728	0.144601	2.179987005	1.895234	down
GO:0031532	actin cytoskeleton reorganization	3	171	48	16775	0.012868	0.144601	6.13121345	1.890473	down
GO:0043410	positive regulation of MAPK cascade	4	171	89	16775	0.013012	0.144601	4.408962481	1.885643	down
GO:0006954	inflammatory response	9	171	367	16775	0.0131	0.144601	2.40570773	1.882717	down
GO:0000278	mitotic cell cycle	10	171	435	16775	0.013872	0.144601	2.25515897	1.857856	down
GO:0007420	brain development	7	171	249	16775	0.01403	0.144601	2.757814885	1.852943	down
GO:0032753	positive regulation of interleukin-4 production	2	171	18	16775	0.0142	0.144601	10.89993502	1.847711	down
GO:0021591	ventricular system development	2	171	18	16775	0.0142	0.144601	10.89993502	1.847711	down
GO:0070373	negative regulation of ERK1 and ERK2 cascade	3	171	51	16775	0.015155	0.144601	5.770553836	1.819442	down
GO:0010001	glial cell differentiation	2	171	19	16775	0.015765	0.144601	10.32625423	1.802296	down
GO:0010039	response to iron ion	2	171	19	16775	0.015765	0.144601	10.32625423	1.802296	down
GO:0031954	positive regulation of protein autophosphorylation	2	171	19	16775	0.015765	0.144601	10.32625423	1.802296	down
GO:0045785	positive regulation of cell adhesion	3	171	52	16775	0.015965	0.144601	5.659581646	1.796834	down
GO:0007050	cell cycle arrest	5	171	144	16775	0.016057	0.144601	3.406229695	1.794345	down
GO:0071385	cellular response to glucocorticoid stimulus	2	171	20	16775	0.017401	0.144601	9.80994152	1.759425	down
GO:0048666	neuron development	3	171	54	16775	0.017656	0.144601	5.449967511	1.753098	down
GO:0042730	fibrinolysis	2	171	21	16775	0.019105	0.144601	9.342801448	1.718843	down
GO:0045737	ve regulation of cyclin-dependent protein serine/threonine kinase a	2	171	21	16775	0.019105	0.144601	9.342801448	1.718843	down
GO:0008156	negative regulation of DNA replication	2	171	21	16775	0.019105	0.144601	9.342801448	1.718843	down
GO:0046697	decidualization	2	171	21	16775	0.019105	0.144601	9.342801448	1.718843	down
GO:0046939	nucleotide phosphorylation	2	171	21	16775	0.019105	0.144601	9.342801448	1.718843	down
GO:0014068	positive regulation of phosphatidylinositol 3-kinase signaling	3	171	56	16775	0.019444	0.144601	5.255325815	1.711213	down
GO:2000116	regulation of cysteine-type endopeptidase activity	1	171	2	16775	0.020284	0.144601	49.0497076	1.692843	down
GO:0046031	ADP metabolic process	1	171	2	16775	0.020284	0.144601	49.0497076	1.692843	down
GO:0039535	regulation of RIG-I signaling pathway	1	171	2	16775	0.020284	0.144601	49.0497076	1.692843	down
GO:0014013	regulation of gliogenesis	1	171	2	16775	0.020284	0.144601	49.0497076	1.692843	down
GO:0014010	Schwann cell proliferation	1	171	2	16775	0.020284	0.144601	49.0497076	1.692843	down
GO:0036018	cellular response to erythropoietin	1	171	2	16775	0.020284	0.144601	49.0497076	1.692843	down
GO:0043624	cellular protein complex disassembly	1	171	2	16775	0.020284	0.144601	49.0497076	1.692843	down
GO:1903025	n of RNA polymerase II regulatory region sequence-specific DNA	1	171	2	16775	0.020284	0.144601	49.0497076	1.692843	down
GO:1901098	positive regulation of autophagic vacuole maturation	1	171	2	16775	0.020284	0.144601	49.0497076	1.692843	down
GO:0060168	positive regulation of adenosine receptor signaling pathway	1	171	2	16775	0.020284	0.144601	49.0497076	1.692843	down
GO:0032762	mast cell cytokine production	1	171	2	16775	0.020284	0.144601	49.0497076	1.692843	down
GO:0032414	positive regulation of ion transmembrane transporter activity	1	171	2	16775	0.020284	0.144601	49.0497076	1.692843	down
GO:0009450	gamma-aminobutyric acid catabolic process	1	171	2	16775	0.020284	0.144601	49.0497076	1.692843	down
GO:0006188	IMP biosynthetic process	1	171	2	16775	0.020284	0.144601	49.0497076	1.692843	down
GO:0006196	AMP catabolic process	1	171	2	16775	0.020284	0.144601	49.0497076	1.692843	down
GO:0002638	negative regulation of immunoglobulin production	1	171	2	16775	0.020284	0.144601	49.0497076	1.692843	down
GO:0002891	positive regulation of immunoglobulin mediated immune response	1	171	2	16775	0.020284	0.144601	49.0497076	1.692843	down
GO:0070101	positive regulation of chemokine-mediated signaling pathway	1	171	2	16775	0.020284	0.144601	49.0497076	1.692843	down
GO:2000664	positive regulation of interleukin-5 secretion	1	171	2	16775	0.020284	0.144601	49.0497076	1.692843	down
GO:0043179	rhythmic excitation	1	171	2	16775	0.020284	0.144601	49.0497076	1.692843	down
GO:0030821	negative regulation of cAMP catabolic process	1	171	2	16775	0.020284	0.144601	49.0497076	1.692843	down
GO:0038111	interleukin-7-mediated signaling pathway	1	171	2	16775	0.020284	0.144601	49.0497076	1.692843	down
GO:0038109	Kit signaling pathway	1	171	2	16775	0.020284	0.144601	49.0497076	1.692843	down
GO:0060437	lung growth	1	171	2	16775	0.020284	0.144601	49.0497076	1.692843	down
GO:0007620	copulation	1	171	2	16775	0.020284	0.144601	49.0497076	1.692843	down
GO:0002380	immunoglobulin secretion involved in immune response	1	171	2	16775	0.020284	0.144601	49.0497076	1.692843	down
GO:0090076	relaxation of skeletal muscle	1	171	2	16775	0.020284	0.144601	49.0497076	1.692843	down
GO:0051126	negative regulation of actin nucleation	1	171	2	16775	0.020284	0.144601	49.0497076	1.692843	down
GO:0021941	egative regulation of cerebellar granule cell precursor proliferatio	1	171	2	16775	0.020284	0.144601	49.0497076	1.692843	down
GO:0021933	radial glia guided migration of cerebellar granule cell	1	171	2	16775	0.020284	0.144601	49.0497076	1.692843	down
GO:1990144	intrinsic apoptotic signaling pathway in response to hypoxia	1	171	2	16775	0.020284	0.144601	49.0497076	1.692843	down
GO:0097324	melanocyte migration	1	171	2	16775	0.020284	0.144601	49.0497076	1.692843	down
GO:0072268	pattern specification involved in metanephros development	1	171	2	16775	0.020284	0.144601	49.0497076	1.692843	down
GO:0038162	erythropoietin-mediated signaling pathway	1	171	2	16775	0.020284	0.144601	49.0497076	1.692843	down
GO:0032796	uropod organization	1	171	2	16775	0.020284	0.144601	49.0497076	1.692843	down
GO:0071954	chemokine (C-C motif) ligand 11 production	1	171	2	16775	0.020284	0.144601	49.0497076	1.692843	down
GO:1901989	positive regulation of cell cycle phase transition	1	171	2	16775	0.020284	0.144601	49.0497076	1.692843	down
GO:2000525	positive regulation of T cell costimulation	1	171	2	16775	0.020284	0.144601	49.0497076	1.692843	down
GO:0061502	early endosome to recycling endosome transport	1	171	2	16775	0.020284	0.144601	49.0497076	1.692843	down
GO:0055002	striated muscle cell development	1	171	2	16775	0.020284	0.144601	49.0497076	1.692843	down
GO:2000570	positive regulation of T-helper 2 cell activation	1	171	2	16775	0.020284	0.144601	49.0497076	1.692843	down
GO:0002327	immature B cell differentiation	1	171	2	16775	0.020284	0.144601	49.0497076	1.692843	down
GO:2000568	positive regulation of memory T cell activation	1	171	2	16775	0.020284	0.144601	49.0497076	1.692843	down
GO:1990502	dense core granule maturation	1	171	2	16775	0.020284	0.144601	49.0497076	1.692843	down
GO:0051726	regulation of cell cycle	5	171	153	16775	0.020312	0.144601	3.205863242	1.692237	down
GO:0019722	calcium-mediated signaling	3	171	57	16775	0.020374	0.144601	5.163127116	1.690923	down
GO:0043406	positive regulation of MAP kinase activity	3	171	57	16775	0.020374	0.144601	5.163127116	1.690923	down
GO:0007565	female pregnancy	4	171	102	16775	0.020472	0.144601	3.84703589	1.68883	down
GO:0006955	immune response	9	171	397	16775	0.020734	0.144959	2.223916214	1.68331	down
GO:0045987	positive regulation of smooth muscle contraction	2	171	22	16775	0.020877	0.144959	8.918128655	1.680332	down
GO:0090023	positive regulation of neutrophil chemotaxis	2	171	22	16775	0.020877	0.144959	8.918128655	1.680332	down
GO:2000378	negative regulation of reactive oxygen species metabolic process	2	171	23	16775	0.022714	0.147054	8.530383931	1.643703	down
GO:0071345	cellular response to cytokine stimulus	2	171	23	16775	0.022714	0.147054	8.530383931	1.643703	down
GO:0010765	positive regulation of sodium ion transport	2	171	23	16775	0.022714	0.147054	8.530383931	1.643703	down
GO:0007411	axon guidance	11	171	543	16775	0.023516	0.147054	1.987280971	1.628628	down
GO:0016311	dephosphorylation	6	171	218	16775	0.024418	0.147054	2.699983905	1.612281	down
GO:0007165	signal transduction	31	171	2125	16775	0.024515	0.147054	1.431097351	1.610576	down
GO:0030833	regulation of actin filament polymerization	2	171	24	16775	0.024616	0.147054	8.174951267	1.608791	down
GO:0045766	positive regulation of angiogenesis	4	171	108	16775	0.024646	0.147054	3.633311674	1.608255	down
GO:0030335	positive regulation of cell migration	5	171	161	16775	0.024665	0.147054	3.04656569	1.607926	down
GO:0030593	neutrophil chemotaxis	3	171	63	16775	0.026461	0.147054	4.671400724	1.577397	down
GO:0051899	membrane depolarization	2	171	25	16775	0.026579	0.147054	7.847953216	1.575454	down
GO:0071222	cellular response to lipopolysaccharide	4	171	112	16775	0.027694	0.147054	3.503550543	1.557619	down
GO:2000352	negative regulation of endothelial cell apoptotic process	2	171	26	16775	0.028605	0.147054	7.546108862	1.543563	down
GO:0051453	regulation of intracellular pH	2	171	26	16775	0.028605	0.147054	7.546108862	1.543563	down
GO:0032689	negative regulation of interferon-gamma production	2	171	26	16775	0.028605	0.147054	7.546108862	1.543563	down
GO:0001954	positive regulation of cell-matrix adhesion	2	171	26	16775	0.028605	0.147054	7.546108862	1.543563	down
GO:0010800	positive regulation of peptidyl-threonine phosphorylation	2	171	26	16775	0.028605	0.147054	7.546108862	1.543563	down
GO:0046580	negative regulation of Ras protein signal transduction	2	171	26	16775	0.028605	0.147054	7.546108862	1.543563	down
GO:0031398	positive regulation of protein ubiquitination	3	171	66	16775	0.029829	0.147054	4.459064327	1.525355	down
GO:0005989	lactose biosynthetic process	1	171	3	16775	0.030272	0.147054	32.69980507	1.518954	down
GO:1903659	regulation of complement-dependent cytotoxicity	1	171	3	16775	0.030272	0.147054	32.69980507	1.518954	down

GO:0097411	hypoxia-inducible factor-1alpha signaling pathway	1	171	3	16775	0.030272	0.147054	32.69980507	1.518954	down
GO:0002215	defense response to nematode	1	171	3	16775	0.030272	0.147054	32.69980507	1.518954	down
GO:0071279	cellular response to cobalt ion	1	171	3	16775	0.030272	0.147054	32.69980507	1.518954	down
GO:0060374	mast cell differentiation	1	171	3	16775	0.030272	0.147054	32.69980507	1.518954	down
GO:1901492	positive regulation of lymphangiogenesis	1	171	3	16775	0.030272	0.147054	32.69980507	1.518954	down
GO:0060379	cardiac muscle cell myoblast differentiation	1	171	3	16775	0.030272	0.147054	32.69980507	1.518954	down
GO:0060564	egative regulation of mitotic anaphase-promoting complex activit	1	171	3	16775	0.030272	0.147054	32.69980507	1.518954	down
GO:0060585	positive regulation of prostaglandin-endoperoxide synthase activity	1	171	3	16775	0.030272	0.147054	32.69980507	1.518954	down
GO:0002025	orepinephrine-epinephrine involved in regulation of systemic arter	1	171	3	16775	0.030272	0.147054	32.69980507	1.518954	down
GO:0070141	response to UV-A	1	171	3	16775	0.030272	0.147054	32.69980507	1.518954	down
GO:0002032	ization of G-protein coupled receptor protein signaling pathway by	1	171	3	16775	0.030272	0.147054	32.69980507	1.518954	down
GO:0002086	diaphragm contraction	1	171	3	16775	0.030272	0.147054	32.69980507	1.518954	down
GO:0032946	positive regulation of mononuclear cell proliferation	1	171	3	16775	0.030272	0.147054	32.69980507	1.518954	down
GO:0045837	negative regulation of membrane potential	1	171	3	16775	0.030272	0.147054	32.69980507	1.518954	down
GO:0021801	cerebral cortex radial glia guided migration	1	171	3	16775	0.030272	0.147054	32.69980507	1.518954	down
GO:0031652	positive regulation of heat generation	1	171	3	16775	0.030272	0.147054	32.69980507	1.518954	down
GO:0042524	negative regulation of tyrosine phosphorylation of Stat5 protein	1	171	3	16775	0.030272	0.147054	32.69980507	1.518954	down
GO:0031649	heat generation	1	171	3	16775	0.030272	0.147054	32.69980507	1.518954	down
GO:0009449	gamma-aminobutyric acid biosynthetic process	1	171	3	16775	0.030272	0.147054	32.69980507	1.518954	down
GO:0009448	gamma-aminobutyric acid metabolic process	1	171	3	16775	0.030272	0.147054	32.69980507	1.518954	down
GO:0044691	tooth eruption	1	171	3	16775	0.030272	0.147054	32.69980507	1.518954	down
GO:0031339	negative regulation of vesicle fusion	1	171	3	16775	0.030272	0.147054	32.69980507	1.518954	down
GO:0021781	glial cell fate commitment	1	171	3	16775	0.030272	0.147054	32.69980507	1.518954	down
GO:0030241	skeletal muscle myosin thick filament assembly	1	171	3	16775	0.030272	0.147054	32.69980507	1.518954	down
GO:0045321	leukocyte activation	1	171	3	16775	0.030272	0.147054	32.69980507	1.518954	down
GO:0032651	regulation of interleukin-1 beta production	1	171	3	16775	0.030272	0.147054	32.69980507	1.518954	down
GO:1902074	response to salt	1	171	3	16775	0.030272	0.147054	32.69980507	1.518954	down
GO:0042461	photoreceptor cell development	1	171	3	16775	0.030272	0.147054	32.69980507	1.518954	down
GO:0032275	luteinizing hormone secretion	1	171	3	16775	0.030272	0.147054	32.69980507	1.518954	down
GO:0060544	regulation of necroptotic process	1	171	3	16775	0.030272	0.147054	32.69980507	1.518954	down
GO:0034447	very-low-density lipoprotein particle clearance	1	171	3	16775	0.030272	0.147054	32.69980507	1.518954	down
GO:0034436	glycoprotein transport	1	171	3	16775	0.030272	0.147054	32.69980507	1.518954	down
GO:0046666	retinal cell programmed cell death	1	171	3	16775	0.030272	0.147054	32.69980507	1.518954	down
GO:1902916	positive regulation of protein polyubiquitination	1	171	3	16775	0.030272	0.147054	32.69980507	1.518954	down
GO:0048858	cell projection morphogenesis	1	171	3	16775	0.030272	0.147054	32.69980507	1.518954	down
GO:0045590	negative regulation of regulatory T cell differentiation	1	171	3	16775	0.030272	0.147054	32.69980507	1.518954	down
GO:0035330	regulation of hippo signaling	1	171	3	16775	0.030272	0.147054	32.69980507	1.518954	down
GO:0048871	multicellular organismal homeostasis	1	171	3	16775	0.030272	0.147054	32.69980507	1.518954	down
GO:0086001	cardiac muscle cell action potential	1	171	3	16775	0.030272	0.147054	32.69980507	1.518954	down
GO:0042663	regulation of endodermal cell fate specification	1	171	3	16775	0.030272	0.147054	32.69980507	1.518954	down
GO:0051386	regulation of neurotrophin TRK receptor signaling pathway	1	171	3	16775	0.030272	0.147054	32.69980507	1.518954	down
GO:1901165	positive regulation of trophoblast cell migration	1	171	3	16775	0.030272	0.147054	32.69980507	1.518954	down
GO:0070837	dehydroascorbic acid transport	1	171	3	16775	0.030272	0.147054	32.69980507	1.518954	down
GO:0043243	positive regulation of protein complex disassembly	1	171	3	16775	0.030272	0.147054	32.69980507	1.518954	down
GO:0038093	Fc receptor signaling pathway	1	171	3	16775	0.030272	0.147054	32.69980507	1.518954	down
GO:0010573	vascular endothelial growth factor production	1	171	3	16775	0.030272	0.147054	32.69980507	1.518954	down
GO:0030185	nitric oxide transport	1	171	3	16775	0.030272	0.147054	32.69980507	1.518954	down
GO:0045626	negative regulation of T-helper 1 cell differentiation	1	171	3	16775	0.030272	0.147054	32.69980507	1.518954	down
GO:0048769	sarcomerogenesis	1	171	3	16775	0.030272	0.147054	32.69980507	1.518954	down
GO:0060420	regulation of heart growth	1	171	3	16775	0.030272	0.147054	32.69980507	1.518954	down
GO:0000320	re-entry into mitotic cell cycle	1	171	3	16775	0.030272	0.147054	32.69980507	1.518954	down
GO:0003408	optic cup formation involved in camera-type eye development	1	171	3	16775	0.030272	0.147054	32.69980507	1.518954	down
GO:0070424	of nucleotide-binding oligomerization domain containing signalin	1	171	3	16775	0.030272	0.147054	32.69980507	1.518954	down
GO:0035694	mitochondrial protein catabolic process	1	171	3	16775	0.030272	0.147054	32.69980507	1.518954	down
GO:0034121	regulation of toll-like receptor signaling pathway	1	171	3	16775	0.030272	0.147054	32.69980507	1.518954	down
GO:0060135	maternal process involved in female pregnancy	2	171	27	16775	0.03069	0.147912	7.266623348	1.513009	down
GO:0005978	glycogen biosynthetic process	2	171	27	16775	0.03069	0.147912	7.266623348	1.513009	down
GO:0071347	cellular response to interleukin-1	3	171	68	16775	0.032195	0.154561	4.327915377	1.492211	down
GO:0007616	long-term memory	2	171	28	16775	0.032833	0.155675	7.007101086	1.483692	down
GO:0006270	DNA replication initiation	2	171	28	16775	0.032833	0.155675	7.007101086	1.483692	down
GO:0090200	positive regulation of release of cytochrome c from mitochondria	2	171	28	16775	0.032833	0.155675	7.007101086	1.483692	down
GO:0000186	activation of MAPKK activity	6	171	234	16775	0.032934	0.155675	2.515369621	1.48236	down
GO:0042149	cellular response to glucose starvation	2	171	30	16775	0.037289	0.161293	6.539961014	1.428421	down
GO:0008286	insulin receptor signaling pathway	7	171	306	16775	0.037575	0.161293	2.244104269	1.425103	down
GO:0048015	phosphatidylinositol-mediated signaling	4	171	124	16775	0.038137	0.161293	3.164497265	1.418653	down
GO:0042517	positive regulation of tyrosine phosphorylation of Stat3 protein	2	171	31	16775	0.039599	0.161293	6.328994529	1.402317	down
GO:0045776	negative regulation of blood pressure	2	171	31	16775	0.039599	0.161293	6.328994529	1.402317	down
GO:0033574	response to testosterone	2	171	31	16775	0.039599	0.161293	6.328994529	1.402317	down
GO:0051409	response to nitrosative stress	1	171	4	16775	0.040159	0.161293	24.5248538	1.396214	down
GO:0072108	on of mesenchymal to epithelial transition involved in metanephro	1	171	4	16775	0.040159	0.161293	24.5248538	1.396214	down
GO:0038026	reelin-mediated signaling pathway	1	171	4	16775	0.040159	0.161293	24.5248538	1.396214	down
GO:0006670	sphingosine metabolic process	1	171	4	16775	0.040159	0.161293	24.5248538	1.396214	down
GO:0003161	cardiac conduction system development	1	171	4	16775	0.040159	0.161293	24.5248538	1.396214	down
GO:0042520	positive regulation of tyrosine phosphorylation of Stat4 protein	1	171	4	16775	0.040159	0.161293	24.5248538	1.396214	down
GO:0043372	ositive regulation of CD4-positive, alpha-beta T cell differentiatio	1	171	4	16775	0.040159	0.161293	24.5248538	1.396214	down
GO:0035995	detection of muscle stretch	1	171	4	16775	0.040159	0.161293	24.5248538	1.396214	down
GO:0014826	vein smooth muscle contraction	1	171	4	16775	0.040159	0.161293	24.5248538	1.396214	down
GO:0034696	response to prostaglandin F	1	171	4	16775	0.040159	0.161293	24.5248538	1.396214	down
GO:0032269	negative regulation of cellular protein metabolic process	1	171	4	16775	0.040159	0.161293	24.5248538	1.396214	down
GO:1901897	regulation of relaxation of cardiac muscle	1	171	4	16775	0.040159	0.161293	24.5248538	1.396214	down
GO:0007089	traversing start control point of mitotic cell cycle	1	171	4	16775	0.040159	0.161293	24.5248538	1.396214	down
GO:2000667	positive regulation of interleukin-13 secretion	1	171	4	16775	0.040159	0.161293	24.5248538	1.396214	down
GO:0034261	negative regulation of Ras GTPase activity	1	171	4	16775	0.040159	0.161293	24.5248538	1.396214	down
GO:0048861	leukemia inhibitory factor signaling pathway	1	171	4	16775	0.040159	0.161293	24.5248538	1.396214	down
GO:0051771	negative regulation of nitric-oxide synthase biosynthetic process	1	171	4	16775	0.040159	0.161293	24.5248538	1.396214	down
GO:0072210	metanephric nephron development	1	171	4	16775	0.040159	0.161293	24.5248538	1.396214	down
GO:2000021	regulation of ion homeostasis	1	171	4	16775	0.040159	0.161293	24.5248538	1.396214	down
GO:0061589	calcium activated phosphatidylserine scrambling	1	171	4	16775	0.040159	0.161293	24.5248538	1.396214	down
GO:0071389	cellular response to mineralocorticoid stimulus	1	171	4	16775	0.040159	0.161293	24.5248538	1.396214	down
GO:0006772	thiamine metabolic process	1	171	4	16775	0.040159	0.161293	24.5248538	1.396214	down
GO:0051984	positive regulation of chromosome segregation	1	171	4	16775	0.040159	0.161293	24.5248538	1.396214	down
GO:0060219	camera-type eye photoreceptor cell differentiation	1	171	4	16775	0.040159	0.161293	24.5248538	1.396214	down
GO:2001168	positive regulation of histone H2B ubiquitination	1	171	4	16775	0.040159	0.161293	24.5248538	1.396214	down
GO:2001181	positive regulation of interleukin-10 secretion	1	171	4	16775	0.040159	0.161293	24.5248538	1.396214	down
GO:0051387	egative regulation of neurotrophin TRK receptor signaling pathwa	1	171	4	16775	0.040159	0.161293	24.5248538	1.396214	down
GO:0038127	ERBB signaling pathway	1	171	4	16775	0.040159	0.161293	24.5248538	1.396214	down
GO:0021747	cochlear nucleus development	1	171	4	16775	0.040159	0.161293	24.5248538	1.396214	down
GO:0060279	positive regulation of ovulation	1	171	4	16775	0.040159	0.161293	24.5248538	1.396214	down
GO:0001915	negative regulation of T cell mediated cytotoxicity	1	171	4	16775	0.040159	0.161293	24.5248538	1.396214	down
GO:0001821	histamine secretion	1	171	4	16775	0.040159	0.161293	24.5248538	1.396214	down
GO:0071922	regulation of cohesin localization to chromatin	1	171	4	16775	0.040159	0.161293	24.5248538	1.396214	down
GO:0000727	double-strand break repair via break-induced replication	1	171	4	16775	0.040159	0.161293	24.5248538	1.396214	down
GO:0051048	negative regulation of secretion	1	171	4	16775	0.040159	0.161293	24.5248538	1.396214	down
GO:0030174	regulation of DNA-dependent DNA replication initiation	1	171	4	16775	0.040159	0.161293	24.5248538	1.396214	down
GO:0001845	phagolysosome assembly	1	171	4	16775	0.040159	0.161293	24.5248538	1.396214	down

GO:0090403	oxidative stress-induced premature senescence	1	171	4	16775	0.040159	0.161293	24.5248538	1.396214	down
GO:2000553	positive regulation of T-helper 2 cell cytokine production	1	171	4	16775	0.040159	0.161293	24.5248538	1.396214	down
GO:0045213	neurotransmitter receptor metabolic process	1	171	4	16775	0.040159	0.161293	24.5248538	1.396214	down
GO:0048863	stem cell differentiation	2	171	32	16775	0.041962	0.167984	6.13121345	1.377146	down
GO:0006006	glucose metabolic process	4	171	129	16775	0.04307	0.17051	3.041842332	1.365824	down
GO:0008283	cell proliferation	8	171	385	16775	0.043906	0.17051	2.038429407	1.357476	down
GO:0031623	receptor internalization	2	171	33	16775	0.044376	0.17051	5.945419103	1.352849	down
GO:0050873	brown fat cell differentiation	2	171	33	16775	0.044376	0.17051	5.945419103	1.352849	down
GO:0046034	ATP metabolic process	2	171	34	16775	0.046841	0.17051	5.770553836	1.329372	down
GO:0009117	nucleotide metabolic process	2	171	34	16775	0.046841	0.17051	5.770553836	1.329372	down
GO:0071260	cellular response to mechanical stimulus	3	171	79	16775	0.046891	0.17051	3.725294248	1.328909	down
GO:0051291	protein heterooligomerization	3	171	80	16775	0.048366	0.17051	3.67872807	1.315462	down
GO:0007173	epidermal growth factor receptor signaling pathway	7	171	324	16775	0.048506	0.17051	2.11943181	1.3142	down
GO:0050918	positive chemotaxis	2	171	35	16775	0.049355	0.17051	5.605680869	1.306668	down
GO:0042177	negative regulation of protein catabolic process	2	171	35	16775	0.049355	0.17051	5.605680869	1.306668	down
GO:0032496	response to lipopolysaccharide	5	171	195	16775	0.049596	0.17051	2.515369621	1.30455	down
GO:0030947	lation of vascular endothelial growth factor receptor signaling patl	1	171	5	16775	0.049946	0.17051	19.61988304	1.3015	down
GO:0003100	regulation of systemic arterial blood pressure by endothelin	1	171	5	16775	0.049946	0.17051	19.61988304	1.3015	down
GO:0071482	cellular response to light stimulus	1	171	5	16775	0.049946	0.17051	19.61988304	1.3015	down
GO:1902510	regulation of apoptotic DNA fragmentation	1	171	5	16775	0.049946	0.17051	19.61988304	1.3015	down
GO:0042908	xenobiotic transport	1	171	5	16775	0.049946	0.17051	19.61988304	1.3015	down
GO:0071447	cellular response to hydroperoxide	1	171	5	16775	0.049946	0.17051	19.61988304	1.3015	down
GO:0036016	cellular response to interleukin-3	1	171	5	16775	0.049946	0.17051	19.61988304	1.3015	down
GO:0042531	positive regulation of tyrosine phosphorylation of STAT protein	1	171	5	16775	0.049946	0.17051	19.61988304	1.3015	down
GO:0051005	negative regulation of lipoprotein lipase activity	1	171	5	16775	0.049946	0.17051	19.61988304	1.3015	down
GO:0008626	granzyme-mediated apoptotic signaling pathway	1	171	5	16775	0.049946	0.17051	19.61988304	1.3015	down
GO:0010739	positive regulation of protein kinase A signaling	1	171	5	16775	0.049946	0.17051	19.61988304	1.3015	down
GO:1900180	regulation of protein localization to nucleus	1	171	5	16775	0.049946	0.17051	19.61988304	1.3015	down
GO:0042518	negative regulation of tyrosine phosphorylation of Stat3 protein	1	171	5	16775	0.049946	0.17051	19.61988304	1.3015	down
GO:0010659	cardiac muscle cell apoptotic process	1	171	5	16775	0.049946	0.17051	19.61988304	1.3015	down
GO:0006172	ADP biosynthetic process	1	171	5	16775	0.049946	0.17051	19.61988304	1.3015	down
GO:0010637	negative regulation of mitochondrial fusion	1	171	5	16775	0.049946	0.17051	19.61988304	1.3015	down
GO:0060708	spongiotrophoblast differentiation	1	171	5	16775	0.049946	0.17051	19.61988304	1.3015	down
GO:0030240	skeletal muscle thin filament assembly	1	171	5	16775	0.049946	0.17051	19.61988304	1.3015	down
GO:0030072	peptide hormone secretion	1	171	5	16775	0.049946	0.17051	19.61988304	1.3015	down
GO:0086011	membrane repolarization during action potential	1	171	5	16775	0.049946	0.17051	19.61988304	1.3015	down
GO:0046834	lipid phosphorylation	1	171	5	16775	0.049946	0.17051	19.61988304	1.3015	down
GO:0042482	positive regulation of odontogenesis	1	171	5	16775	0.049946	0.17051	19.61988304	1.3015	down
GO:0002819	regulation of adaptive immune response	1	171	5	16775	0.049946	0.17051	19.61988304	1.3015	down
GO:0033314	mitotic DNA replication checkpoint	1	171	5	16775	0.049946	0.17051	19.61988304	1.3015	down
GO:0048633	positive regulation of skeletal muscle tissue growth	1	171	5	16775	0.049946	0.17051	19.61988304	1.3015	down
GO:0090188	negative regulation of pancreatic juice secretion	1	171	5	16775	0.049946	0.17051	19.61988304	1.3015	down
GO:0090184	positive regulation of kidney development	1	171	5	16775	0.049946	0.17051	19.61988304	1.3015	down
GO:0032264	IMP salvage	1	171	5	16775	0.049946	0.17051	19.61988304	1.3015	down
GO:0060956	endocardial cell differentiation	1	171	5	16775	0.049946	0.17051	19.61988304	1.3015	down
GO:0090383	phagosome acidification	1	171	5	16775	0.049946	0.17051	19.61988304	1.3015	down
GO:0002377	immunoglobulin production	1	171	5	16775	0.049946	0.17051	19.61988304	1.3015	down
GO:0061591	calcium activated galactosylceramide scrambling	1	171	5	16775	0.049946	0.17051	19.61988304	1.3015	down
GO:0061590	calcium activated phosphatidylcholine scrambling	1	171	5	16775	0.049946	0.17051	19.61988304	1.3015	down
GO:0060075	regulation of resting membrane potential	1	171	5	16775	0.049946	0.17051	19.61988304	1.3015	down
GO:0051135	positive regulation of NK T cell activation	1	171	5	16775	0.049946	0.17051	19.61988304	1.3015	down
GO:0010803	regulation of tumor necrosis factor-mediated signaling pathway	1	171	5	16775	0.049946	0.17051	19.61988304	1.3015	down
GO:0051344	negative regulation of cyclic-nucleotide phosphodiesterase activity	1	171	5	16775	0.049946	0.17051	19.61988304	1.3015	down
GO:0048305	immunoglobulin secretion	1	171	5	16775	0.049946	0.17051	19.61988304	1.3015	down
GO:0034392	negative regulation of smooth muscle cell apoptotic process	1	171	5	16775	0.049946	0.17051	19.61988304	1.3015	down
GO:0002573	myeloid leukocyte differentiation	1	171	5	16775	0.049946	0.17051	19.61988304	1.3015	down
GO:0002551	mast cell chemotaxis	1	171	5	16775	0.049946	0.17051	19.61988304	1.3015	down
