# Supplementary material for: lncRNA HHIP-AS1 Promotes the Osteogenic Differentiation Potential and Inhibits the Migration Ability of Periodontal Ligament Stem Cells
Source: Stem Cells Int. 2021 Apr 27;2021:5595580. doi: 10.1155/2021/5595580 (PMC8554619; doi:10.1155/2021/5595580)
Supplement: Supplementary 7 — Table S5: the significant upregulated and downregulated pathways in HHIP-AS1-depleted PDLSCs. [file 5595580.f7.pdf]

Supplementary Table 5. The significant upregulated and downregulated pathways in HHIP-AS1-depleted PDLSCs.

PathwayID	PathwayTerm	DifGene	AllDifGene	GeneInPathway	AllGene	P-Value	FDR	Enrichment	(-log10P)	Style
PATH:04512	ECM-receptor interaction	19	160	89	6719	1.34E-13	2.24E-11	8.964957865	12.874245	up
PATH:04974	Protein digestion and absorption	15	160	89	6719	1.94E-09	1.63E-07	7.077598315	8.713029	up
PATH:05146	Amoebiasis	15	160	109	6719	3.41E-08	1.91E-06	5.778956422	7.4667047	up
PATH:04510	Focal adhesion	20	160	207	6719	7.68E-08	3.23E-06	4.05736715	7.1145597	up
PATH:04151	PI3K-Akt signaling pathway	22	160	346	6719	2.07E-05	0.000694	2.670122832	4.6850756	up
PATH:04610	Complement and coagulation cascades	9	160	69	6719	3.28E-05	0.000918	5.477445652	4.4842141	up
PATH:04611	Platelet activation	10	160	131	6719	0.001075	0.025804	3.205629771	2.9685209	up
PATH:05150	Staphylococcus aureus infection	6	160	53	6719	0.001511	0.031727	4.754009434	2.8207891	up
PATH:05200	Pathways in cancer	17	160	330	6719	0.002095	0.039106	2.163314394	2.6788264	up
PATH:00410	beta-Alanine metabolism	4	160	30	6719	0.005237	0.087985	5.599166667	2.2809018	up
PATH:00120	Primary bile acid biosynthesis	3	160	17	6719	0.00705	0.107672	7.410661765	2.1518132	up
PATH:04020	Calcium signaling pathway	10	160	185	6719	0.012614	0.176597	2.269932432	1.899144	up
PATH:00380	Tryptophan metabolism	4	160	41	6719	0.015818	0.189817	4.09695122	1.8008464	up
PATH:00350	Tyrosine metabolism	4	160	41	6719	0.015818	0.189817	4.09695122	1.8008464	up
PATH:05222	Small cell lung cancer	6	160	89	6719	0.019019	0.198392	2.831039326	1.7208228	up
PATH:02010	ABC transporters	4	160	44	6719	0.020075	0.198392	3.817613636	1.697337	up
PATH:00071	Fatty acid metabolism	4	160	44	6719	0.020075	0.198392	3.817613636	1.697337	up
PATH:00982	Drug metabolism - cytochrome P450	5	160	73	6719	0.029503	0.275365	2.876284247	1.5301284	up
PATH:05144	Malaria	4	160	52	6719	0.034626	0.306169	3.230288462	1.460595	up
PATH:05205	Proteoglycans in cancer	10	160	225	6719	0.041834	0.333747	1.866388889	1.3784727	up
PATH:00980	Metabolism of xenobiotics by cytochrome P450	5	160	81	6719	0.04334	0.333747	2.59220679	1.3631095	up
PATH:04340	Hedgehog signaling pathway	4	160	56	6719	0.043705	0.333747	2.999553571	1.3594697	up
PATH:05143	African trypanosomiasis	3	160	34	6719	0.046276	0.338014	3.705330882	1.3346463	up
PATH:00604	Glycosphingolipid biosynthesis - ganglio series	2	160	15	6719	0.048301	0.338105	5.599166667	1.3160464	up
PATH:05134	Legionellosis	7	90	55	6719	7.36E-06	0.000828	9.501616162	5.1331333	down
PATH:05322	Systemic lupus erythematosus	10	90	134	6719	1.05E-05	0.000828	5.571310116	4.9793897	down
PATH:05202	Transcriptional misregulation in cancer	11	90	180	6719	2.48E-05	0.001307	4.562283951	4.6052404	down
PATH:05034	Alcoholism	10	90	179	6719	0.000127	0.005	4.170701428	3.8976271	down
PATH:04060	Cytokine-cytokine receptor interaction	12	90	263	6719	0.000181	0.005712	3.406337136	3.7429293	down
PATH:04668	TNF signaling pathway	7	90	110	6719	0.000636	0.016751	4.750808081	3.1964621	down
PATH:04630	Jak-STAT signaling pathway	8	90	157	6719	0.001137	0.025549	3.804104742	2.9443802	down
PATH:04110	Cell cycle	7	90	124	6719	0.001294	0.025549	4.214426523	2.8881991	down
PATH:05219	Bladder cancer	4	90	42	6719	0.002285	0.040107	7.11005291	2.6411908	down
PATH:05323	Rheumatoid arthritis	5	90	88	6719	0.006311	0.099719	4.241792929	2.199881	down
PATH:04068	FoxO signaling pathway	6	90	133	6719	0.008603	0.123564	3.367919799	2.0653725	down
PATH:05200	Pathways in cancer	10	90	330	6719	0.012373	0.152813	2.262289562	1.9075117	down
PATH:04115	p53 signaling pathway	4	90	68	6719	0.012776	0.152813	4.391503268	1.8935952	down
PATH:04066	HIF-1 signaling pathway	5	90	106	6719	0.01354	0.152813	3.52148847	1.8683682	down
PATH:05146	Amoebiasis	5	90	109	6719	0.015132	0.159389	3.424566769	1.8201088	down
PATH:04730	Long-term depression	4	90	73	6719	0.016238	0.160349	4.090715373	1.7894711	down
PATH:05203	Viral carcinogenesis	7	90	206	6719	0.020069	0.18652	2.536839266	1.6974829	down
PATH:04062	Chemokine signaling pathway	6	90	188	6719	0.039758	0.348983	2.382624113	1.4005809	down
PATH:04916	Melanogenesis	4	90	101	6719	0.046047	0.382915	2.956655666	1.3368008	down
PATH:04621	NOD-like receptor signaling pathway	3	90	62	6719	0.0499	0.394214	3.612365591	1.3018955	down
